# Local urban government policies to facilitate healthy and environmentally sustainable diet-related practices: a scoping review

**DOI:** 10.1017/S1368980021004432

**Published:** 2022-02

**Authors:** Liza Barbour, Rebecca Lindberg, Julie Woods, Karen Charlton, Julie Brimblecombe

**Affiliations:** 1 Department of Nutrition, Dietetics & Food, Monash University, Level 1, 264 Ferntree Gully Road, Notting Hill, VIC 3168, Australia; 2 Institute for Physical Activity and Nutrition (IPAN), School of Exercise and Nutrition Sciences, Deakin University, Geelong, VIC, Australia; 3 Smart Foods Centre, School of Medicine, University of Wollongong and Illawarra Health and Medical Research Institute, Wollongong, NSW, Australia

**Keywords:** Planetary health, Public health, Ecological nutrition, Public policy, Food systems

## Abstract

**Objective:**

This scoping review sought to describe the policy actions that urban local governments globally have implemented to facilitate healthy and environmentally sustainable diet-related practices.

**Setting:**

Urban local government authorities.

**Design:**

Five databases were searched to identify publications which cited policies being implemented by local governments within the 199 signatory cities of the Milan Urban Food Policy Pact (MUFPP) that targeted at least one healthy and sustainable diet-related practice. Grey literature was then searched to retrieve associated policy documentation. Data from both sources were charted against the MUFPP’s monitoring framework to analyse the policy actions included in each overarching policy.

**Results:**

From 2624 screened peer-reviewed studies, 27 met inclusion criteria and cited 36 relevant policies amongst signatory cities to the MUFPP. Most were from high income countries (*n* 29; 81 %), considered health (*n* 31; 86 %), equity (*n* 29; 81 %) and the broader food system beyond dietary consumption (*n* 34; 94 %). Of the 66 policy actions described, the most common involved food procurement within public facilities (*n* 16; 44 %) and establishing guidelines for school-feeding programs (*n* 12; 33 %).

**Conclusions:**

This review has demonstrated that urban local government authorities are implementing policies that consider multiple phases of the food supply chain to facilitate population-wide uptake of healthy and sustainable diet-related practices. Opportunities exist for local governments to leverage the dual benefits to human and planetary health of policy actions, such as those which discourage the overconsumption of food including less meat consumption and the regulation of ultra-processed foods.

Our planet’s ability to sustain human life into the future is under immediate threat. The global food system is creating more greenhouse gas emissions than any other single contributor, depleting natural resources such as land and water, and driving biodiversity loss^(1,2,3,4)^. The EAT-Lancet Commission advised that to nourish a growing population within planetary boundaries, ‘nothing less than a Great Food Transformation’ is required, including a global shift towards healthy and sustainable diets^(5)^, p448. To achieve planetary health, that being ‘the health of human civilisation and the state of the natural systems on which it depends’^(6)^ p1978, effective governance at global, national and local levels is critical^(7,8,9)^. At a national level, governments have committed to meet targets set within the UN Sustainable Development Goals^(10)^, the Paris Climate Agreement^(11)^ and the Decade for Action on Nutrition^(12)^. These national commitments require local action, whereby ‘municipalities – with their close connections to residents, local businesses and civil society organisations – are key to the implementation of most SDG’^(13)^, p10.

By 2030, it is anticipated that 60% of the global population will live in urban areas^(14)^. It has been argued that local authorities which govern urban cities have a role to play in this food system transformation^(3,7)^. Hosted in Milan (Italy), the World Expo 2015, brought together government stakeholders, civil society, non-government organisations and corporations to determine how the world can sustainably nourish a growing population^(15)^. In the lead up to this event, the Mayor of Milan led a dialogue on the role of urban cities to achieve this, amongst leaders from 46 cities and an advisory group representing relevant international organisations and scientific experts^(15)^. This resulted in the development of the Milan Urban Food Policy Pact (MUFPP), the first International guide on urban food policies, which was launched at the World Expo 2015^(16)^. Delegates from over 100 cities globally signed the Pact, publicly committing to developing sustainable food systems that are: (i) inclusive, resilient, safe and diverse; (ii) provide healthy and affordable food to all people in a human rights-based framework and (iii) minimise waste and conserve biodiversity while adapting to and mitigating impacts of climate change^(16,17)^. A review conducted by Candel (2019) identified that approximately one-quarter (*n* 23) of MUFPP signatory cities had developed local food policies^(18)^. His review described the commonalities and differences between food policies designed to affect dietary consumption by targeting any phase of the food supply chain, from food production to waste^(18)^. Missing from the literature, however, is a comprehensive description of relevant policies and their associated policy actions which specifically target the consumption phase of the food supply chain, as a component of the broader food system. With local government authorities proposed as critical stakeholders in shifting population diets, this review provides valuable insight as to where current policy action is being focused and where gaps exist^(7,16)^.

Conducted through a public health nutrition lens, this scoping review was undertaken to document policies cited in the peer-reviewed literature that local governments within MUFPP signatory cities have implemented since 2015, to target desirable healthy and sustainable diet-related practices.

## Methodology

A 5-staged approach for scoping reviews^(19,20,21)^ was used with reporting conducted according to Preferred Reporting Items for Systematic reviews and Meta-Analyses extension for Scoping Reviews guidelines^(22,23)^. Scoping review methodology was used due to the broad nature of the research question and to allow for an emerging body of research to be explored and evidence from diverse sources to be included, regardless of quality^(22,24)^. A review protocol was registered with the Open Science Framework^(25)^.

To explain the sequential approach taken for this review, an understanding of the following terminology is required. ‘*Policy’* refers to the over-arching planned approach to achieve pre-determined, desired outcomes. ‘*Policy actions*’ refer to the activities included in the strategic plan developed in order to achieve the overarching policy objectives. ‘*Healthy and sustainable diet-related practices’* refer to the specific activities that an individual engages in to source, store, prepare, consume and dispose of the food that makes up their overall diet. For example, a municipality may have a Local Food System Strategy (*policy*), which includes a community garden (*policy action*) to promote increased consumption of locally grown fruit and vegetables (*diet-related practice*).

### Stage 1: identifying the research questions

To describe the types of policy that local governments globally are implementing to promote the uptake of healthy and environmentally sustainable diet-related practices, these sub-questions were considered: (1) Which healthy and sustainable diet-related practices are local governments targeting? (2) What is the relationship between the broader geographical, economic and political context and the rationale for the type of policy that has been implemented? (3) Are these policies part of a broader intervention? (4) Is health, equity and the broader food system considered? If so, how? (5) Is evaluation of these policies being planned for? If so, how? Is evidence used in the design and implementation of these policies? and (6) If so, how?

### Stage 2: identifying relevant studies for inclusion

#### Determining healthy and sustainable diet-related practices

In 2012, the Food and Agriculture Organisation defined healthy and sustainable diets as those with ‘low environmental impacts which contribute to food and nutrition security and to healthy life for present and future generations’^(26)^, p7. Healthy and sustainable population-level reference diets and recommendations to inform dietary guidelines have since been derived from the large body of evidence^(5,27)^. However, for the purpose of this review, a comprehensive list of specific diet-related practices for policy-makers to target was required. As this did not exist in the literature, these were defined by the authors and reported elsewhere^(28)^. Thirteen diet-related practices were identified and categorised (Table 1) to describe where to source food, what foods to eat and how to consume foods as part of a healthy and sustainable diet. See also *Supplemental Material S1: Commonly cited healthy and environmentally sustainable diet-related practices* and *Supplemental Material S2: Description of each healthy and sustainable diet-related practice.*

**Table 1:** Individual-level practices required to achieve healthy and sustainable diets^(28)^

Where to source food?
• Select food grown using sustainable food production practices, valuing and respecting Indigenous knowledges
• Strengthen local food systems by connecting with primary producers
• Eat seasonally, incorporating native and wild-harvested foods
• Eat locally available foods
What to eat?
• Avoid over-consumption beyond caloric requirement
• Consume no more than recommended animal-derived foods
• Limit intake of ultra-processed, nutrient-poor and over-packaged food
• Increase intake of plant-based foods
• Eat a wide variety of foods to promote biodiversity
How to eat?
• Adopt food waste-minimisation strategies
• Preference home-made meals and share with others
• Consume safe tap water as preferred drink
• Breastfeed infants where possible

#### Search strategy for identifying studies

Five databases (Scopus, Medline, CINAHL Plus, Global Health and Pro-quest – Agricultural & Environmental Science Collection), were searched on July 29, 2019, filtering for papers published in English after 2015, when the MUFPP was established. The search combined the following terms with their synonyms (*Supplemental Material S3: Search Terms for peer-reviewed publication search*); ‘local government’ (local, municipal*, county, counties, shire*, provin*, regional, city, town, urban, metropolitan, council, authorit*, govern*, board*, service*, office*) AND ‘policy’ (policies, act, strategy*, plan*, scheme, initiative*, intervention*, program*, action*, law, legislat*, guideline*, regulat*) AND ‘food’ (diet) AND ‘environmental sustainability’ (green, sustainab*, greenhouse gas, carbon emissions, GHG, climate, unsustainab*, enviro*, ecolog*, health*, eco-friendly*). Results were exported via Endnote into Covidence software to facilitate collaborative screening.

#### Criteria for selecting relevant studies

To meet inclusion criteria for this review (Table 2), studies had to describe policies implemented by local-level governments within signatory cities of the MUFPP (*n* 199 cities as of 1st August 2019), focus on urban settings and be published after 2015. Restricting the sample to only signatory cities of the MUFPP was done to restrict the size of this study and to refine the scope by identifying policies from cities that had publicly committed to improving both health and sustainability outcomes of their local food system. Hawkes and Halliday’s (2017) holistic definition was adopted to inform the inclusion criteria, whereby they define urban food policy as ‘a concerted action on the part of city government to address food-related challenges… grassroots, citizen-led actions that are independent of governments do not constitute urban food policies per se’^(29)^, p9. For the purpose of this review, this definition of urban food policy was adopted whereby single-issue policies as well as multi-faceted policies, often with an integrated, holistic approach beyond food, were considered.

**Table 2:** Inclusion criteria for publications citing relevant policies

Criterion	Definition
Policy	Policy included any plan, action, intervention, initiative, activity or strategy which had pre-determined intentions (goals, objectives, targets) accompanied by a planned approach or work plan to achieve the desired outcome. Ad hoc activities were not included unless they were part of a policy. Policies could be documented in many forms such as regulatory or non-regulatory statements, websites and strategic reports. Hypothetical scenarios such as simulation or modelling were not included. Food Policy Councils were included, provided they were initiated by local government (or have significant involvement)
Outcome	The intended outcome of the policy must have included the promotion of at least one healthy and sustainable diet-related practice, as outlined in Table 1. The targeted diet-related practice(s) must have been clearly stated. The policy must have been designed with consideration of environmental sustainability therefore policies aiming to address overweight, obesity, food insecurity, veganism, vegetarianism or cancer were not included unless environmental sustainability outcomes were considered explicitly. Policies promoting urban agriculture, food safety and sustainable farming were not included unless the desired diet-related practice was considered explicitly. Urban agriculture policies which described the intention to increase dietary consumption of locally grown, seasonal and/or plant-based foods were eligible for inclusion
Local government involvement	The policy must have been implemented at a local government level and involve local government employees as stakeholders. Involvement could range from lead implementer, funding provision or consultation representative. The terminology used for local government varies and included; county, municipality, local government area, province, shire, region, council, office
Settings	The policy must have been implemented in an urban setting, specifically in one of the MUFPP signatory cities (*n* 199 as documented on 22 July 2019)
Study	The publication must have been available in English, published in or after 2015, include adequate detail to discern relevance. Any study type – reviews, dissertations, conference proceedings, etc – was considered

### Stage 3: selection of included studies

Two researchers (Authors 1 and 5) completed the screening of titles and abstracts, with a third (Author 3) engaged to resolve discrepancies. Full-text papers were retrieved (Author 1) and each assessed against the inclusion criteria by 2 researchers (Author 1 and Authors 2, 3 and 5). Data extraction using Microsoft Excel was piloted via double-extraction (Author 1 and Authors 3, 4 and 5) with a subset of 6 papers to check for accuracy and consistency. As per the non-linear and iterative nature of scoping review methodology, the data extraction method was refined based on consultation throughout this double-extraction process^(19)^. The lead researcher (Author 1) completed data extraction on all items with fortnightly review (Authors 1, 3 and 5) to further refine the extraction process and inclusion criteria to ensure included studies enabled the research questions to be answered comprehensively.

### Stage 4: charting the data from peer-reviewed publications and policy documentation

To answer the research questions, a 3-staged process was followed to chart the data:

- Data extraction from included peer-reviewed publications; year, authors, title, citation, study aim, study design, targeted population, cited policy(s), MUFPP signatory city and key findings.
- Retrieval of associated policy documents from the grey literature (e.g. policy documents, legislation, case studies, websites) cited by publications identified in step one. The policy title was entered into Google (incognito mode) and the first 10 results were scanned to find the documentation that was most relevant and recent, the most primary source (media commentary was excluded) and reported in English (or translation provided). Retrieval of cited policy documents was conducted between January and April 2020; and
- Data were extracted from the retrieved policy documents, as follows: name and location of policy, MUFPP signatory city(s), related policies, policy aim, description of policy actions, role of local government, targeted diet-related practice(s), category(s) of action according to the MUFPP Monitoring Framework^(13)^, consideration of health, equity and the broader food system, planned evaluation, effectiveness of the policy and process for integrating evidence into policy development. Where detail was missing from the policy documents, the peer-reviewed publication from step one was reviewed to populate the spreadsheet.

### Stage 5: collating, summarising and reporting of results to identify policy actions described within the policy documents

A modified PRISMA flowchart was completed to report the sources of peer-reviewed publications^(23)^. To answer the research question, all policy actions described within the policy documents were identified and mapped against the MUFPP’s Framework of Action^(17)^. This framework, developed by the Food and Agriculture Organisation in consultation with signatory cities, is organised into 6 categories^(13,17)^. Directed content analysis^(30)^ was used to categorise each of the policy action. Descriptive analysis was then used to derive a count of frequency, geographic and economic context, and targeted diet-related practice from the charted data. All authors approved final categorisation and reporting of results.

## Results

**Fig. 1:**
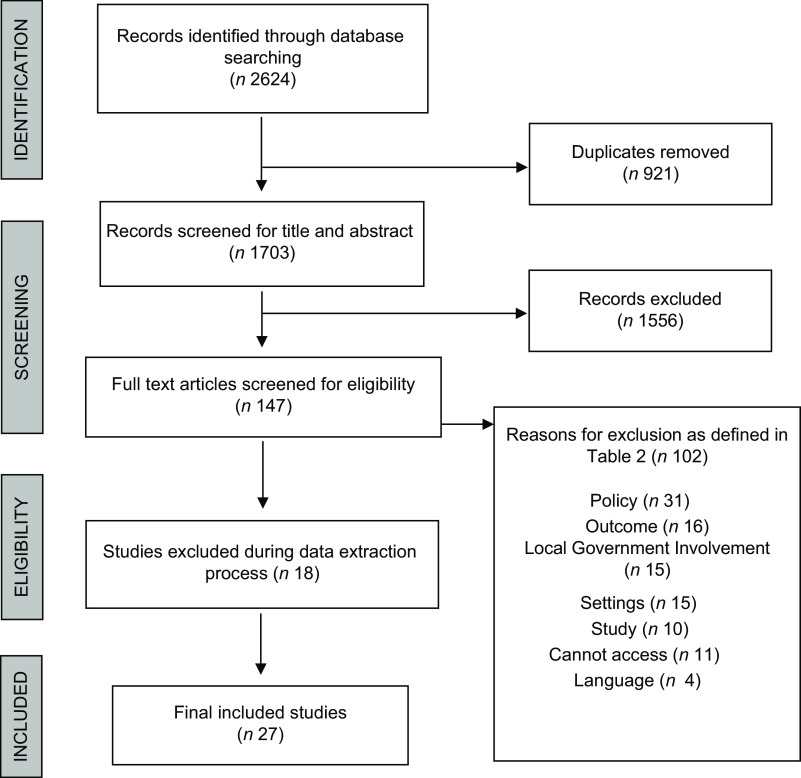
PRISMA flowchart of literature search and selection of inclusion process^(23)^

The original database search yielded 2624 results, from which 147 remained after removing duplicates and screening the title and abstract (Fig. 1). During the full-text screening, 102 were removed as they did not meet inclusion criteria as described in Table 2. A further 18 studies were excluded during the data extraction process as inadequate detail was provided about the setting (e.g. the geographical region may have been stated however the specific signatory city not mentioned), the policy (e.g. the name of the specific policy is not provided to allow retrieval of the policy documentation) or the outcome (e.g. the policy may have led to desired healthy and sustainable dietary outcomes however environmental sustainability was not described as a consideration). A resulting 27 studies were included.

Of the included studies (*n* 27)^(29,31,32,33,34,35,36,37,38,39,40,41,42,43,44,45,46,47,48,49,50,51,52,53,54,55,56)^ most applied qualitative research methods (*n* 21) and used either content, document or policy analysis (*n* 16), interviews (*n* 11) or case studies (*n* 10) to address study aims (see online Supplemental Materials S4: Summary *characteristics of included studies* and S5: *Data extraction from included studies*). Most studies targeted local government stakeholders such as leaders and city planners (*n* 21), while approximately one-third included stakeholders from other levels of government (*n* 9) and several engaged primary producers of food (*n* 3).

### Characteristics of relevant policies cited within included studies

Thirty-six policies which met inclusion criteria were cited in these studies (see online *Supplemental Material S6: Data extraction from policy documents*). Most policies were implemented in cities within a high-income country (*n* 29; 81 %), with the majority in Europe and Central Asia (*n* 24; 67 %) (Table 3). Most policies described the role of local government as one of leadership or ownership (*n* 33; 92 %), rather than merely consultative (*n* 2; 6 %) or as a funding source (*n* 1; 3 %). Example policies include ‘Ghent en Garde’, a local food policy (Belgium), the ‘San Francisco Zero Waste’ commitment (USA) and the Nairobi Urban Agriculture Promotion and Regulation Act (Kenya).

**Table 3:** Characteristics of policies cited in included studies

Characteristic	Policies (*n* 36)*	
Signatory cities cited – economic context as per World Bank categorisation		
Low income	0	0 %
Lower-middle income	2	6 %
Upper-middle income	5	14 %
High income	29	81 %
Signatory cities cited – geographic context as per World Bank categorisation		
East Asia and Pacific	0	0 %
Europe and Central Asia	24	67 %
Austria (Vienna), Belgium (Ghent), Denmark (Copenhagen), France (Paris *n* 3), Italy (Catania, Bologna *n* 2, Rome *n* 2, Turin, Milan, Ancona, Bari & Cagliari), Netherlands (Rotterdam), Spain (Barcelona *n* 2), Switzerland (Zurich), UK (Bristol, Brighton & Hove, London *n* 2)		
Latin America and Caribbean	10	28 %
Brazil (Araraquara, Belo Horizonte *n 2*, Curitiba, Porto Alegre, Praia, Rio de Janeiro, Sao Paulo), Columbia (Medellin), Ecuador (Quito)		
Middle East and North Africa	0	0 %
North America	10	28 %
Canada (Toronto *n* 3, Vancouver), United States of America (New York City *n* 2, Chicago *n* 2, San Francisco *n* 2)		
South Asia	0	0 %
Sub-Saharan Africa		
Kenya (Nairobi), Senegal (Dakar), South Africa (Cape Town)	3	8 %
Role of local government		
Stakeholder consultation/representation	2	6 %
Leadership/ownership (development, implementation & evaluation)	33	92 %
External funding body, award recognition body	1	3 %
Targeted healthy and sustainable diet-related practices		
Where to source food		
Select food grown using sustainable food production practices, valuing Indigenous knowledges	31	83 %
Strengthen local food systems by connecting with primary producers	28	78 %
Eat seasonally, incorporating native and wild-harvested foods	29	81 %
Eat locally available foods	28	78 %
What to eat		
Avoid over-consumption beyond caloric requirement	12	33 %
Consume no more than recommended animal-derived foods	10	28 %
Limit intake of ultra-processed, nutrient-poor and over-packaged food	10	28 %
Increase intake of plant-based foods	26	72 %
Eat a wide variety of foods to promote biodiversity	17	47 %
How to eat		
Adopt food waste-minimisation strategies	23	64 %
Preference home-made meals and share with others	8	22 %
Consume safe tap water as preferred drink	9	25 %
Breastfeed infants where possible	3	8 %
Targeted phase of the food supply chain†		
Agricultural production	33	92 %
Distribution, transport and trade	26	72 %
Processing	12	33 %
Food retail/service	16	44 %
Consumption	35	97 %
Waste and disposal	21	58 %
More than one phase considered	34	94 %
All considered	8	22 %
Considerations during policy development and implementation		
Health	31	86 %
Equity	29	81 %
Broader food system	34	94 %
Evaluation plans	30	83 %
Use of evidence in development		
Reported in policy document or included study(s)		
Type of evidence used	22	61 %
Process for integrating evidence	23	64 %

* Citation for each policy (*n* 36) is available in Supplemental Material S6: Data extraction from policies documents.

† The food supply chain is characterised by a series of activities, categorised as: (i) agricultural production; (ii) distribution, transport and trade; (iii) processing; (iv) food retail/service; (v) consumption and (vi) waste and disposal^(57)^. This supply chain sits within the broader food system, defined as ‘the interconnected system of everything and everybody that influence, and is influenced by, the activities involved in bringing food from farm to fork and beyond’^(57)^, p1.

Within these policies, the most commonly targeted healthy and sustainable diet-related practices were those within the category of *where to source food* (*n* 28–31; 78 %–83 %), with *increasing intake of plant-based foods* (*n* 26; 72 %), in the *what to eat* category and *adopting waste minimisation strategies* (*n* 23; 64 %) in the *how to eat* category, also targeted frequently. Some policies targeted action across all 6 phases of the food supply chain (*n* 8; 22 %), or at least 2 phases (*n* 34; 94 %), while few (*n* 2, 6 %) focused on just one phase^(57)^. The majority of policies considered health (*n* 31; 86 %), equity (*n* 29; 81 %) and the broader food system, beyond either the consumption phase of the supply chain or the local government’s geographic boundaries (*n* 34; 94 %). Many policies described elements of evaluation (*n* 30, 84 %), such as measurable targets, partnership with research institutions or data collection tools.

Many policies failed to report on the type of evidence used to inform the policy-making processes (*n* 14; 39 %) or the process used to integrate this evidence (*n* 13; 36 %). The type of evidence described to inform the policy-making processes included government statistics, resident concern, advocacy led by community organisations and grey literature. Of the policies that did report on the way this evidence was integrated into the policy-making process, approaches ranged from public meetings, roundtable events, stakeholder workshops, formal prioritisation processes, farmer forums, women-led community consultations, surveys, speaker sessions and advisory group meetings. Individuals involved in these processes included government employees, citizens (including women, children, young people, and those disproportionately affected by obesity and poverty), primary food producers, lawyers, researchers, chefs, procurement officers, social planners, funders, gardeners and teachers. These details are available in the ‘policy development’ section of *Supplemental Material S6: Data extraction from policy documents.*

Within the 36 policies, a total of 66 policy actions were identified with actions falling into all 6 categories of the MUFPP Monitoring Framework (Table 4). The highest number of actions aligned with the food waste category (*n* 17; 26 %) and the least number of actions aligned with the food supply and distribution category (*n* 6; 9 %). The policy actions most frequently described however were in the food supply and food distribution category and involved food procurement for food service in public facilities (*n* 16; 44 %), establishing guidelines for school feeding programs (*n* 12; 33 %) and allocating urban garden plots for food production and education opportunities for people experiencing disadvantage (*n* 11; 31 %). In observing the regions, most of the 11 policy actions which targeted social and economic equity outcomes were implemented in North America (*n* 9; 82 %), compared with Europe and Central Asia (*n* 8; 73 %), Latin America and Caribbean (*n* 3, 27 %) and Sub-Saharan Africa (*n* 1; 9 %). Food waste policy actions were only identified in Europe and Central Asia (*n* 12; 70 %) and North America (*n* 6; 35 %). Not all policy actions could be linked directly to specific healthy and sustainable diet-related practices however most targeted multiple practices. Based on this analysis of policy actions, of the 13 desirable healthy and sustainable diet-related practices, those most commonly targeted were waste-minimisation practices (*n* 23; 35 %), selecting food grown using sustainable food production practices valuing and respecting Indigenous knowledges (*n* 21; 32 %), strengthening local food systems by connecting with primary producers (*n* 16; 24 %) and eating locally available foods (*n* 16; 24 %)

**Table 4:** Policy actions to promote healthy and sustainable diet-related practices

MUFPP Monitoring framework category (number of actions identified across all policies)	Brief description of policy actions included in City* policy	Number of policies reporting each action		Targeted H&S diet-related practice§
		Number (citation†)	Region‡	
Enabling effective action (governance) (*n* 12)	Join regional and global networks (e.g. C40 Cities, Edible Cities, Urban Agriculture Association) and sign relevant declarations to publicise City commitment	3^(xii,xxxiii,xxxvi)^	E, N, S	N/A
	Collaborate with academic institutions to support grass-roots initiatives with best-practice approaches to conceive and co-design ideas, implement activities and plan and conduct evaluation	1^(viii)^	E	N/A
	Fund prizes to recognise and promote grass-roots urban agriculture initiatives	1^(iv)^	E	N/A
	Establish and support city-wide Food Policy Councils to drive policy action	5^(ii,xvi,xxiv,xxx,xxxii)^	E, N	N/A
	Establish annual public events (e.g. Sustainable Food Day, Urban Agriculture Week) to demonstrate political commitment, celebrate innovative initiatives and engage City Councillors, media and City staff on a tour to meet project participants	2^(v,xxiv)^	E, N	N/A
	Create a food charter to define key principles of practice around health and sustainability, to enable partnerships between stakeholders with common values and aspirations	2^(iii,xvi)^	E	N/A
	Establish a ‘Network of actors of sustainable food’ via a digital platform and directory to connect projects and suppliers supporting sustainable food	1^(v)^	E	N/A
	Establish a producers’ network with stakeholders from neighbouring councils to ensure consistency of approach and enable urban, peri-urban and rural linkages	2^(xvi,xxv)^	E, N	N/A
	Mandate food standards requiring public facilities‖ and contractors to serve food and beverages which meet nutrition and sustainability outcomes, including vending machines and drinking fountains	3^(xxix,xxxi,xxxii)^	N	N/A
	Involve professionals with technical nutrition and sustainability expertise in planning decisions to facilitate evidence-based recommendations (e.g. include urban agriculture in planning processes for land use, ensure new buildings have bottle-filling stations, avoid obesogenic environments via Health Impact Assessment processes, zoning, food policy, marketing and market infrastructure)	4^(v,xxix,xxxiv,xxxvi)^	E, N, S	N/A
	Expand and support new food enterprises by including one or more of these strategies: - funding incubator programs - appointing a senior City official to assist farming and food entrepreneurs to navigate approval and regulatory processes - offering target tax increment financing to incentivise businesses involved in local production, processing and distribution of healthy food (e.g. long-term land rental agreements) - enhancing healthy food retail options especially in underserved areas	3^(v,xxi,xxxv)^	N	N/A
	Ensure City representation on advisory boards to inform nutrition and environmental sustainability regulation and policy decisions	1^(xxxiv)^	S	N/A
Sustainable diets and nutrition (*n* 9)	Establish guidelines for school feeding programs which include one or more of these strategies to ensure: - staff are adequately trained in nutrition and sustainability - menu is designed to prioritise organic, local, seasonal ingredients - meat is served less than 1–2 times/week - use of environmental modes of transport for suppliers (e.g. natural gas-fuelled vehicles) - purchase of school kitchen appliances based on energy efficiency/savings criteria - fresh drinking water (refillable station – no plastic bottles) - nutrition and sustainability education are integrated into the school curriculum (e.g. food laboratories to support students with hands-on learning from local producers and processors) - awareness campaigns for the broader school community	12^(ii,iii,v,vii,xi,xiv,xvii,xx,xxii,xxv,xxvii,xxix)^	E, L, N	1–12
	Support schools and early learning centres to work towards and achieve state/local government awards, which require integrated curriculum with a focus on nutrition, health and gardening	5^(xiii,xvi,xvii,xviii,xix)^	E	N/A
	Fund academic institutions’ student unions to facilitate student-led projects to promote flexitarianism and food waste reduction	1^(xvi)^	E	6, 8, 10
	Support national campaigns such as ‘Flexitarian City’, ‘Sustainable Fish Cities’ and ‘Fairtrade town’ which promote desired consumption and procurement practices in restaurants and communities throughout the city	4^(v,xvi,xix,xxxii)^	E	1, 6, 8
	Promote social prescription of motivational healthy eating and cooking courses within mainstream healthcare	1^(xvi)^	E	N/A
	Incentivise food sustainability practices in the food service setting (restaurants, catering, retail) through menu and product labeling	3^(vxxviiixxxii)^	E, N	N/A
	Invest in awareness-raising activities to reduce meat consumption and promote local, Fairtrade and seasonal produce (e.g. nutrition education, signage on farmland, social marketing campaigns, regional food brand labels)	6^(ii,v,xix,xxiii,xxvx,xvii)^	E, L, N	1, 2, 6
	Establish non-commercial foundation with chefs and technical nutrition and sustainability expertise to support kitchen staff in public facilities to prioritise desired diet-related practices (e.g. less meat, use of whole animal, increase organic, seasonal and local fresh produce, differentiate every day and feast (sweet/expensive/processed) menu items, less waste)	1^(iii)^	E	1, 3–10
	Promote desired diet-related practices through City-funded events (e.g. only serving vegetarian food at public events on Thursdays)	1^(ii)^	E	6
Social and economic equity (*n* 11)	Allocate urban garden plots for food production and education opportunities to people experiencing disadvantage (e.g. Retirees and people with disabilities, young people, people seeking asylum, reintegration and therapeutic rehabilitation projects)	11^(iv,viii,ix,xi,xiv,xvi,xxii,xxiii,xxvii,xxxv,xxxvi)^	E, N, S	1–2
	Co-design rooftop gardens with residents in social housing complexes to provide a communal space for neighbours to connect socially, learn organic food production practices and exchange knowledge, culture and experiences	2^(viii,xiv)^	E	1–2
	Build capacity amongst low-income residents and refugees by providing food handler training, nutrition education and support to secure food-related employment	6^(ii,xvii,xxi,xxiv,xxvii,xxx)^	E, L, N	N/A
	Connect emergency food relief services (including school breakfast programs) with local food producers to increase the nutritional quality of food served to people experiencing food insecurity	8^(xvi,xviii,xxi,xxv,xxvii,xxviii,xxix,xxx)^	E, L, N	4, 8
	Encourage emergency food relief and food rescue organisations to provide capacity building opportunities for people experiencing food insecurity to improve nutrition, health and sustainability outcomes (e.g. reduce household food waste)	3^(xvi,xxvii,xxx)^	E, N	10
	Advocate to raise welfare payments to enable equitable access to local, fresh and healthy food	1^(xix)^	E	4, 8
	Generate employment in the local food manufacturing sector by making affordable public space available and offering technical assistance	1^(xxx)^	N	N/A
	Fund emergency food relief services and food rescue organisations who prioritise the provision of healthy food to people experiencing food insecurity	1^(xxxii)^	N	N/A
	Innovate digital solutions to support people experiencing food insecurity to find community food opportunities and emergency food relief in the local area	1^(xxix)^	N	N/A
	Support social enterprises and programs which aim to increase access to healthy and sustainable food for people experiencing disadvantage (e.g. social solidarity stores, communitarian restaurants for elderly, vouchers for ‘at risk’ families to spend at local produce markets, ‘pay it forward’ programs where customers can pay for someone less fortunate to access food later) by facilitating access to public premises and offering financial support	4^(v,xviii,xxii,xxiii)^	E, L, N	N/A
	Establish communal kitchens in public facilities to allow people without such facilities to cook healthy, sustainable, seasonal, organic meals	1^(v)^	E	1, 3–4, 7, 11
Food production (including urban–-rural linkages) (*n* 11)	Incentivise and simplify regulations to establish urban garden plots in vacant spaces (including traffic islands) and areas destined to abandonment and degradation	7^(vi,ix,xv,xxi,xxviii,xxix,xxxvi)^	E, L, N, S	N/A
	Create rooftop, vertical and urban gardens designed, built and managed for high-yielding food production, with consideration of one or more of these concepts: - circular economy principles with composting and soil management (e.g. earthworms) - integration of urban agriculture into commercial agriculture industry by connecting gardeners with the same research bodies, markets, suppliers as commercial farmers - supplying local restaurants, public facilities and residents - a code of management to ensure public access and conditions of food production (e.g. composting, rain water recovery and use, reporting) - City expertise in landscape design - attracting City funds for raised garden beds, topsoil, water supply, above-ground bins for edibles and utility shed and other tools	8^(iv,vi,xvi,xxi,xxii,xxiii,xxxiv,xxxvi)^	E, L, N, S	1–4, 10
	Create edible garden beds at train stations and other public facilities to be maintained by local school communities and neighbourhood groups to enable social connections between elderly, children and other population sub-groups	6^(xiv,xvi,xix,xviii,xxix,xxxv)^	E, S	1–2
	Promote the vital role of primary producers to the general public (e.g. farmer visits at local schools to encourage more schools, community groups and businesses to procure Fairtrade produce)	1^(xvi)^	E	1
	Create a citizen statement to define the value of good quality soil to the City and describe how local government will demonstrate that value (e.g. dumping restrictions, healthy soil guidelines for urban farms)	2^(xvi,xxix)^	E, N	1–2
	Develop a Pollinator Strategy to promote better habitat management for insect pollinators required for food production	1^(xvi)^	E	1–2
	Maintain and improve local and fringe farmland protection programs, prioritising high-density areas and informal settlements	3^(xxvii,xxviii,xxxiv)^	N, S	1
	Facilitate sustainable practices on public property (e.g. parks providing opportunities for native plant foraging, seed saving/exchange, composting workshops)	2^(xviii,xxvii)^	E, N	1–2, 4
	Support primary producers, including urban gardeners to enjoy therapeutic, subsistence, income, employment and export outcomes from their farming by providing educational, technical and financial resources (e.g. establish educational farms for the purpose of hosting workshops)	10^(ii,v,xxii,xxiii,xxv,xxix,xxxii,xxxiv,xxxv,xxxvi)^	E, L, N, S	N/A
	Promote best-practice in food production approaches by offering financial support where required to enable one or more of these strategies: - protect animal welfare standards (e.g. prohibit use of factory farmed livestock in public facilities) - support traceability systems - increase organic farming, prohibiting pesticides and fertiliser use and sharing cost of organic certification for small-medium businesses	4^(xv,xxiii,xxxiv,xxxvi)^	E, L, S	1, 6
	Develop internships, apprenticeships and scholarships to inspire students and young people to take on careers in food and farming	1^(xxv)^	N	N/A
Food supply and distribution (*n* 6)	Implement food procurement policies for food service in public facilities by including one or more of these strategies: - Require a proportion of funds to be spent on seasonal, organic agricultural products from local family farms including fruit, vegetable, dairy products and sustainability raised and harvested seafood and locally raised and butchered meat, with employment conditions which adhere to international labour standards - Establish a tender process with criteria for applicants to demonstrate their ability to minimise health and environmental impact through a range of strategies; waste minimisation, local and organic procurement, meat labelling (e.g. origin, delivered within 4 d of packaging), free-range eggs with adequate traceability, menu design to prioritise meat-free options and tailor to seasonal offerings, preference for fresh over frozen fish, prioritise suppliers who deliver goods with reusable packaging - Promote equitable forms of dialogue with farmers/producers	16^(i,ii,iii,xi,xiii,xv,xx,xxi,xxii,xxv,xxviii,xxix,xxxi,xxxii,xxxiii,xxxv)^	E, L, N, S	1–12
	Engage nutrition professionals in school feeding program menu design to ensure local culture, eating traditions, environmental sustainability and agricultural diversity are considered	1^(xx)^	L	1–12
	Improve local food distribution by investing in infrastructure, technology, transportation and planning to connect consumers with producers by including one or more of these strategies: - Establish an online portal for local businesses, community agencies and residents to order fresh and healthy foods at wholesale prices - Establish distribution hubs to allow groups of producers to sell direct - Expand and support Community Supported Agriculture initiatives from urban and fringe farms - Improve public transport options to increase physical access to locally produced food - Enable pop-up fresh produce market stalls at public facilities to promote access to organic, seasonal, locally grown and fairly traded food	10^(v,xvi,xix,xxi,xxiii,xxiv,xxv,xxvii,xxx,xxxvi)^	E, L, N, S	1–4, 7–8
	Support and expand alternative retail options such as farmers’ markets, food co-ops, on-site school programs and mobile grocery stores, prioritising low-income neighbourhoods and peri-urban zones	9^(ii,v,xix,xxi,xxiii,xxv,xxvii,xxviii,xxxii)^	E, L, N	1–4, 7–8
	Engage dominant retail chains as partners to promote healthy and sustainable choices through training of retail staff, point-of-sale messaging, tastings and video messages at cash registers	3^(v,xxv,xxix)^	E, N	1–2, 4
	Revitalise local food businesses involved in food manufacturing, processing, distribution and storage through tax exemptions	1^(xxvii)^	N	4
Food waste (*n* 17)	Design urban agriculture initiatives with composting facilities	1^(iv)^	E	10
	Increase awareness about food waste and build capacity to adopt waste minimisation strategies by funding campaigns (e.g. Love Food Hate Waste) and workshops	6^(v,xi,xvi,xix,xxvi,xxvii)^	E, N	10
	Create best-practice guidelines to support food service businesses to reduce their food waste and save money (e.g. reduce cost of waste collection by improving waste separation processes to increase composting potential, encourage diners to take-away leftover food by providing compostable containers, energy recovery from biogas)	2^(ii,x,vi)^	E	10
	Incentive programs for residents to use household food recycling bins and businesses to create closed-waste cycles (e.g. mushrooms from coffee grounds)	3^(ii,v,xvi)^	E	10
	Develop kitchen and processing facilities onsite at local farms to use farm surpluses in preserves, chutneys, dehydrated and fermented foods to be sold direct to local restaurants	1^(xvi)^	E	3–4, 10
	Integrate composting and wormery facilities within school garden programs	1^(xvi)^	E	10
	Conduct research to establish the extent and contributors of city-wide food waste to inform targeted strategy	1^(xvi)^	E	N/A
	Connect surplus local food with restaurants, catering industry and food rescue/relief services (e.g. Digital marketplace for surplus food)	3^(ii,v,xvi)^	E	2, 4, 10
	Establish a sustainable restaurant award scheme for small-medium businesses to incentivise sustainable practices, with food waste audits as part of the adjudication process	1^(xvi)^	E	1–12
	Provide high-capacity food digester facilities for businesses to dispose of their organic waste	1^(xxvi)^	N	10
	Advocate to federal government to revise ‘best before’ and ‘use-by’ labelling regulations	1^(xxvii)^	N	10
	Mandate residents’ responsibility to separate recyclables, compostable and landfill-bound trash, with compliance audits, including provision of green waste bins by the City	2^(xxvi,xxxiii)^	N	10
	Require suppliers and contractors of public facilities to take responsibility for their waste generation by including responsibility language in City purchasing contracts	1^(xxxiii)^	N	10
	Create a sustainability charter for events on City property to prohibit single-use food service ware and polystyrene use, promote compostable or recyclable food ware, require reusable beverage cups, enable single-use plastic straws only upon request for people with disabilities and medical needs, promote bottle-filling stations, restrict sale of packaged water and prohibit public funds being spent on bottled water	1^(v)^	E	10, 12
	Integrate food waste management into City food safety handling certification requirements	1^(xxvi)^	N	10
	Facilitate domestic composting sites for residents to use communally	1^(v)^	E	10
	Prioritise commercial bulk food stores to minimise food packaging	1^(v)^	E	7, 10

* City: refers to any local government authority, otherwise referred to as a county, municipality, local government area, province, shire, region, council, office.

† Citations: Roman numerals are linked to the summary of each policy (*n* 36) in Supplemental Material S6: Data extraction from policy document.

‡ Regions: E = Europe & Central Asia, N = North America, L = Latin America and Caribbean, S = Sub-Saharan Africa.

§ Targeted diet-related practice^(28)^: (1) Select food grown using sustainable food production practices, valuing Indigenous knowledges; (2) Strengthen local food systems by connecting with primary producers; (3) Eat seasonally, incorporating native and wild-harvested foods; (4) Eat locally available foods; (5) Avoid over-consumption beyond caloric requirement; (6) Consume no more than recommended amounts of animal-derived foods; (7) Limit intake of highly processed, nutrient poor and over-packaged foods; (8) Increase intake of plant-based foods; (9) Eat a wide variety of foods to promote biodiversity; (10) Adopt food waste-minimisation strategies; (11) Preference home-made meals and share with others; (12) Consume safe tap water as preferred drink; and (13) Breastfeed infants where possible.

‖ Public facilities: government-funded services such as kindergartens, early years day-care centres, public schools, seniors’ centres, public hospitals, recreation centres, homeless shelters, correctional facilities.

## Discussion

This review demonstrates the leadership role played by local governments in developing and implementing policy to promote the uptake of healthy and environmentally sustainable diet-related practices. Existing policies have been mapped against the MUFPP Monitoring Framework to characterise and identify gaps in policy action and highlight exemplars, as described within the following key observations.

### A holistic approach is being taken by local governments, with consideration of the broader food supply chain, health and equity

Many of the policies led by local government to facilitate the uptake of healthy and sustainable diet-related practices adopted a holistic approach. That is, policy action was directed across multiple phases of the food supply chain, rather than simply focusing on the consumption phase, and health and equity were considered in the desired outcomes. A core value in effective urban food policies, as identified by Sonnino (2019), is taking this systemic approach to food, where all phases of the food supply chain are considered and food’s ‘multidimensional connections with different social contexts, sectors and other community systems’ is acknowledged^(58)^, p14. The High-Level Panel of Experts on Food Security and Nutrition (2017) also supports this whole-of-system approach, and recommends that action across the food supply chain is critical to influence peoples’ dietary patterns^(59)^. Although beyond the scope of this review, further research is warranted to explore the capacity of local governments to support multi-sectorial governance with monitoring and evaluation to measure the impact of this whole-of-system approach.

This review highlighted a number of comprehensive local government policies, such as Belgium’s *Ghent en Garde* which demonstrated a holistic approach. *Ghent en Garde* included activity across the food supply chain including production (e.g. promotion of fair trade and organic practices, allocation of urban garden plots), distribution (e.g. an agricultural hub with job-ready training and networking), retail (e.g. farmers markets), consumption (e.g. public facilities serving less meat and more plant-foods) and waste (e.g. supporting local businesses to innovate new models such as growing mushrooms from coffee grounds). The *Ghent en Garde* policy aims to improve health by updating tender processes for public facility food services (e.g. schools and hospitals), ensuring that all food served at publicly funded events on Thursdays is vegetarian, and raising citizen awareness through social marketing campaigns to eat less meat, and consume more local, organic and seasonal foods. Ghent also invested in strategies to address equity such as creating social employment through food, in social restaurants, social grocers and in local production and distribution of food.

Bristol’s Good Food Action Plan presents another example of a holistic approach, by linking with neighbouring local governments, transforming unused public land into edible gardens and acknowledging the value of good quality soil and insect pollinators for local food production. Bristol defines good food as ‘not only tasty, healthy affordable, but also produced and distributed in a way that is good for nature, workers, animal welfare and local businesses’. This policy addresses health by supporting schools to achieve the Mayor’s Award for Excellence by including nutrition, health and gardening in the curriculum and connecting emergency food relief services with local producers of fresh, healthy food. Bristol also piloted a campaign, ‘Flexitarian City’, by promoting a flexitarian diet in local restaurants and communities. In considering equity, urban agriculture initiatives allocate garden plots for people seeking asylum and the Kitchen on Prescription program involves prescribing accessible cooking, gardening and nutrition training opportunities for individuals experiencing disadvantage. Local governments are demonstrating a holistic approach to food system transformation by considering equity in their policy actions; this could be strengthened however as not all local governments had considered this.

Local government policy actions were found to target multiple phases of the food supply chain to shift dietary patterns. However, this review yielded the least policy actions for the *food supply and distribution* category of MUFPP’s framework for action. Policy actions from this category included investment in infrastructure, technology and transportation to connect consumers with producers, revitalisation of local food manufacturing, processing, distribution and storage businesses through tax exemptions and expanding alternative retail options to connect consumers with producers. The reason why this category had the least policy options requires further research. It may be that local governments do not consider this area to be part of their mandate, or perhaps a gap in competency and knowledge exists at the local level relating to these activities, requiring funds and resources from national governments to address this gap^(60)^.

### Local government authorities can lead local action towards global planetary health targets, however they require enhanced capacity

It has been argued that local government is critical to achieving the ambitious global and national targets needed for food sustainability^(59)^. Local governments have a unique role in operationalising ‘on the ground’ action to enable nations to achieve ambitious global sustainable development targets^(61,62)^. As the level of government closest to their constituents, they can be more agile and responsive to changing needs and innovation opportunities than national governments, moving more rapidly from the agenda-setting phase to policy implementation^(61,62)^. This review identified that local government authorities played a role of leadership or ownership in policy action for the majority of policies, rather than one of stakeholder representation or consultation. This highlights internal commitment to make urban food systems more sustainable, resilient and equitable, as demonstrated by these cities’ pledges to the MUFPP^(16)^.

A range of policies was identified which have effectively enhanced local government capacity to lead policy action. First, formal structures and networks outside of local governments were shown to facilitate collaborative action and collective capacity and provided opportunities for local government leadership. The MUFPP^(13)^ is one of these, and others include the C40 Good Food Cities Declaration (https://www.c40.org/other/good-food-cities), and regional networks such as Europe’s Edible Cities Network (www.edicitnet.com). Second, opportunities to enhance capacity were shown to exist within local governments themselves. McCartan and Palermo^(63)^ also found that food policy councils led by local government can provide a forum to combine practical and technical expertise across sectors, and increase capacity through partnerships in an environment where funding is often limited. Third, strategies to connect researchers with local governments facilitated the translation of evidence into practice and enhanced knowledge about the relations between food systems (e.g. urban and rural) and between phases of the food supply chain (e.g. supply and distribution). Finally, policies identified in this review engaged citizens and professionals with technical expertise in decision-making. Examples of this included establishing producers’ networks to facilitate urban, peri-urban and rural linkages, and logistics, nutrition and sustainability experts to inform land use, zoning and new building requirements.

### Publicly funded facilities are suitable settings for action

Food procurement policies in schools were identified as a popular approach by local governments to promote healthy and sustainable diet-related practices and induce systemic change across the food supply chain, as has previously been described by Hawkes *et al.* (2015)^(64)^. Publicly funded settings such as schools, hospitals, community centres, prisons and early learning centres are suitable settings for local governments to fulfil their commitment to achieve planetary health targets. These settings serve large and socio-economically varied groups of people and can shape consumers’ behaviour through their regular interaction with the food environment^(59)^. Local governments may possess regulatory and legislative powers to manipulate these food environments, and align government spending for food procurement, supply and promotion with public priorities^(61)^.

Some procurement policies were enforced nationally, yet implemented at the local government level (e.g. Brazil and Italy), and others were developed at the local level in Vienna (Austria), Ghent (Belgium), Copenhagen (Denmark), London (United Kingdom), New York City and San Francisco (United States of America). Policy actions included in this review comprise: use of tender processes and legislative mechanisms to promote organic, local, fair-trade food production practices; eco-friendly food packaging and transport; and, school menus with less processed and animal-derived foods and more plant-based and nutritious foods. Comment on the effectiveness of these policies is beyond the scope of this review, however, Goncalves *et al*. (2015) concluded that Brazil’s *Family Farming* program which uses legislation to enforce 30 % of Brazilian government funding is used to buy food directly from local family farms, increased the nutritional quality of school menus^(46)^. These examples demonstrate that although focused on one setting, food procurement policies can trigger action across phases of the food supply chain and facilitate a number of healthy and sustainable diet-related practices.

### Progress is needed to promote the double-win (health & environment) of some diet-related practices

While all 13 healthy and sustainable diet-related practices are important to achieve food system transformation, particularly in middle and high-income contexts, some have greater potential than others^(3,4)^. According to Springmann (2020) practices with the greatest potential to improve health and sustainability are those which limit animal-derived foods, in particular beef and dairy, increase wholegrain and plant-based foods and avoid over-consumption^(65)^. This discussion will therefore describe how local governments are promoting these 3 desired practices in particular, and identify some of the trade-offs that exist as well as opportunities to achieve double-wins for human and planetary health.

First, actions to limit the intake of animal-derived foods were identified in less than a third of policies. Eating less animal-derived foods has been demonstrated to improve health, by lowering mortality risk from CVD and some cancers, and reduce environmental degradation, by reducing greenhouse gas contribution, water usage and biodiversity loss^(5)^. This review identified that local governments are promoting less animal-derived foods by updating school feeding guidelines, incentivising procurement practices in commercial and public food service facilities and investing in social marketing campaigns. Policy documents describe the environmental impact of eating less meat as a more dominant message than the health benefits thereof.

In considering approaches to increase wholegrain and plant-based food intake, actions targeting this practice were identified in nearly three quarters of the policies included in this review. It is important to note that the health and environmental benefits rely on the adoption of sustainable agricultural practices and various trade-offs exist. For example, while organic farming is more environmentally sustainable, there are consequences for food security because yields may be lower and produce is often more expensive to purchase. This review identified that local governments are promoting plant-based food consumption by investing in urban agriculture, redirecting rescued fresh produce to emergency food relief services and connecting producers with consumers through alternative retail avenues such as farmers markets. Local governments often framed these policy actions as achieving social and economic benefits, acknowledging that poor fruit and vegetable intake is disproportionately experienced by people experiencing disadvantage, however they were less likely to describe the environmental benefits^(66)^.

The third diet-related practice to discuss in relation to win-wins is the avoidance of over-consumption of food beyond biological requirements. This practice was promoted by one-third of identified policies, however these primarily focused on the health benefits of reducing overweight and obesity. The environmental benefits of avoiding overconsumption of food are being overlooked, despite convincing evidence that this diet-related practice will reduce deforestation, biodiversity loss, ocean acidification, air, water and soil pollution which result from producing food not deemed essential to nourish life^(66,67,68)^. For example, New York City’s Food Standards (2011) policy was primarily intended to reduce diet-related disease by reducing over-consumption, but included sustainability considerations in its supporting documentation.

There is an increased urgency being placed on policy-makers to consider both health and ecological implications of the food system and dietary recommendations^(1,4,65)^. However, this win-win scenario is complicated by trade-offs which challenge local governments to simultaneously promote health, social justice and environmental sustainability. For example, in promoting organic food production to benefit human health and the environment, governments must consider the lower yields, higher land requirements and increased cost to consumers. To address these complex trade-offs, trans-disciplinary evidence from health, nutrition, environment, the social sciences and beyond must inform policy action to support best-practice across the entire food supply chain.

As described above, this review presents 4 key observations to inform future policy action: (1) a holistic approach is being taken by local governments, with consideration of the broader food system, health and equity; (2) local government authorities can lead local action towards global planetary health targets however require enhanced capacity; (3) publicly funded facilities are suitable settings for holistic policy to be implemented; and (4) progress is needed to promote the double win (health and environment) of recommended diet-related practices.

### Limitations and implications for practice

The scope of this review was refined to include an exploration of policies that were cited within literature sourced from scientific databases. This presents some limitations: (i) policies from low-middle income countries have not been adequately represented most likely due to variance in the degree to which governments can engage academic resources to publish outcomes of policy action; and (ii) current best practice examples may have been missed simply because they have not yet been published in the scientific literature. In refining the scope to MUFPP signatory cities, this review does not include the many progressive policies that exist globally that are independent to the MUFPP movement. To identify relevant policy actions, this review referred to 13 diet-related practices identified by the authors in a previous study^(28)^ however this is just one publication within a large, and rapidly expanding body of evidence to describe healthy and sustainable diets.

Further research is required to explore the intricacies of local government policymaking, such as how policies and their goals are prioritised, why some policy actions receive investment over others and how success and failure can effectively be defined^(18)^. Likewise, research into the effectiveness and cost-efficiencies of the policies identified in this review, across a range of geographic contexts, is recommended in order to support local governments to develop and implement policies that have the best chance of achieving food system transformation to improve human health and environmental sustainability.

## Conclusions

This scoping review identified policies that promote healthy and environmentally sustainable diet-related practices. Local governments are considering both health and equity in their choice of policy actions to shift population-level diets; engaging a diverse range of stakeholders in the policy-making process; creating governance structures which connect with neighbouring areas and key stakeholders; and using public procurement policy actions as a common strategy to address wide-ranging challenges across the food supply chain.

Local government authorities, at the interface between citizens and state and national decision-makers, have a critical role to play in shifting population-level food consumption. This review showcases policies from cities committed to the MUFPP, to inform local government authorities seeking a more comprehensive policy response. By localising global sustainable development targets, food policy can promote healthy and sustainable diets to drive the food system transformation required to sustain human lives into the future, within planetary boundaries.

## Supplementary material

For supplementary material accompanying this paper visit https://doi.org/10.1017/S1368980021004432. click here to view supplementary material
