# Supplementary material for: Local urban government policies to facilitate healthy and environmentally sustainable diet-related practices: a scoping review
Source: Public Health Nutr. 2021 Oct 25;25(2):471–87. doi: 10.1017/S1368980021004432 (PMC8883777; doi:10.1017/S1368980021004432)
Supplement: Supplementary file 1 [file S1368980021004432sup.zip › S1368980021004432sup001.docx]

**Supplementary Materials**

Table of Contents

[Supplementary Material S1: Commonly cited healthy and environmentally sustainable diet-related practices 2](#_Toc83135554)

[Supplementary Material S2: Description of each healthy and sustainable diet-related practice 3](#_Toc83135555)

[Supplementary Material S3: Search terms for peer-reviewed publication search 7](#_Toc83135556)

[Supplementary Material S4: Summary characteristics of included studies 9](#_Toc83135557)

[Supplementary Material S5: Data extraction from included studies 10](#_Toc83135558)

[Supplementary Material S6: Summary of data extracted from the policy documents cited in included studies 17](#_Toc83135559)

[Reference List of Included Studies 62](#_Toc83135560)

# Supplementary Material S1: Commonly cited healthy and environmentally sustainable diet-related practices

| ***Diet-related practice cited in relevant United Nations’ publications*** | ***Sustainable Healthy Diets*** | ***Eat Lancet Commission*** | ***A Healthy Diet Sustainably Produced*** | ***Sustainable Diets for Healthy People & Planet*** | ***Nutrition and Food Systems*** | ***UN Decade of Action on Nutrition*** | ***Plates Pyramids***  ***Planets*** | **Sustainable Diets & Biodiversity** |
| --- | --- | --- | --- | --- | --- | --- | --- | --- |
|  | *FAO & WHO (2019) ^[[1]](#footnote-1)^* | *Willett et al (2019) ^[[2]](#footnote-2)^* | *WHO (2018) ^[[3]](#footnote-3)^* | *UNSCN (2017) ^[[4]](#footnote-4)^* | *HLPE (2017) ^[[5]](#footnote-5)^* | *UN (2016) ^[[6]](#footnote-6)^* | *FAO (2016) ^[[7]](#footnote-7)^* | *FAO (2012) ^[[8]](#footnote-8)^* |
| Select food grown using sustainable food production practices, valuing and respecting Indigenous knowledges | 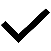 | 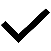 | 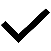 | 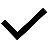 i | 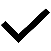 | 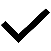 | 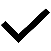 |  |
| Strengthen local food systems by connecting with primary producers | 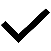 |  | 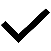 |  | 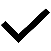 |  | 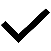 |  |
| Eat seasonally, incorporating native and wild-harvested foods | 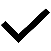 | 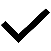 |  | 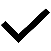 | 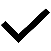^ii^ |  | 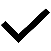 | 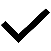 |
| Eat locally available foods | 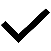 | 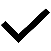 | 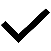 | 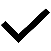 | 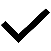 |  | 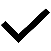 | 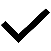 |
| Avoid over-consumption beyond caloric requirement | 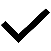 | 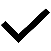 | 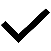 | 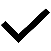 |  | 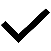 | 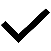 |  |
| Consume no more than recommended amounts of animal-derived foods | 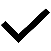 | 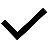iii | 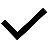iii | 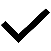 |  |  | 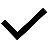iv |  |
| Limit intake of highly processed, nutrient poor and over-packaged foods | 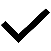 | 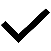 | 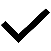 | 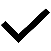 | 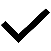 | 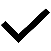 | 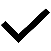 |  |
| Increase intake of plant-based foods | 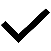 | 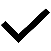 | 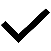 | 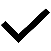 | 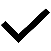 | 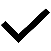 | 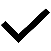 |  |
| Eat a wide variety of foods to promote biodiversity | 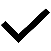 | 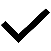 | 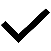 |  | 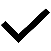 | 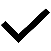 | 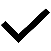 | 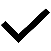 |
| Adopt food waste-minimisation strategies | 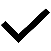 | 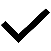 |  | 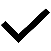 |  | 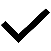 | 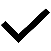 |  |
| Preference home-made meals and share with others |  |  | 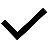 |  | 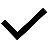 |  | *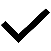* |  |
| Consume safe tap water as preferred drink | 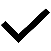 |  | *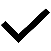* |  |  | *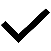* | *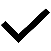* |  |
| Breastfeed infants where possible | 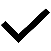 | 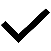 | 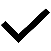 |  | 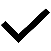 | 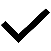 | 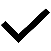 |  |
| *Publications focused on specific details of each practice in some cases: ^i^ Affordability, education, seafood. ^ii^ Traditional foods. ^iii^ Including dairy. ^iv^ All animal parts consumed. ^v^ Home prepared. ^vi^ Cooking.* | | | | | | | | |

# Supplementary Material S2: Description of each healthy and sustainable diet-related practice

| ***Diet-related practice*** | ***Evidence to justify each practice*** |
| --- | --- |
| Select food grown using sustainable food production practices, valuing and respecting Indigenous knowledges | Food production is one of the main drivers of climate change, land and water use, biodiversity loss and disturbance to nitrogen and phosphorus cycles^1^. The EAT-Lancet Commission (2019) has demonstrated that an integrated agenda, considering both food production practices and population diets, is required to preserve human health within planetary boundaries ^1,2^.  Sustainable food production can be achieved by shifting priorities away from producing large quantities of food to meet the global populations’ calorie needs, towards food production that uses innovative practices to intensify the production of healthy and high-quality food, embraces holistic governance of land and waterways, and reduces food losses during production^1^. Examples of sustainable food production approaches include conservation agriculture, agroecology, diversified farming systems, organic farming, precision agriculture, sustainable and ecological intensification. Specific farming and fishing practices include using pest control, pollination, water regulation and nutrient cycling to achieve productivity and resilience while reducing harmful environmental effects^2^.  Preserving biodiversity including crops, livestock, forest and aquatic derived foods is essential to adequately nourish a growing global population, and can be achieved through effective governance to avoid unsustainable practices such as over-fishing and over-hunting, which helps ensure food and nutrition security into the future^3^. These strategic priorities have the potential to reduce existing global yield gaps, manage nitrogen distribution and phosphorus fertiliser use, optimise fertiliser and water use, change dominant agricultural practices to reduce green-house gas emissions and fundamentally shift food production priorities to prioritise planetary and human health outcomes^1^.  Existing Indigenous food systems have been diversifying diets with nutritious foods while simultaneously preserving biodiversity and environmental sustainability for hundreds and in many cases thousands of years^4^. Indigenous food systems can offer best-practice evidence to inform approaches to conserve resources, nourish human health and strengthen ecosystems for climate change resilience^4^. Inter-generational transmission of traditional knowledge in and beyond Indigenous communities is critical to influence high-level policy and practice, as is being facilitated on a global scale by the FAO (2018) ^4^. This is not only crucial to achieve food system sustainability for the population at large, but also to preserve Indigenous food systems and associated knowledges for generations to come^5^. |
| Strengthen local food systems by connecting with primary producers | Consumers’ consideration of sustainability is affected by the value they place on food and their knowledge of the local food system. Connecting consumers with producers not only reduces the physical distance of the food supply chain, it also creates a platform for consumers to socially and emotionally engage with primary producers, adding value and additional meaning to their food. Respect for “local culture, culinary practices, knowledge and consumption patterns, and value the way food is sourced, produced and consumed” are the hallmarks of healthy and sustainable diet-related practice^3, p11^. |
| ***Diet-related practice*** | ***Evidence to justify each practice*** |
| Eat seasonally, incorporating native and wild-harvested foods | Reverting to more traditional food systems, which “rely on minimally processed seasonal foods, collected or produced for self-consumption or sold mainly through informal markets” and sustained life on earth for thousands of years, have both desirable health and environmental outcomes^6, p2^. Eating seasonally and relying on locally available foods also reduces the need for refrigeration, storage and transportation of food and therefore minimises GHG emissions and the resulting environmental impact. |
| Eat locally available foods | According to the WHO, policy to promote a healthy and sustainable diet should “connect smallholder farmers and local producers with consumers to promote easy access to local markets, shorten production chains and use local produce in school feeding programmes, hospitals and other institutions.” ^7, p6^ Improving the connection between consumer demand and food supply (across rural, per-urban, urban settings) will have many desirable outcomes including greater diversity and nutritional value of available fresh foods, shorter supply chains and increased support for local economies (markets, e-commerce, community supported agriculture)^6^. Consumers are encouraged to adapt their diet to preference locally available foods, knowing that to achieve the lowest environmental impact, foods need to be produced under optimal climatic conditions for each specific food therefore not all foods can be grown or raised locally at all times of the year. |
| Avoid over-consumption beyond caloric requirement | Food that is consumed beyond an individual’s nutritional requirement “represents an avoidable environmental burden in the form of GHG emissions, use of natural resources and pressure on biodiversity” ^8, p1159^. Burlingame from the FAO describes this as *metabolic food waste*, whereby the consumption of food in excess of nutritional requirements manifests as overweight and obesity^9^. According to EAT, if current consumption patterns continue, by 2050 our global population’s food-related GHG emissions will have exceeded planetary boundaries by 263%, with the majority coming from overconsumption of food which should be considered ‘limited’ or ‘optional’ to achieve human health^2,^ ^p29^. A further consequence of over-consumption of these foods is the natural resources required to support the healthcare sector in the management and treatment of obesity and other diet-related diseases. |
| Consume no more than recommended amounts of animal-derived foods | Current dietary consumption patterns (in G20 countries at a population level) of animal-derived foods is far exceeding the recommended amounts required to nourish the population within planetary boundaries, as defined within the Planetary Health Diet^2^. Current red meat consumption is over 500% in excess of the recommended maximum 28 g/day^2^, increasing risk for some diet-related cancers. Egg consumption is over 200% of the recommended 13 g/day^2^. For fish, poultry and dairy, the average consumption across all G20 countries is at or below the recommended amounts, however some countries have very little consumption of these foods due to limited resources and cultural/religious determinants while other countries are far-exceeding the recommended values^2^. When selecting animal-derived foods in amounts consistent with Dietary Guidelines, the prioritisation of beef, seafood and poultry raised using sustainable farming practices is essential. Some examples of such considerations in livestock production include effective manure management, feed conversion, and the use of feed additives that reduce enteric fermentation^2^. |

| ***Diet-related practice*** | ***Evidence to justify each practice*** |
| --- | --- |
| Limit intake of highly processed, nutrient poor and over-packaged foods | Processed foods, especially those in the ‘ultra-processed food’ (UPF) category such as savoury snacks, processed meats, sugar-sweetened beverages, confectionery, frozen desserts, breakfast cereals and dairy products are high in trans fats, salt and added sugar and offer minimal nutritional value^10,11^. UPFs are defined as “formulations of ingredients, mostly of exclusive industrial use, that result from a series of industrial processes (hence ‘ultra-processed’), many requiring sophisticated equipment and technology” ^10^. The manufacturing and packaging of these foods use greater amounts of environmental resources than less processed foods, while also contributing to food-related waste^12^. Consumption of ultra-processed foods is increasing modestly in high-income countries, and more rapidly in upper-middle and lower-middle-income countries^11-13^. |
| ­­­­­­­­­­  Increase intake of plant-based foods | Current dietary consumption patterns (in G20 countries at a population level) of plant-based foods are not meeting the recommended amounts required to nourish the population within planetary boundaries, as defined within the Planetary Health Diet^2^. Nut consumption is the greatest deviation from the recommended 50 g/day. Legume consumption is less than 30% of the recommended 75 g/day^2^. Fruit intake is below the recommended 200 g/day in all G20 countries except Brazil, Turkey, Italy, Canada and France^2^. Vegetable consumption is only meeting the recommended 300 g/day in Turkey, Italy, USA, South Korea and China of all the G20 countries^2^. Plant-based foods grown using water sensitive farming practices should be preferentially selected for sustainability and nutrition gains. |
| Eat a wide variety of foods to promote biodiversity | At a consumer level, eating a variety of food is essential to meet nutritional requirements, while at a population level, the promotion of dietary variety is vital to “preserve biodiversity, including that of crops, livestock, forest-derived foods and aquatic genetic resources”, and minimise risk of depleting natural resources^1, p10^. |
| Adopt food waste-minimisation strategies | The EAT-Lancet Commission identified that a reduction in food loss (during food production) and food waste (consumption end of supply chain) is essential and reinforced the SDGs target to halve food loss and waste by 2030^1^. Food waste minimisation strategies rely on effective meal planning to avoid over-purchasing or sourcing of food, energy-efficient food storage facilities and equipment to lengthen shelf life, reduced reliance on processed foods with inorganic packaging, adequate cooking skills to utilise leftover ingredients, access to composting or worm farming to dispose of unavoidable organic food waste and adequate knowledge to determine items suitable for non-landfill destinations. |
| Preference home-made meals and share with others | Consumption of food with others is conducive to the enjoyment of food, and “encourages eating regularly, attentively, and in appropriate environments” where over-consumption of food is less likely^15,p1^. Home-made meals are more likely to include fresh, healthy ingredients^13, 14^. Home-prepared meals, especially when shared with others, have also been noted to contribute to the conservation of natural resources such as water, energy and gas required to produce the meal^16^. |

| ***Diet-related practice*** | ***Evidence to justify each practice*** |
| --- | --- |
| Consume safe tap water as preferred drink | Where safe and clean drinking water is accessible, there is no requirement for packaged drinks to meet daily fluid requirements^3^. The environmental impact of water consumption is minimal when the infrastructure and practices used to process water from its original source to a safe and clean fluid for human consumption consider all the dimensions of sustainability, and when water is consumed from reusable vessels. |
| Breastfeed infants where possible | Early initiation of breastfeeding, idealy exclusively until six months of age, then continued until two years and beyond with appropriate complementary feeding is recommended^17^. Breastfeeding offers a source of nutrition, together with many social and emotional benefits, without any environmental impact. Breastmilk is a “natural, renewable food that is environmentally safe and produced and delivered to the consumer without pollution, packaging or waste”^18^. The alternative, milk formula, generates greenhouse gas emissions and it’s estimated that 1kg of breastmilk substitute powder requires more then 4000L of water^18^. If breastfeeding is able to occur naturally, there is no requirement for packaged or processed materials to nourish a human for the first six months of their life. World Breastfeeding Week 2020 was dedicated to supporting breastfeeding to promote a healthier planet^18^. |
| FAO – Food and Agricultural Organisation, GHG – Greenhouse Gas, WHO – World Health Organisation, G20 Countries – An international forum for global economic cooperation of 19 countries and the European Union (EU)^2^, SDGs – Sustainable Development Goals (United Nations’ 2030 Agenda), | |
| Willett, W. et al. Food in the Anthropocene: the EAT–Lancet Commission on healthy diets from sustainable food systems. Lancet, 2019. 393(10170): p. 447-492.   1. EAT. Diets for a Better Future: Rebooting and Reimagining Healthy and Sustainable Food Systems in the G20. 2020. EAT-Lancet Commission on Food, Planet, Health.   Food and Agriculture Organisation and World Health Organisation. Sustainable healthy diets - Guiding principles. 2019. Rome.  United Nations. High-Level Expert Seminar on Indigenous Food Systems: Building on traditional knowledge to achieve zero hunger. FAO, FILAC and UN Permanent Forum on Indigenous Issues. 2018. FAO Head Quarters, Rome.   1. Turner, N., Plotkin, M. & Kuhnlein, H. Global environmental challenges to the integrity of Indigenous Peoples’ food systems. FAO of the United Nations. 2013. FAO Headquarters, Rome 2. High Level Panel of Experts. Nutrition and food systems. A report by the High-Level Panel of Experts on Food Security and Nutrition of the Committee on World Food Security. 2017. Rome. 3. World Health Organisation, A healthy diet sustainably produced - information sheet. 2018. 4. Friel, S., Barosh, L. & Lawrence, M. Towards healthy and sustainable food consumption: an Australian case study. 2014. Public Health Nutrition, 17, pp1156-1166. 5. Institute of Medicine. Sustainable diets: Food for healthy people and a healthy planet: Workshop summary. 2014. Washington DC, The National Academies Press 6. Monteiro C.A., Moubarac J.C., Cannon G. et al. Ultra-processed products are becoming dominant in the global food system. 2013. Obesity Reviews, 14, pp. 21–28 7. Development Initiatives Poverty Research Ltd. 2020 Global Nutrition Report: Action on equity to end malnutrition. 2020. Bristol, UK. 8. Fardet, A. & Rock, E. Ultra-Processed Foods and Food System Sustainability: What are the Links? 2020. Sustainability, 12, 6280. 9. Baker, P. et al. Ultra-processed foods and the nutrition transition: Global, regional and national trends, food system transformations and political economy drivers. 2020. Obesity Reviews. pp1-22. 10. Wolfson, J., Leung, C. & Richardson, C. More frequent cooking at home is associated with higher Healthy Eating Index-2015 score. 2020. Public health Nutrition, 23 (13), pp 2384-2394. 11. Ministry of Health of Brazil. Dietary Guidelines for the Brazilian Population. 2015. Secretariat of Health Care, Brazil. 12. Fischer, C. and Garnett, T. Plates, pyramids and planets. Developments in national healthy and sustainable dietary guidelines: a state of play assessment. 2016. FAO and the Food Climate Research Network at The University of Oxford. 13. World Health Organisation. Infant and young child feeding – Fact Sheet. 2020. <https://www.who.int/news-room/fact-sheets/detail/infant-and-young-child-feeding> 14. World Alliance for Breastfeeding Action (WABA). World Breastfeeding Week 2020: Support breastfeeding for a healthier planet. 2020. [www.worldbreastfeedingweek.org](http://www.worldbreastfeedingweek.org) | |

# Supplementary Material S3: Search terms for peer-reviewed publication search

***Scopus*** search (with title, abstract and keyword search):

| *1* | *Population - local government* | (local or municipal* or county or counties or shire* or provin* or regional or city or town* or urban or metropolitan) w/5 (council or authorit* or govern* or board* or service* or office*) |
| --- | --- | --- |
| *2* | *Intervention - policy* | Policy or policies or act or strateg* or plan or plans or scheme or initiative* or intervention* or program* or action* or law or legislat* or guideline* or regulat* |
| *3* | *Outcome - food* | Food* or diet* |
| *4* | *Outcome - environmental sustainability* | Green or sustainab* or greenhouse gas or (carbon w/4 emissions) or GHG or climate or unsustainab* or enviro* or ecolog* or health* or eco-friendly |

***Medline*** search (with title, abstract and keyword search):

| *1* | *Population - local government* | (local or municipal* or county or counties or shire* or provin* or regional or city or town* or urban or metropolitan) ADJ5 (council or authorit* or govern* or board* or service* or office*) |
| --- | --- | --- |
| *2* | *Intervention - policy* | Policy or policies or act or strateg* or plan or plans or scheme or initiative* or intervention* or program* or action* or law or legislat* or guideline* or regulat* |
| *3* | *Outcome - food* | Food* or diet* |
| *4* | *Outcome - environmental sustainability* | Green or sustainab* or greenhouse gas or (carbon ADJ4 emissions) or GHG or climate or unsustainab* or enviro* or ecolog* or health* or eco-friendly |

***CINAHL Plus*** search (with title, abstract and keyword search):

| *1* | *Population - local government* | (local or municipal* or county or counties or shire* or provin* or regional or city or town* or urban or metropolitan) W5 (council or authorit* or govern* or board* or service* or office*) |
| --- | --- | --- |
| *2* | *Intervention - policy* | Policy or policies or act or strateg* or plan or plans or scheme or initiative* or intervention* or program* or action* or law or legislat* or guideline* or regulat* |
| *3* | *Outcome - food* | Food* or diet* |
| *4* | *Outcome - environmental sustainability* | Green or sustainab* or greenhouse gas or (carbon W4 emissions) or GHG or climate or unsustainab* or enviro* or ecolog* or health* or eco-friendly |

***Global Health*** search (with title, abstract and keyword search):

| *1* | *Population - local government* | (local or municipal* or county or counties or shire* or provin* or regional or city or town* or urban or metropolitan) AND (council or authorit* or govern* or board* or service* or office*) |
| --- | --- | --- |
| *2* | *Intervention - policy* | Policy or policies or act or strateg* or plan or plans or scheme or initiative* or intervention* or program* or action* or law or legislat* or guideline* or regulat* |
| *3* | *Outcome - food* | Food* or diet* |
| *4* | *Outcome - environmental sustainability* | Green or sustainab* or greenhouse gas or (carbon AND emissions) or GHG or climate or unsustainab* or enviro* or ecolog* or health* or eco-friendly |

***Proquest – Agricultural & Environmental Science Collection*** search (with title, abstract and keyword search):

| *1* | *Population - local government* | (local or municipal* or county or counties or shire* or provin* or regional or city or town* or urban or metropolitan) NEAR/5 (council or authorit* or govern* or board* or service* or office*) |
| --- | --- | --- |
| *2* | *Intervention - policy* | Policy or policies or act or strateg* or plan or plans or scheme or initiative* or intervention* or program* or action* or law or legislat* or guideline* or regulat* |
| *3* | *Outcome - food* | Food* or diet* |
| *4* | *Outcome - environmental sustainability* | Green or sustainab* or greenhouse gas or (carbon NEAR/4 emissions) or GHG or climate or unsustainab* or enviro* or ecolog* or health* or eco-friendly |

# Supplementary Material S4: Summary characteristics of included studies

| *Characteristic* | *Number of studies*  *(n=27) ^*^* |
| --- | --- |
| Study Design  Qualitative  Quantitative  Mixed methods  Unclear | 21  0  4  2 |
| Research methods  Interviews  Surveys  Focus groups  Written reflections and observation notes  Content/document/policy analysis  Reviews  Case studies | 11  4  2  2  16  4  10 |
| Targeted Population  Government stakeholders not at local government level  Local Government stakeholders (leaders, city planners)  Funders  Individuals experiencing disadvantage (socio-economic, disability, elderly, public housing residents, ‘priority’ neighbourhoods, etc)  Farmers (primary producers of food, landowners)  Food procurement managers  School communities  Researchers (including local-level monitoring and evaluation)  Citizens | 9  21  2  1  3  1  2  1  3 |
| * Citations are provided in the Reference List of Included Studies (final page of these Supplementary Materials) | |

# Supplementary Material S5: Data extraction from included studies

| **Citation *** | **Year** | **Authors** | **Title** | **Reference for publication source** | **Study Aim** | **Study Design**  **(Qual, Quant or Mixed)** | **Targeted population** | **Cited Urban Policies** | **Signatory City (Country)** | **Key Findings** |
| --- | --- | --- | --- | --- | --- | --- | --- | --- | --- | --- |
| 1 | 2018 | Timpanaro, Foti, Scuderi, Toscano & Romano | Urban agriculture as a tool for sustainable social recovery of metropolitan slum area in Italy: case Catania | Acta Hortic. DOI 10.17660/ActaHortic.2018.1215.58 Proceedings International Symposium on Greener Cities for More Efficient Ecosystem Services in a Climate Changing World | Evaluate the benefits (social, economic and environmental) of an urban vegetable gardening project of Catania, called 'Orti e arte' (vegetable gardens and arts). Results will inform social and environmental upgrading of urban mosaics, according to an 'agro-urban planning' approach. | Qual and quant - Likert-type rating scale to quantify and explore different effects. Survey conducted face-to-face with technicians of the Catania City Council, stakeholders and residents of the area | Residents of Librino (70,000 inhabitants), a 'degraded' district in the City of Catania | Orti e arte' is part of the 'Citizens and Regions of the EU for a Sustainable Food Policy' initiative | Catania (Italy) | Social benefits: Secure spaces in the city, exchange report, food security, know-how exchange, awareness of the food value, social inclusion, best quality of life. Economic benefits: Reproduction of landraces, short supply chain, reduce food budget, maintenance of public spaces, project of formation. Environmental benefits: Protection of bio-diversity, healthy safety, landscape improvement, maintenance of the rural culture, recycle organic matrixes. |
| 2 | 2017 | Scwartzman, Rodriguez Mora, Bogus & Villar | Background and elements of the linkage between the Brazilian school feeding program and family farming | Reports in Public Health. DOI: 10.1590/0102-311X00099816 | Analyse the process of drafting the legislation about family farming procurement, through a historical contextualisation, and present a graphical representation of the program's objectives, target population, actions implemented and expected results. | Qualitative. Interviews with people involved in drafting the legislation. Data included: (i) information collected at the interviews; (ii) theoretical and legal framework (documents and legislation on the issues); (iii) the main researcher´s experience and knowledge. The information was then systematized in a diagram. | This study: policy-makers. The policy: students of basic education in public, philanthropic, community and youth and adult schools and family farmers. | National School Feeding Program of Brazil | Araraquara, Belo Horizonte, Curitiba, Porto Alegre, Praia, Rio de Janeiro, Sao Paulo (Brazil) | School feeding emerged in Brazil in the 1940's, then demand for family farming procurement began late 1980's from social movements and family farmers. Local procurement for the school feeding program was happening via an ad-hoc basis, usually depending on municipality-level factors. Family farming legislation aims to provide diversified and healthy school meals with low processed foods, promote short-chain (local) markets, promote local farm production. Programmatic elements were visualised including activities, outputs, direct and indirect effects. |
| 3 | 2019 | Saumel, Reddy & Wachtel | Edible City Solutions - One Step Further to Foster Social Resilience through Enhanced Socio-Cultural Ecosystem Services in Cities | Sustainability 2019, 11, 972, doi:10.3390/su11040972 | (i) Introduce conceptual framework of the Edible City Solutions (ECS), (ii) Review scientific evidence on ECS benefits, (iii) Explore benefits (case studies of Rotterdam, Andernach, Oslow, Heidelberg & Havana), (iv) identify and discuss strategies for implementation. | Qualitative. Literature Review (with some elements of systematic methods) and Case Study research. Case studies involved document analysis, a questionnaire with main contact people within the case study cities and a SWOT analysis. | Cities that are developing strategies related to Edible City Solutions as part of a world-wide growing network of Edible Cities. | Edible City Solutions, Edible Cities | Rotterdam (Netherlands) | ECS demonstrate a high potential for a participatory development of social cohesion – co-planning, co-design and co-implementation are essential. However, a lack of mainstreaming knowledge on ECS technologies, experiences and provided ecosystem services exists. Challenges include; short term actions within election periods, sectoral city administrations and lack of integration into urban planning process. |
| 4 | 2018 | Sanye-Mengual, Kahane, Gianquinto & Geoffriau | Evaluating the current state of rooftop agriculture in Western Europe: categories and implementation constraints | Acta Hortic. 1214. DOI 10.17660/ActaHortic.2018.1215.60 | This study aims to evaluate the current state of rooftop agriculture (RA) in Western Europe, focussing on the different categories of initiatives and the implementation constraints. | Qualitative. A systematic online search to identify current RA projects in Europe which were then categorised (methodology for creating typology is unclear). Case studies are descriptive in nature, methods for collecting information is not stated. | Policy-makers, government stakeholders, funders, citizens | Community rooftop garden of Via Gandusio (Bologna, Italy) and Topager (Paris, France) | Bologna (Italy), Paris (France) | Four categories of types of rooftop gardens were identified: (i) Garden (ii) Farm (iii) Engineering (iv) Landscape. Key implementation constraints include building/engineering competency, legal aspects (resistance of building materials, security access to roofs, waste management, etc), neighbourhood acceptance. |
| 5 | 2018 | Mulligan, Archbold, Baker, Elton & Cole | Toronto municipal staff and policy-makers' views on urban agriculture and health: A qualitative study | Journal of Agriculture, Food Systems and Community Development. 8(Suppl. 2), 133-156. doi:10.5304/jafscd.2018.08B.001 | Identify tensions in the context of food policy developments in Toronto by seeking the views of municipal staff and policy-makers. | Qualitative. Semi-structured key informant interviews (n = 18) | Key municipal staff and policy decision-makers in urban agriculture (working in government and community agencies at municipal and provincial levels) and farmers, landowners and funders. | Toronto Food Policy Council (1990), Toronto Food Charter (2001), GrowTO: An Urban Agriculture Action Plan for Toronto (2012), Toronto Food Strategy (2015-2019) | Toronto (Canada) | Interviewees identified 5 categories of interest in relation to urban agriculture and health; (i) Economic development and employment, (ii) Equity and Health, (iii) Risk, (iv) Land use and production, and (v) Partnerships and policies. Key informants advised that better approaches to the potential risks involved in urban agriculture are required. The results informed the agenda for urban agriculture at the intersections of population health, environmental sustainability, and urban governance. |
| 6 | 2018 | Lazzarini | The role of planning in shaping better urban-rural relationships in Bristol City Region | Land Use Policy, 71 (2018), 311-319. doi:10/1016.jlandusepol.2017.12.005 | Analyse the planning policies at local and city-regional level dealing with and affecting rural areas in the context of Bristol City Region. | Survey | Local government stakeholders | Bristol's Good Food Action Plan (2015-2018) | Bristol (UK) | Results demonstrate that most of the failings in supporting the development of rural areas relate to the rigidity of planning policies and to the resistant attitude towards innovation. The paper argues that a more proactive and integrated approach for planning is needed to re-build stronger agri-food relations and to achieve a more sustainable land use management at city regional level. |
| 7 | 2015 | Tsui, Wurwarg, Poppendieck, Deutsch, Freudenberg | Institutional food as a lever for improving health in cities: the case of New York City | Public Health, 129 (2015), 303-309. doi:10.1016.j.puhe.2014..12.006 | To describe and examine the factors that most facilitate and impede the provision of healthy foods in a complex institutional food system. | Document review and interviews | Relevant City government staff. | New York City Food Standards | New York (USA) | Making changes to institutional food systems that will meaningfully influence public health requires a detailed understanding of the diverse systems supporting and shaping public food provision. Ultimately, the cases in this study demonstrate that agency staff would like to provide healthier foods, however often feel limited by competing objectives of affordability and consumer preference. Their ability to address these is influenced by external (nutritional regulations), and internal forces (agency's structure and staff motivation). |
| 8 | 2016 | Sonnino | The new geography of food security: exploring the potential of urban food strategies | The geographical Journal, Vol 182, No 2, pp 190-200. doi:10.1111/geoj.12129 | (i) What type of ‘foodscape’ do these documents envision, and why? (ii) Does the rescaling of food governance coincide with the emergence of a new localistic approach to food security? (iii) What type of priorities and concrete measures do city governments identify to deal with the new geography of food security? | Document analysis of 15 urban food strategies from Canada, the USA and the UK | Urban food strategy documents | Food Strategies for Toronto, Vancouver, New York City, Chicago, San Francisco, Bristol, London, Brighton and Hove | Toronto & Vancouver (Canada), New York City, Chicago & San Francisco (USA), Bristol, London, Brighton and Hove (UK) | Urban food strategies are highlighting the vital role of physical infrastructure and policy integration to improve food security and sustainability. An emerging agenda exists that is striving to improve food in relation to public health, community development, environmental integrity and sustainable land use – these are values that are amplified by complex dynamics within the new geography of food security. |

| 9 | 2016 | Smith, Andersson, Gourlay, Karner, Egberg Mikkelsen, Sonnino, Barling | Balancing competing policy demands: the case of sustainable public sector food procurement | Journal of Cleaner Production. 112. 249-256. http://dx.doi.org/10.1016/j.jclepro.2015.07.065 | (i) Clarify the difference between green and sustainable public sector food procurement, (ii) demonstrate the reality of devising and implementing innovative approaches to sustainable public sector food procurement and (iii) identify the effects of cultural and political framings. | Case study (n=5) research involving knowledge brokerage activities of the Communities of Practice (CoP). Data was obtained from the activities of the public sector procurement CoP. | European cities where innovative public procurement policies and practices have been successfully implemented. | | Sustainable Food Procurement for Schools in Rome, Organic Program (Copenhagen House of Food), OkoKauf Wien ('EcoBuy') in Vienna | Rome (Italy), Copenhagen (Denmark), Vienna (Austria) | These case studies demonstrate the reality of implementing sustainable public sector food procurement. Key drivers: enactment of national-level legislation (and its local-level interpretation, political will & leadership, infrastructure to balance economic, environmental and social drivers, development of systems and indicators to measure change, reforms to EU directives on procurement, relationship between green growth strategies and sustainable diets. Consistent definitions for green public procurement and sustainable public procurement are essential to support governments at all levels. |
| --- | --- | --- | --- | --- | --- | --- | --- | --- | --- | --- | --- |
| 10 | 2016 | Nogueira, Barone, Teixeira De Barros, Guimarães, Rodrigues, Behrens. | Sixty years of the National Food Program in Brazil. | Rev. Nutr. 29 (2) 253 http://dx.doi.org/10.1590/1678-98652016000200009 | Analyse the School Food Program from the perspective of 4 basic structures: the formal structure (legal milestones that regulated the program); substantive structure (public and private social actors involved); material structure (Brazilian sponsorship of program); symbolic structure (knowledge, values, interests and rules that legitimatize the policy). | Review | Not stated | | Brazilian National School Food Program | Araraquara, Belo Horizonte, Curitiba, Porto Alegre, Praia, Rio de Janeiro, Sao Paulo (Brazil) | The evaluation of the management of the School Food Program in 5,570 Brazilian municipalities is still in its infancy and will not consider the difficulties that managers have in administrating the program. Therefore, further studies and evaluations of management of the program at a municipal level are required. |
| 11 | 2018 | Neto & Caldas | The use of green criteria in the public procurement of food products and catering services: a review of EU schemes | Environ Dev Sustain (2018) 20:1905–1933 https://doi.org/10.1007/s10668-017-9992-y | Provide a first insight on the use of green public procurement (GPP) criteria within public purchasing in Europe for national, regional or local governments. This review allows identification of the current practices, main issues and the problems faced. | Review | National, regional and local government authorities, key stakeholders involved in food service at public facilities | Programme EcoBuy (Vienna), Organic & Seasonal Procurement (Copenhagen), Sustainable food procurement for schools (Rome), Organic, seasonal food for kindergartens (Barcelona) | | Vienna (Austria), Copenhagen (Denmark), Rome (Italy), Barcelona (Spain) | 23 GPP schemes were identified across Europe; 8 National, 3 regional and 10 local level. GPP criteria apply to the provision of both food products and catering services. The main food products covered by the criteria are fruits, vegetables, dairy products, fish, seafood and meat. The criteria identified comprehensively cover the life cycle stages of the food supply chain. |
| 12 | 2016 | Olsson, Kerselaers, Kristensen, Primdahl, Rogge and Wästfelt | Peri-Urban Food Production and Its Relation to Urban Resilience | Sustainability doi:10.3390/su8121340 8, 1340 | (i) Analyse peri-urban land use changes in relations to food production and food security, (ii) Clarify connections between food production, urban resilience and multi-functional land use in this urban context. | 3 Case Studies; Gothenburg in Sweden, Copenhagen in Denmark and Gent in Belgium (all peri-urban). Mixed-methods data collection; qualitative interviews, document analysis and group discussion with stakeholders. | Farmers, city planners, officials responsible for municipality owned land and management of protected areas. | Gent' Local Food Strategy | | Gent (Belgium) | Three developmental trends were highlighted: (i) Peri-urban arable land was transferred into land for recreational purposes; (ii) Remaining peri-urban farms are increasing in scale however food production is not specifically intended for urban consumption; (iii) New forms of niche production that take advantage of the peri-urban location and control labour costs in new ways is occurring (e.g., self-harvesting by consumers and on-farm sale). |

| 13 | 2015 | Moragues-Faus & Morgan | Reframing the foodscape: the emergent world of urban food policy | Environment and Planning A 2015, volume 47, pages 1558 – 1573 doi:10.1177/0308518X15595754 | Examine the potential of cities as new urban ‘spaces of deliberation’ to foster transitions towards more sustainable and just food systems, paying particular attention to new governance configurations. | Case studies; Bristol and Malmo. Mixed-methods; data collected from CoPs - short stories, knowledge hub and lively forum. Data was analysed from project meetings and interviews with stakeholders (n=12) involved in food initiatives in both cities. Document analysis - policy documents, newsletters, webpages, reports. | Stakeholders involved in the development and implementation of food initiatives. | Bristol's Sustainable Food Strategy (2009), Bristol Good Food Plan (2013) and Bristol's Good Food Charter | Bristol, UK | Analysis in these two “innovative” cities identified that attention needs to be paid to the potential transfer of state capacities to other scales and actors (e.g. at different levels of government), fuelling uneven geographies of winning and losing cities. “Negotiating a progressive course in an ‘age of austerity’ constitutes the key political challenge to the construction of an inclusive, place-based, trans-local movement that strives to build a sustainable food system for all.” |
| --- | --- | --- | --- | --- | --- | --- | --- | --- | --- | --- |
| 14 | 2017 | Ilieva | Urban Food System Strategies: A Promising Tool for Implementing SDGs in Practice | Sustainability 2017, 9, 1707; doi:10.3390/su9101707 | Explore the alignments and potential synergies between Urban Food System Strategies (UFSS) and the 2030 Agenda. | Systematic review and comparative analysis of the 2030 Agenda and the sustainable food systems strategies of five of the 10 largest cities in North America | New York, Philadelphia, Los Angeles, Chicago & Toronto. Policy-makers, government authorities, Agenda 2030 stakeholders | FoodWorks 2010 (New York), GO TO 2040, Food Systems Report & Hunger (Chicago), A Recipe for Healthy Places 2013 (Chicago) & Cultivating Food Connections 2010 (Toronto) | New York & Chicago (USA) & Toronto (Canada) | Urban food systems strategies provide effective mechanisms to streamline global, national, and local implementation efforts towards the 2030 Agenda. This goal- and indicator-level analyses revealed promising areas for collaboration. |
| 15 | 2017 | Hawkes & Halliday | What makes urban food policy happen? Insights from five case studies | Report by the International Panel of Experts on Sustainable Food Systems (IPES-Food) http://www.ipes-food.org/images/Reports/Cities_full.pdf | Three aims: (i) What factors have enabled urban food policies to be developed and delivered? (ii) What are the barriers to developing and delivering these policies? (iii) What can be learned from these experiences for cities at different stages of policy development about how to harness the enablers and overcome barriers to make change happen? | Case Studies, mixed-methods; document analysis (policy documents, minutes from council meetings and other organizations, websites of local authorities and other organizations, media reports, and academic articles) and semi-structured interviews (with two actors involved with the development and/or implementation of the policy and a third interview was carried out where an additional point of view was deemed necessary). | Stakeholders involved in developing and delivering the following urban food policies: Belo Horizonte’s approach to food security, Nairobi Urban Agriculture Promotion and Regulation Act, Amsterdam’s Approach to Healthy Weight, Golden Horseshoe Food and Farming Plan, Detroit’s urban agriculture ordinance | Belo Horizonte’s food security policy (Brazil), Nairobi Urban Agriculture Promotion and Reg Act 2015 (Kenya), Micro-gardens programme (Dakar, Senegal), Public Policy on Food Security, Food Sovereignty and Nutrition (Medellin, Colombia), Participatory Urban Agriculture Project (Quito, Ecuador), Bristol Food Policy Council (UK), Re-using cooking oil (Rio de Janeiro, Brazil), Zero waste (San Francisco, US), Flagship Food Boroughs in London (UK), Toronto Food Strategy (Canada), New York City Food Standards (US), Urban agriculture policy (Cape Town, South Africa), Street food safety training (Abidjan, Cote d’Ivoire), Food traceability platform (Shanghai, China) | Belo Horizonte & Rio de Janeiro (Brazil), Nairobi (Kenya), Amsterdam (The Netherlands), Dakar (Senegal), Medellin (Columbia), Quito (Ecuador), Bristol & London (UK), San Francisco & New York (USA), Toronto (Canada), Cape Town (South Africa) | 15 factors were identified across the in-depth analysis of these 5 case studies. These 15 factors enabled the development and delivery of urban food policies and were categorised into six themes; (i) data, monitoring and learning, (ii) 'vertical' multi-level governance, (iii) 'horizontal' city level governance, (iv) participatory policy process, (v) funding and (vi) political commitment. |
| 16 | 2017 | Gorrie | Residential Organics Diversion in Toronto | BioCycle August 2017, Vol. 58, No. 7, p. 24 | Inform the general public about Toronto's waste trends and plans for waste reduction; racoon-resistant bins, anaerobic digestor and the Long-Term Waste Reduction Strategy | Not a study. | General public | Love Food Hate Waste as part of Toronto's Long-Term Waste Reduction Strategy (2016) | Toronto | 90 percent of Toronto’s 461,000 single family households (including those that live in apartments above commercial properties) participate in the green bin program, which now also extends to 65 percent of the City’s multifamily residential buildings. |
| 17 | 2015 | Goncalves, da Cunha, Stedefeldt & de Rosso | Family farming products on menus in school feeding: a partnership for promoting healthy eating | Rural Sociology, v 45, n 12, p2267-2273 http://dx.doi.org/ID.1590/0103-8478cr20150214 | Characterise the process of buying Family Farming (FF) food for the Brazilian School Feeding Program (BSFP) and compare menu quality before and after the implementation of the law which requires >30% of Brazilian government funds be used to buy food directly from family farmers. | Observational cross-sectional study. Semi-structured questionnaire and menu evaluation. | 82 cities from Sao Paulo and Rio de Janeiro participated. | Brazilian National School Food Program | Rio de Janeiro, Sao Paulo | The quality of the menus offered to schoolchildren improved significantly after the implementation of FF purchases. Low farmer interest and deficient hygienic conditions were the main difficulties reported. Partnership between FF and BSFP can achieve healthy eating habits by increasing nutritional quality of menus and integrating sustainability & nutritional education activities into schools. |
| 18 | 2018 | Freudenberg, Willingham & Cohen | The role of metrics in food policy: Lessons from a decade of experience in New York City | Journal of Agriculture, Food Systems and Community Development. 8(Suppl. 2), 191-209. doi:10.5304/jafscd.2018.08B.009 | Assess how annual metrics can describe a city’s implementation progress of municipal food policies over the last decade. Analysis examines: (i) changes in the indicators that the city reports; (ii) strengths and weaknesses of the Food Metrics Reports as a tool for monitoring policy enactment & impact; (iii) opportunities to improve implementation of future metrics. | Quantitative analysis of data from six food metrics reports. This analysis is used to assess progress in achieving five broad food policy goals: improving nutritional well-being, promoting food security, creating food systems that support economic and community development, ensuring a sustainable food system, and supporting food workers. | Stakeholders involved in collecting, interpreting and collating data and evaluation results related to the effectiveness of urban food policy interventions. | New York City Food Standards | New York (USA) | Recommendations to improve metrics include development of a comprehensive, intersectoral multi-year food plan. International partnerships such as the MUFPP and recent reports on urban food policy governance suggest approaches to using data to inform municipal food planning. Effective metrics are vital to increase the likelihood that, five or ten years from now, evidence can demonstrate substantial progress in creating healthier, more efficient, more equitable, and more sustainable urban food systems. |
| 19 | 2016 | Fesenfeld | Governing Urban Food Systems in the Long Run: Comparing Best Practices in Sustainable Food Procurement Regulations (SFPR) | GAIA - Ecological Perspectives for Science and Society. Vol 25, No 4, pp260-270. http://dx.doi.org/10.14512/gaia.25.4.8 | Explain the variation in target ambitiousness and successful implementation of 13 officially adopted food policies in the cities of Zurich (Switzerland), Munich and Nuremberg (Germany). | Most similar cases design to test Jabobs' three conditions for long-term policy-making theory. Document analysis and semi-structured interviews (n=24) with decision-makers. | Zurich, Munich and Nuremberg cities – all have adopted and implemented SFPR and have high contextual similarity (state/political system, geography & socio-demographics). | SFPR - increase procurement of fair-trade products (2010) & SFPR to increase procurement of organic, seasonal and less energy-intensive products in city departments and canteens (2014) | Zurich (Switzerland) | Recommendations for successful SFPR implementation: (i) Multi-sectorial governance to embed SFPRs within comprehensive urban food strategies, (ii) Coordination from authoritative, food policy coordinators from the mayor’s office, (iii) Participation of executive staff in policy network and training events, (iv) Engagement with evidence-based policy instruments by decision-makers, (v) Design policies and policy mixes that generate short-term co-benefits for decision-makers, stakeholders and citizens. |
| 20 | 2017 | Dubbeling, Santini, Renting, Taguchi, Lançon, Zuluaga, de Paoli, Rodriguez and Andino | Assessing and Planning Sustainable City Region Food Systems: Insights from Two Latin American Cities | Sustainability, 9, 1455; doi:10.3390/su9081455 | Analyse the content, definitions and limitations of the concept of City Region Food Systems (CRFS). | Case studies - Medellin and Quito. Data is derived from the FAO and RUAF programme of assessing and planning CRFS currently implemented in 8 city regions. | Policy-makers, government stakeholders | Medellin's 'Plan for Food and Nutrition Security' (2016-2028), Quito's 'AGRUPAR' (Participatory Urban Agriculture programme), Bristol's Good Food Action Plan) | Medellin (Columbia), Quito (Ecuador), Bristol (UK) & Toronto (Canada) | CRFS is a promising approach to support local governments, policy makers, and multi-stakeholder bodies to improve urban and regional food system sustainability and resilience. Recommendations based on case study analysis: (i) Strategies for Improving the Sustainability and Resilience of CRFS; (ii) Mapping and Assessing CRFS; (iii) Need for developing Integrated Territorial Governance Mechanisms. |
| 21 | 2016 | Crivits, Prové, Block & Dessein | Four Perspectives of Sustainability Applied to the Local Food Strategy of Ghent (Belgium): Need for a Cycle of Democratic Participation? | Sustainability, 8, 55; doi:10.3390/su8010055 | Explore, analyse and valorise the potential of actors, motives and logics for change within the agriculture and food system in the Ghent region, considering the four perspectives of sustainable development. | Qualitative; in-depth interviews with key informants in the food and agriculture system (n=32), participant observation of debates, workshops and meetings of the Urban Agriculture working group, document analysis of policy and informal documents, comparative case study of urban agriculture in Philadelphia (USA) and Warsaw (Poland) | Policy-makers, government stakeholders | Ghent en Garde (Gent's Local Food Strategy) | Gent (Belgium) | Applying the four perspectives of sustainability (Planetary Boundaries, Safe & Just Operating Space, Energetic Society, and Green Competition) revealed two strategies for effective governance of sustainable urban development: (i) a governance approach to stimulate participation and representation in a complex, unequal and rapidly changing context; (ii) a reflection on how considering the regional food system as a governance principle can direct action in an interconnected, globalised world. |
| 22 | 2018 | Collé, Daniel and Aubrya | Call for projects "Parisculteurs": catalyst for urban agriculture development on rooftops in Paris | Acta Hortic. 1215. ISHS 2018. DOI 10.17660/ActaHortic.2018.1215.28 | Examine the relevance of urban rooftop farming (URF) in the city of Paris in view of its specificities and to understand the management of the call for projects as an innovative way to develop urban rooftop farming. | Document analysis of the submissions received by City of Paris, in response to their "Parisculteurs" call for urban agriculture (UA) projects | Citizens, policy-makers, Government stakeholders | Main Verte (Green Hand) Charter (2003), Paris Strategy for Sustainable Food (2018) | Paris (France) | This call for URF projects was an effective mechanism to ‘green the city’ quickly and in an economically efficient manner. Submissions highlighted the diversity UA approaches and allowed the City of Paris to test technical and regulatory limits of UA on existing buildings. Developing UA by providing access to land on rooftops (without financing the winning projects) increased the professionalisation of urban agriculture in Paris. |
| 23 | 2018 | Cerutti, Ardente, Contu, Donno and Beccaro | Modelling, assessing, and ranking public procurement options for a climate-friendly catering service | Int J Life Cycle Assess (2018) 23:95–115 DOI 10.1007/s11367-017-1306-y | Determine which green public procurement policies are most efficient in reducing the global warming potential of the public service by (i) assessing a set of procurement policies, and (ii) ranking them according to their potential to reduce greenhouse gas (GHG) emissions of public catering. | Case study - a life cycle approach applied to a baseline scenario to quantify the GHGs produced by the entire catering service (including all stages of food production, management of waste, etc in kitchens and canteens). Then 11 policies were applied to this baseline scenario to assess the potential improvement. | School catering service in the City of Turin (Italy). | INNOCAT project - procurement of eco-innovation in the catering sector implemented in Turin Schools | Turin (Italy) | Among the selected policies, a change in diet was the most effective (32% reduction of emissions), followed by the adoption of improved food production practices (11% reduction) and the purchasing of certified green electricity (6% reduction). Decision-makers could benefit from basing their decisions upon scientific evidence and avoiding the prioritisation of policies based on personal opinions or weak evidence. |
| 24 | 2016 | Camps-Calvet, Langemeyer, Calvet-Mir & Gómez-Baggethun | Ecosystem services provided by urban gardens in Barcelona, Spain: Insights for policy and planning | Environmental Science and Policy, 62, pp14-23 http://dx.doi.org/10.1016/j.envsci.2016.01.007 | (i) Identify and characterize ecosystem services (ES) provided by urban gardens, (ii) assess the demographic and socioeconomic profile of gardeners and the importance they attribute to different ES, and (iii) explore the role that ES from urban gardens can play as solutions for urban policy challenges. | Single site case study. Qualitative. Document analysis (policy documents and grey literature), semi-structured interviews (n=44), survey, observation (participant and non-participant engagement with gardening tasks), 2 meetings. | Gardeners (Network of Community Gardens of Barcelona members), policy-makers and city planners | Barcelona's Green Infrastructure and Biodiversity Plan 2020 | Barcelona (Spain) | 20 ecosystem services were identified of which cultural (non-material benefits people derive from their interaction with nature) stood out as most highly valued. Main beneficiaries are older, low-middle income, and migrant people. Urban gardens can address several urban policy challenges in cities by creating opportunities for recreation, healthy lifestyles, and social cohesion. |

| 25 | 2017 | Bügel | Public procurement as a means to link sustainable production with diet and lifestyle - the new Nordic diet way | Ann Nutr Metab 2017;71(suppl 2):1–1433 | Describe how transformation to organic produce can change diets to more healthy and sustainable diets, without increasing costs. | Method not stated. Case study of the municipality of Copenhagen, who declared in 2001 that by 2011 75% of food served by public kitchens should be from organic production. In 2007 the goal was raised to 90%. | Not stated | Copenhagen's organic ag in public food service - managed by org: "Kobenhavns Madhus" | Copenhagen (Denmark) | In 2019 the goal that 90% of public meals should be made from organic produce was reached. Facilitators of success: political decision and staff capacity/willingness to increase seasonal produce and limit waste. Mandatory change to organic food, without increasing cost, results in changes towards more healthy diets. This Copenhagen model has since been implemented in other municipalities of Denmark. |
| --- | --- | --- | --- | --- | --- | --- | --- | --- | --- | --- |
| 26 | 2016 | Campbell | Getting farming on the agenda: Planning, policymaking, and governance practices of urban agriculture in New York City | Urban Forestry & Urban Greening 19 (2016) 295–305 http://dx.doi.org/10.1016/j.ufug.2016.03.011 | (i) Examine the power-laden operation of urban environmental governance, (ii) explore several ‘faces of power,’ including overt authority, institutionalized ‘rules of the game,’ and hegemony, (iii) investigate the interaction of multiple actors in policy-making processes, including through the construction and use of broad discursive concepts. | Case study; document analysis, semi-structured interviews (n=43) | Stakeholders engaged in food systems policy-making | PlaNYC2030 - New York City's Sustainability Plan, PlanNYC 2.0, Food-NYC, Food in the Public Interest, FoodWorks | New York City (USA) | Some interviewees questioned the significance of UA, due to the challenges of quantifying its benefits and minimal access to open spaces in the developed city. Despite this, a coalition of civic activists worked with public officials to develop food policy that included UA. Tracing policy implementation highlights the way the food systems concept and specific policy proposals were repeated and legitimised. Investigating the dynamics within this policymaking process provides valuable insight into how urban environmental governance occurs. |
| 27 | 2018 | Balzaretti, Ventura, Ratti, Ferrazzi, Spallina, Carruba & Castrica | Improving the overall sustainability of the school meal chain: the role of portion sizes | Eating and Weight Disorders - Studies on Anorexia, Bulimia and Obesity https://doi.org/10.1007/s40519-018-0524-z | Analyse the meal supply in Italian primary schools to identify inefficiencies within the food chain, regarding the size of the food portions specified in public tenders (which can affect the sustainability of school meals, contribute to childhood overweight and obesity and increase food waste). | Document analysis of contracts between municipalities and school catering services. Extraction of portion sizes (in grams) of the main food products included in school menus for each regional capital in Italy. Analysis considered two main factors (i) consistency of food portions within regions and (ii) adherence to national standards for children. | School food service stakeholders, municipal-level government authorities | National Guidelines for School Catering (Italy) | Milan, Bologna, Rome, Ancona, Bari and Cagliari (Italy) | Great discrepancies in portion sizes exist between and amongst regions. In some cases, portion sizes are significantly larger than the reference values of standard portions for school catering. School meals programs play a role in educating children about environmental challenges (e.g. food waste—by introducing the best strategies for waste reduction, reuse, and recycling). Benefits include economic, social, health, and environmental outcomes, highlighting the need to review existing policies and introduce new solutions for more sustainable and healthy school canteens in Italy. |
| * Citations are provided in the Reference List of Included Studies (final page of these Supplementary Materials) | | | | | | | | | | |

# Supplementary Material S6: Summary of data extracted from the policy documents cited in included studies

| *Region* | *Country* | *Signatory City* | *Policy* | *Ref* | *Page* |
| --- | --- | --- | --- | --- | --- |
| East Asia & Pacific | Nil | Nil | Nil | - | - |
| Europe & Central Asia | Austria | Vienna | OkoKauf Wien ('EcoBuy') Green Public Procurement program (2012) | i | 21 |
|  | Belgium | Gent | Gent Local Food Policy "Ghent en Garde" (2016) | ii | 22 |
|  | Denmark | Copenhagen | Organic Programme - Copenhagen House of Food (2013) | iii | 23 |
|  | France | Paris | Topager "WildRoof" (2013) | iv | 24 |
|  |  |  | Paris Strategy for Sustainable Food (2018) | v | 25 |
|  |  |  | Main Verte ‘Green Hand’ Charter (2003) | vi | 27 |
|  | Italy | Multiple | National Guidelines for School Catering, Italy (2010) Ancona, Bari, Bologna, Cagliari, Milan, Rome | vii | 28 |
|  |  | Bologna | Community rooftop garden of Via Gandusio (2011) | viii | 29 |
|  |  | Catania | ‘Orti e arte’ - Vegetable Gardens and Arts (2018) | ix | 30 |
|  |  | Rome | Sustainable Food Procurement for Schools in Rome (2013) | x | 31 |
|  |  | Turin | City of Turin: Guidelines on school canteen services (2015) | xi | 32 |
|  | Netherlands | Rotterdam | Edible City Solution initiatives (2018) | xii | 33 |
|  | Spain | Barcelona | Seasonal food for kindergartens (2013) | xiii | 34 |
|  |  |  | Barcelona green infrastructure and biodiversity plan (2020) | xiv | 36 |
|  | Switzerland | Zurich | Sustainable Food Procurement Regulations in Zurich City (2010) | xv | 37 |
|  | UK | Bristol | Bristol's Good Food Action Plan (2015) | xvi | 38 |
|  |  | Brighton & Hove | Spade to spoon: digging deeper. A food strategy and action plan for Brighton and Hove (2012) | xvii | 40 |
|  |  | London | Flagship Food Boroughs in London (2014) | xviii | 41 |
|  |  |  | The London Food Strategy (2018) | xix | 42 |
| Latin America & Caribbean | Brazil | Multiple | National School Feeding Program of Brazil - Sustainable Schools initiative (2009) Araraquara, Belo Horizonte, Curitiba, Porto Alegre, Praia, Rio de Janeiro, Sao Paulo | xx | 43 |
|  |  | Belo Horizonte | Belo Horizonte food security policy (2011) | xxi | 45 |
|  | Columbia | Medellin | Public Policy on Food Security, Food Sovereignty and Nutrition (2005) | xxii | 46 |
|  | Ecuador | Quito | Participatory Urban Agriculture Project (AGRUPAR) (2000) | xxiii | 47 |
| Middle East & North Africa | Nil | Nil | Nil | - | - |
| North America | Canada | Toronto | Toronto Food Strategy (2010) | xxiv | 48 |
|  |  |  | Toronto Golden Horseshoe Food and Farming Action Plan (2012) | xxv | 49 |
|  |  |  | Toronto's Long-Term Waste Management Strategy (2016) | xxvi | 51 |
|  |  | Vancouver | Regional Food System Action Plan Metro Vancouver (2016) | xxvii | 52 |
|  | United States of America | Chicago | Chicago: GO TO 2040 Regional Comprehensive Plan Chicago (2010) | xxviii | 53 |
|  |  |  | A Recipe for Healthy Places: Addressing the Intersection of Food and Obesity in Chicago (2013) | xxix | 54 |
|  |  | New York City | Foodworks: a vision to improve NYCs food system (2010) | xxx | 56 |
|  |  |  | New York City Food Standards (2011) | xxxi | 57 |
|  |  | San Francisco | Healthy & Sustainable Food for San Francisco (2009) | xxxii | 59 |
|  |  |  | San Francisco Zero Waste (2018) | xxxiii | 60 |
| South Asia | Nil | Nil | Nil | - | - |
| Sub-Saharan Africa | Kenya | Nairobi | Nairobi Urban Agriculture Promotion and Regulation Act (2015) | xxxiv | 61 |
|  | Senegal | Dakar | Dakar Micro-gardens Programme (2006) | xxxv | 62 |
|  | South Africa | Cape Town | Urban Agriculture Policy (2007) | xxxvi | 63 |

*NB: The following tables are linked to the reference list on page 65, with full details of the included studies that each policy was cited within*

| Citation | Name of Policy | **OkoKauf Wien ('EcoBuy') Green Public Procurement program (2012)** |
| --- | --- | --- |
|  | Reference | i |
|  | Format and link or citation | Website: <https://www.wien.gv.at/english/environment/protection/oekokauf/criteria-catalogues.html#food> |
|  | Included study(s) cited within | 9, 11 |
| Geographic context | Region | Europe & Central Asia |
|  | Country | Austria |
|  | Signatory City | Vienna |
|  | World Bank economic classification | High Income |
| Policy context | Is this intervention a policy itself or part of an over-arching policy? | Regulatory policy |
|  | Timeframe for intervention | The food criteria available online are dated from 2010 and 2012 with no end date stated. |
|  | Related policy interventions (Regional, National, State, Local) | The ‘OkoKauf Wien’ (‘EcoBuy’) programme, with targets for organic food procurement, was launched as part of the Vienna Climate Protection Programme (KliP Wien) in 1999. [City of Vienna website](https://www.wien.gv.at/english/environment/protection/oekokauf/index.html). Animal Welfare Act (Bundestierschutzgesetz) Federal Law Gazette I No 118/2004, 1996 Regulation on Packaging, Federal Law Gazette (BGBl.) II No 648/1996, Federal Ministry of Health (trans fatty acid regulation), Federal Law Gazette II No 267/2009, Austrian Federal Procurement Act of 2006 (Bundesvergabegesetz, BVergG) |
| Policy Aims & Activities | Policy aim | Promote climate protection within the City of Vienna by following ecological criteria when guying goods, products and services in all areas of the City Administration. The Food criteria relate to the procurement of (i) food and products of organic agriculture, (ii) food with the lowest possible share of artificial trans fatty acids, (iii) fresh hen eggs (shell eggs), pasteurised whole hen eggs, hen egg yolk, hen egg white of free-range hens. |
|  | Description of actions | Activities related to food require the inclusion of these criteria in any tender documentation for facilities of the City of Vienna: (i) at least 30% (monetary assessment) of food purchased should originate from organic farming, (ii) organic labelling on food must meet EU Council Regulations, (iii) fruit and vegetables should be in season and from the region where possible, (iv) Beverages, fruits and vegetables should be delivered in reusable transport packaging where possible, (v) no food is procured with more than 2% trans fatty acids (with regard to fat content), (vi) tenderers must advise which collection and recycling system they are part of to define the measures they're taking to put essential food packaging back into circulation. (vii) labelling requirements to enhance the traceability of eggs determined for human consumption (method of production, country code and registration number), (viii) proof of free-range production for all eggs. |
|  | Role of Local Government | Leadership/ownership. The City of Vienna established the OkoKauf programme in 1998. |
|  | Targeted healthy and sustainable diet-related practice(s) | More plant-based foods  Eat seasonally  Eat locally available foods  Minimise food waste  Support sustainable food production practices |
|  | Targeted food system phase(s) | All |
| Considerations | Does the policy consider health? Y/N and how? | Yes - the trans-fat regulations are intended to prevent diet-related disease |
|  | Does the policy consider equity? Y/N and how? | Not stated |
|  | Does the policy consider the broader food system? Y/N and how? | Yes - promotion of organic food production, promotion of ethical egg production, efforts to shorten the supply chain, promotion of seasonal produce |
| Evaluation | Has evaluation been planned for? | Each of the activities is presented with clear targets and adequate detail to discern how the monitoring and evaluation of progress could be approached. |
|  | Data collection tools, target population | The approach taken to evaluation (including tools) is not described in this policy document, nor in any of the included studies of this review. |
|  | Has the policy been effective? | From the evaluation data provided on the linked website, more than 740 local businesses have participated in the program since 1998 and have saved energy, raw materials, water and reduced waste output. From the data presented by Smith et al (2016), this intervention has affected the supply of food to approximately 85 thousand people in hospitals, schools, kindergartens and nursing homes. The overall spend on organic food procurement is stated as more than 50% (more than 90% for dairy products) thus exceeding the 30% target. Smith et al (2016) also state that this intervention saved the City of Vienna EUR 44.4 million and over 100000 tonnes of carbon dioxide emissions between 2001 and 2007. |
| Policy Development | What evidence underpins the development of the policy? | Decades of practical experience and monitoring data from the National intervention. |
|  | What type of evidence was used to develop the policy? | Not stated |
|  | What was the process for using evidence to develop the policy? | J Smith et al (2016) describe the policy-making process for this intervention using a case study approach. They describe a range of political (cross-departmental commitment, funding, international recognition), environmental (Vienna's Climate Protection Programme) and social (motivating more sustainable purchasing amongst procurement officers, motivating chefs to cook from scratch) forces that enabled implementation. |

| Citation | Name of Policy | **Gent Local Food Policy "Ghent en Garde" (2016)** |
| --- | --- | --- |
|  | Reference | ii |
|  | Format and link or citation | [Report of recommendations](https://stad.gent/sites/default/files/page/documents/20160913_PU_Gent%20en%20garde_operationele%20doelstellingen_Engels_web.pdf) from the Food Policy Council |
|  | Included study(s) cited within | 12, 21 |
| Geographic context | Region | Europe & Central Asia |
|  | Country | Belgium |
|  | Signatory City | Gent |
|  | World Bank economic classification | High Income |
| Policy context | Is this intervention a policy itself or part of an over-arching policy? | Recommended Action Plan |
|  | Timeframe for intervention | Policy adopted for period 2013-2018. These recommendations for an action plan were published in 2016. No end date stated, “it's an ongoing process”. |
|  | Related policy interventions (Regional, National, State, Local) | Food Smart Cities for Development Project (EU), Sustainable Development Goals, Milan Urban Food Policy Pact |
| Policy Aims & Activities | Policy aim | Five strategic goals to create a sustainable food system for Ghent; (i) A shorter, more visible food chain, (ii) More sustainable food production and consumption, (iii) The creation of more social added value for food initiatives, (iv) Reduce food waste, (v) Optimum reuse of food waste as raw materials. The Gent en Garde food policy council was established with 25 members from various sectors (agriculture, knowledge institutions, commerce, etc) to act as a sounding board and ensure effective implementation of the city's policy on food. This report describes their effort to translate the 2013 policy into concrete operational goals. |
|  | Description of actions | Operational goals include activities to access local products (farmers markets), create spaces for professional food production, awareness raising amongst residents to eat less meat, opt for organic, fair trade, local and seasonal food, update tender processes for public facilities (schools, hospitals, companies), serve sustainable food at events (currently only vegetarian food is served on Thursdays at publicly funded events 'Thursday Veggie Day'), school served a 'LEF' lunch - Local, Environmentally-friendly, Fair-trade (LEF) supplied by 5 local farmers, urban agriculture hub with training and networking, produce gardens in schools, social employment through food (social restaurants, grocers and in processing/distribution of food), recover and redirect surplus food to emergency food relief, compostable boxes in restaurants to encourage take-away leftovers (program coordinated by city council), closing waste cycles by encouraging on-site composting at home and in the neighbourhood and promoting new business models e.g. oyster mushrooms from coffee grounds |
|  | Role of Local Government | Leadership/ownership. City of Ghent established the food policy council to lead this work and two of the 25 council members work for the City of Ghent. |
|  | Targeted healthy and sustainable diet-related practice(s) | Less animal-derived foods  More plant-based foods  Eat seasonally  Eat locally available foods  Minimise food waste  Connect with local food system  Support sustainable food production practices |
|  | Targeted food system phase(s) | All |
| Considerations | Does the policy consider health? Y/N and how? | Yes - promoting less meat and more fruits and vegetables, making school meals healthier and emergency food relief meals healthier |
|  | Does the policy consider equity? Y/N and how? | Yes - improving access to healthy and environmentally sustainable food for people experiencing food insecurity |
|  | Does the policy consider the broader food system? Y/N and how? | Yes - promotion of fair-trade, seasonal, organic food, reducing food waste, shortening food supply chains, promoting alternative retail options, reintroducing food waste back into the system |
| Evaluation | Has evaluation been planned for? | Evaluation is not described in this document and the operational goals are not written in a level of detail whereby they are measurable, as this document is intended to provide recommendations for further consideration. |
|  | Data collection tools, target population | The approach taken to evaluation (including tools and results) is not described in this policy document, nor in any of the included studies of this review. |
|  | Has the policy been effective? | Progress towards achieving the intended goals is not stated in the recommendations report nor in either of the relevant included studies. |
| Policy Development | What evidence underpins the development of the policy? | The Food Policy Council's report cites many global and national sources of expertise. |
|  | What type of evidence was used to develop the policy? | The document describes various stakeholder discussions, input from the city administration and political agreement. |
|  | What was the process for using evidence to develop the policy? | The details of seeking stakeholder input were not stated. |

| Citation | Name of Policy | **Organic Programme - Copenhagen House of Food (2013)** |
| --- | --- | --- |
|  | Reference | Iii |
|  | Format and link or citation | [Website, case study document, YouTube videos](http://base.citego.org/docs/fiche_copenhagen.pdf) |
|  | Included study(s) cited within | 9, 11, 25 |
| Geographic context | Region | Europe & Central Asia |
|  | Country | Denmark |
|  | Signatory City | Copenhagen |
|  | World Bank economic classification | High Income |
| Policy context | Is this intervention a policy itself or part of an over-arching policy? | Policy - regulatory |
|  | Timeframe for intervention | 2013-2020 |
|  | Related policy interventions (Regional, National, State, Local) | Organic food in public procurement has been on the political agenda in Denmark since the 1990s as part of their sustainable food strategy. Since 2009 the Organic Programme has been administered by 'Copenhagen House of Food'. "Eco-Metropolis: Our Vision for Copenhagen 2015" states that 'there will be at least 20% organic food in the city's food consumption, with the city taking the lead with at least 90% organic food in municipal institutions'. |
| Policy Aims & Activities | Policy aim | Two main objectives; (i) 20% organic food in the city's food consumption, (ii) the city leads the way with at least 90% organic food in its institutions. |
|  | Description of actions | ‘Copenhagen House of Food' have a team of chefs, food specialists, generalists, teachers, project managers, communicators, ethnologists, designers etc. who work with staff in public kitchens. They provide consultancy, training, support and communication to public kitchens and are experienced in 'organic conversion'. They base their approach on these 10 principles; (i) Less meat and different meat (use the whole animal, also the cheap cuts), (ii) More vegetables - greens in season, diversity, (iii) More potatoes - better potatoes, (iv) Fruit in season - fruit alone is not enough, (v) More or different use of bread of grains, (vi) Beware of the sweet and expensive, (vii) Composition of menus - difference between every day and feast, (viii) Old housekeeping virtues - rational kitchen operation (less waste), (ix) Critical use of full- and semi-manufactures, more ingredients, (x) Find the weak point, one or more of the above. FIVE MAIN STRATEGIES: 'Kitchen Lift' - a tool for change in kitchens, Urban Agriculture - still in infancy, Educating and empowering future generations, Eat-Cuisine: accommodation to context and ambition of food education, Food schools. |
|  | Role of Local Government | Leadership. City of Copenhagen led the policy development and have invested in capacity building activities to increase organic food consumption in their jurisdiction. 'Københavns Madhus (Copenhagen House of Food) is an independent, non-commercial foundation established by the City of Copenhagen in 2007. It inherited the “Copenhagen Healthy School Meals” (see above) and has been working over the 900 public kitchens preparing meals for the city public food services in kindergartens, schools, social institutions, elderly homes and staff restaurants |
|  | Targeted healthy and sustainable diet-related practice(s) | Variety (diversity)  Avoid over-consumption  Limit processed foods  More plant-based foods  Less animal-derived foods  Eat seasonally  Eat locally available foods  Minimise food waste  Connect with local food system  Support sustainable food production practices  Prepare food at home with others |
|  | Targeted food system phase(s) | Distribution  Agricultural production  Consumption |
| Considerations | Does the policy consider health? Y/N and how? | Y - promoting organic procurement via the 'Copenhagen Healthy School Meals' initiative - the health outcomes of children and other population groups are considered throughout this policy. |
|  | Does the policy consider equity? Y/N and how? | Not stated beyond the mention that this policy includes food service for disabled, mentally ill and elderly persons. |
|  | Does the policy consider the broader food system? Y/N and how? | Y - organic food production, using the whole animal (less waste), increasing seasonal produce consumption, urban agriculture |
| Evaluation | Has evaluation been planned for? | Not stated in this document, however specific and measurable targets are set throughout. |
|  | Data collection tools, target population | Bugel (2017)'s research evaluated the success of this intervention however the data collection tools and approach to evaluation is not stated in the included study of this review (a Scientific Symposium Supplementary issue) |
|  | Has the policy been effective? | Bugel (2017)'s research revealed that transformation from conventional food products to organic in public procurement can shift diets to be healthier and more sustainable without increasing financial costs. |
| Policy Development | What evidence underpins the development of the policy? | Not stated |
|  | What type of evidence was used to develop the policy? | Not stated |
|  | What was the process for using evidence to develop the policy? | J Smith et al (2016) describe the policy-making process for this intervention using a case study approach. They describe a range of political (goals with clear timer frame, allocated finance), environmental (need to protect groundwater from pesticides, GHGE targets) and social (food literacy amongst children and young people, advocating for more healthy and sustainable food consumption) forces that enabled implementation. |

| Citation | Name of Policy | **Topager "WildRoof" (2013)** |
| --- | --- | --- |
|  | Reference | iv |
|  | Format and link or citation | Website: <http://topager.com/portfolio-item/wildroof/> |
|  | Included study(s) cited within | 4 |
| Geographic context | Region | Europe & Central Asia |
|  | Country | France |
|  | Signatory City | Paris |
|  | World Bank economic classification | High Income |
| Policy context | Is this intervention a policy itself or part of an over-arching policy? | No. This is a public-private partnership. |
|  | Timeframe for intervention | Topager was founded in 2013 and continues to operate, designing and establishing rooftop gardens. |
|  | Related policy interventions (Regional, National, State, Local) | The intervention received funding from the City of Paris as part of their call for project "Innovative Vegetation" for terracotta walls (Wild On Wall). The City of Paris' food strategy aims to increase urban agriculture https://www.api-site.paris.fr/paris/public/2018%2F9%2FENG_Abrege_StratAlim.pdf |
| Policy Aims & Activities | Policy aim | Topager's "WildRoof" initiative aims to design, build and manage rooftop gardens for food productions (fruits and vegetables). The garden design is adapted to meet intended purposes of food production, food and landscape interaction, therapeutic role, social inclusion and biodiversity increase. |
|  | Description of actions | Topager is a small company with expertise in designing open-air gardens. They have designed over 20 gardens and provide design, installation, monitoring, training and garden maintenance. Design incorporates circular economy, agroecological methods, consideration of urban composting and soil management (earthworms). Participants in this intervention include restaurants, residents, hospitals (therapeutic purposes), city halls and companies. |
|  | Role of Local Government | The City of Paris awarded a prize for the intervention which publicised and gave credibility to the company for further expansion. |
|  | Targeted healthy and sustainable diet-related practice(s) | Variety (diversity)  More plant-based foods  Eat seasonally  Eat locally available foods  Minimise food waste  Connect with local food system  Support sustainable food production practices |
|  | Targeted food system phase(s) | Agricultural production  Distribution  Consumption  Waste |
| Considerations | Does the policy consider health? Y/N and how? | Y - increasing fruit and vegetable consumption, social connectedness, therapeutic benefits of gardening |
|  | Does the policy consider equity? Y/N and how? | Not stated |
|  | Does the policy consider the broader food system? Y/N and how? | Y - contributing research and development advances to the sector eg. Determining technical and economic viability of agronomic practices in the urban context (which if effective, "could be integrated into city's policy for greening roofs and improving city's resilience") |
| Evaluation | Has evaluation been planned for? | Sanye-Mengual et al (2018) describe an evaluation of rooftop agriculture across Western Europe, including this community garden. |
|  | Data collection tools, target population | Case study research design - data collection methods not stated. |
|  | Has the policy been effective? | The garden was identified to have diverse impacts on clients of the gardens, depending on their purpose. The details of these impacts are not stated. |
| Policy Development | What evidence underpins the development of the policy? | Not stated |
|  | What type of evidence was used to develop the policy? | Not stated |
|  | What was the process for using evidence to develop the policy? | Not stated |

| Citation | Name of Policy | **Paris Strategy for Sustainable Food (2018)** |
| --- | --- | --- |
|  | Reference | v |
|  | Format and link or citation | Strategy Document: <https://www.api-site.paris.fr/paris/public/2018%2F9%2FENG_Abrege_StratAlim.pdf> |
|  | Included study(s) cited within | 22 |
| Geographic context | Region | Europe & Central Asia |
|  | Country | France |
|  | Signatory City | Paris |
|  | World Bank economic classification | High Income |
| Policy context | Is this intervention a policy itself or part of an over-arching policy? | Policy - non-regulatory |
|  | Timeframe for intervention | 2018 - 2030 |
|  | Related policy interventions (Regional, National, State, Local) | 2010 Sustainable Food Plan, 2003 Green Verte Charter, Milan Urban Food Policy Pact (signed in 2015) |
| Policy Aims & Activities | Policy aim | Paris's Strategy for Sustainable Food commits the City of Paris to "a more sustainable, more inclusive, more resilient food system" |
|  | Description of actions | The Paris Strategy for Sustainable Food is made up of 40 actions across 4 themes; (i) Access to sustainable food for everyone e.g. mobile grocery stores, prioritise transport infrastructure for businesses offering sustainable food to shorten supply chains and develop sales opportunities, facilitate access to new premises for shops from the social economy, supermarkets to promote local food products, promote sustainable food innovation start-ups via the 'Paris Smart Food' incubator, increase sustainable food on menus at canteens, assistance to restaurant owners to increase vegetarian offerings with awards to incentivise this practice called 'sustainable restaurant certificates', set up shared kitchens to allow people without such facilities to cook sustainable organic fruit and vegetables, incentivise isolated elderly people to eat at restaurants with the sustainability certificate via 'Paris Emeraude' program reserved for seniors, implement Food Aid Action Plan, introduce fruit and vegetable solidarity cards, expand 'cafe sustendu' program from cafes to include bakers and grocery stores where customers can pay for someone less fortunate to access food later, open 10 new social grocery stores, (ii) Increased autonomy and food resilience e.g. connect producers with consumers through creating 3 new organic markets, develop 'farmers' stores', launch official signs of Quality and Origin to promote local sustainable producers, preserve agricultural land, relocate food processing facilities to the outskirts of Paris, diversify local food supply through urban and peri-urban partnerships, workshops to build food processing capacity, establish 3 educational farms to deliver permaculture training, develop resilient food supply logistics, (iii) Prevention of food wastage and food waste e.g. coordination of networks to connect charities who recover and redistribute food to those in need, campaign to raise awareness of food waste reduction strategies with tailored communication tools, 900 domestic composting sites deployed to public facilities and public housing, collect food waste from households, test anaerobic digester, open bulk stores to minimise packaging, event organisers to follow eco-responsible events charter, promote tap water through posers, (iv) Networking and partnerships e.g. culinary workshops to increase consumption of fresh seasonal products and legumes, school workshops, educational farm tours, establish a 'Network of actors of sustainable food' including a digital platform and directory of projects supporting sustainable food, reinforce actions to secure position as a 'Fair Trade Town', establish a metropolitan governance of food within the City of Paris to coordinate implementation of the strategy, establish an annual 'Sustainable Food Day and Congress' to bring together key stakeholders to promote actions in the region. |
|  | Role of Local Government | Leadership/ownership. The city of Paris developed this strategy for the region by co-creating 4 themes and 40 action items with key actors in the sector. The strategy advises that implementation will involve regular collaboration with partners and citizens, charitable, voluntary and economic organisations and institutions. However, the coordination of implementation appears to remain with the City of Paris. |
|  | Targeted healthy and sustainable diet-related practice(s) | Variety (diversity)  Avoid over-consumption  Less animal-derived foods  More plant-based foods  Eat seasonally  Eat locally available foods  Minimise food waste  Connect with local food system  Support sustainable food production practices  Promote safe tap water  Prepare food at home with others |
|  | Targeted food system phase(s) | All |
| Considerations | Does the policy consider health? Y/N and how? | Y - the strategy acknowledges that Parisians have a lower obesity rate than the rest of France, greater consumption of organic products (61% consumer these regularly) |
|  | Does the policy consider equity? Y/N and how? | Y - the strategy acknowledges that Parisians living in 'priority neighbourhoods' have higher rates of childhood obesity and household food insecurity (6.3%), incentivise isolated elderly people to eat at restaurants with the sustainability certificate via 'Paris Emeraude' program reserved for seniors, implement Food Aid Action Plan, introduce fruit and vegetable solidarity cards, expand 'cafe sustendu' program from cafes to include bakers and grocery stores where customers can pay for someone less fortunate to access food later, open 10 new social grocery stores, culinary workshops in seniors' accommodation and specialised housing for people with disabilities |
|  | Does the policy consider the broader food system? Y/N and how? | Y - the strategy acknowledges that approximately 70% of food consumed by Parisians comes from France, in particular the Paris Basin. |
| Evaluation | Has evaluation been planned for? | Measurable targets (deadline is 2030) are stated in the Strategy e.g. Increase the consumption of food produced in the Paris Basin to 50% (currently 25%), Decrease the region's food carbon footprint by 40%, dedicate 20% of agricultural land to organic production (compared to 2.7%), 75% of Parisians to regularly buy organic food, reduce obesity from 10.7% to 5% (focussing on priority neighbourhoods), eliminate food insecurity from 6.3% to 0%, expand transport options to connect consumers with producers (electric vehicles, foot, bikes, river), increase proportion of sustainable food in City of Paris funded canteens and restaurants to 90% by 2050. One target is not measurable: "contribute to changing the nutritional balance of the population towards a 'flexitarian' diet." The approach to evaluation has not been described in the Summary Strategy Document not the included study by Colle et al (2018) |
|  | Data collection tools, target population | Not stated |
|  | Has the policy been effective? | Not stated |
| Policy Development | What evidence underpins the development of the policy? | The strategy describes taking learnings from the "international scope of the challenge" and promotes the City of Paris' collaborations with the 'Organic Cities Network Europe', the 'Milan Urban Food Policy Pact', 'Eurocities' and the 'C40 network' |
|  | What type of evidence was used to develop the policy? | The strategy mentions a consultation process however specific details are not included. |
|  | What was the process for using evidence to develop the policy? | According to the Strategy document, "extensive consultation" was conducted with Parisians and representative groups operating in the region's food system throughout 2017 to develop the Strategy. |

| Citation | Name of Policy | **Main Verte ‘Green Hand’ Charter (2003)** |
| --- | --- | --- |
|  | Reference | vi |
|  | Format and link or citation | [Website directory](https://www.ryerson.ca/carrotcity/board_pages/city/main_verte.html) with 3 case study gardens within the City of Paris |
|  | Included study(s) cited within | 22 |
| Geographic context | Region | Europe & Central Asia |
|  | Country | France |
|  | Signatory City | Paris |
|  | World Bank economic classification | High Income |
| Policy context | Is this intervention a policy itself or part of an over-arching policy? | Part of the Carrot City directory for 'Designing Urban Agriculture' in many cities globally |
|  | Timeframe for intervention | 2003 - present |
|  | Related policy interventions (Regional, National, State, Local) | The [summarised charter](https://translate.google.com/translate?hl=en&sl=fr&u=https://api-site-cdn.paris.fr/images/123236.pdf&prev=search) agreement itself |
| Policy Aims & Activities | Policy aim | Main Verte Charter was introduced in 2003 to 'frame the practice of wild gardening which was emerging: it was the beginning of shared gardens.' It was created with the intention of establishing more community gardens (each assigned to local associations), bringing together Parisians to practice their gardening skills and maintain biodiversity in urban areas. |
|  | Description of actions | These community gardens are mainly established on public land and most are permanent gardens. City of Paris provides landscaping services, raised garden beds or rigid planning containers, topsoil, a water supply, above-ground bins for edibles and a utility shed. The code of management (known as the "Charter Main Verte") is signed by the association to ensure the garden is open to the public at least 2 half days per week and 1 on the weekend, and whenever a gardener is present, prohibition of toxic chemicals, local composting, recovery of rainwater, reporting requirements, etc. The gardens can be intended for therapeutic purposes, social bonding between generations and cultures, integration gardens, educational gardens as well as food provision. Gardeners are part of the association who sign the charter and come from schools, hospitals, social centres, residents’ associations, colleges, early learning centres, residential facilities, etc). |
|  | Role of Local Government | Leadership/ownership is with the City of Paris' Department of Green Spaces and the Environment |
|  | Targeted healthy and sustainable diet-related practice(s) | More plant-based foods  Eat seasonally  Eat locally available foods  Minimise food waste  Connect with local food system  Support sustainable food production practices |
|  | Targeted food system phase(s) | Agricultural production  Consumption  Waste |
| Considerations | Does the policy consider health? Y/N and how? | Not stated |
|  | Does the policy consider equity? Y/N and how? | Y - the City provides raised garden beds to protect edibles from pollution and also to enable people with reduced mobility to garden also. |
|  | Does the policy consider the broader food system? Y/N and how? | Y - considers use of pesticides and fertilisers, promotes organic gardening practices |
| Evaluation | Has evaluation been planned for? | Not stated |
|  | Data collection tools, target population | Not stated |
|  | Has the policy been effective? | The website describes in brief, three case study garden locations however does not include details of outcomes. |
| Policy Development | What evidence underpins the development of the policy? | Not stated |
|  | What type of evidence was used to develop the policy? | Not stated |
|  | What was the process for using evidence to develop the policy? |  |

| Citation | Name of Policy | **National Guidelines for School Catering, Italy (2010)** |
| --- | --- | --- |
|  | Reference | Vii |
|  | Format and link or citation | European commission School Food Policy Country [Factsheet](https://ec.europa.eu/jrc/sites/jrcsh/files/jrc-school-food-policy-factsheet-italy_en.pdf) and [Official Policy](https://translate.googleusercontent.com/translate_c?depth=1&hl=en&prev=search&rurl=translate.google.com&sl=it&sp=nmt4&u=http://www.salute.gov.it/imgs/C_17_pubblicazioni_1248_allegato.pdf&usg=ALkJrhgda9Ulu8mr1HsBW0NUWo6xGN20QA) |
|  | Included study(s) cited within | 27 |
| Geographic context | Region | Europe & Central Asia |
|  | Country | Italy |
|  | Signatory City | Milan, Bologna, Rome, Ancona, Bari and Cagliari |
|  | World Bank economic classification | High Income |
| Policy context | Is this intervention a policy itself or part of an over-arching policy? | Policy - regulatory |
|  | Timeframe for intervention | 2010 - present |
|  | Related policy interventions (Regional, National, State, Local) | United Nations Decade of Action on Nutrition, UN's School Food and Nutrition Framework |
| Policy Aims & Activities | Policy aim | The National Guidelines for School Catering include objectives to i) improve child nutrition, ii) reduce/prevent malnutrition, iii) improve attainment, iv) support parents and local community, v) improve school attendance, vi) promote health and disease prevention, vii) learn about food traditions. |
|  | Description of actions | Standards included in the guidelines specify desirable parameters for portion sizes (eg. lunch should provide 25% daily energy requirement), food-based standards, nutrient-based standards, dining spaces and facilitates, staff training, food arrangement/presentation, adequate time to eat. The food-based standards relate to fresh drinking water (must be provided and easily accessible), fruit and vegetable provision (legumes 1-2 times per week, change the menu spring-summer to use seasonal produce), salt restrictions, frequency of serving dairy (cheese 1/week), frequency of serving oily fish (1-2 times per week), frequency of serving non-meat/non-dairy protein (1 egg per week), frequency of serving meat (less than 1-2 times per week). In vending machines on school premises, the School Catering Guidelines recommend and promote healthful options. However, there are no restrictions in place to limit food marketing on school premises and food and nutrition education is a mandatory part of the curriculum in Italy as part of these national guidelines. |
|  | Role of Local Government | Developed by the Italian Ministry of Health. Implemented by local government authorities. School catering is a significant budget item for local governments in Italy. |
|  | Targeted healthy and sustainable diet-related practice(s) | Variety (diversity)  Avoid over-consumption  Less animal-derived foods  More plant-based foods  Eat seasonally  Eat locally available foods  Minimise food waste  Connect with local food system  Promote safe tap water |
|  | Targeted food system phase(s) | Agricultural production  Consumption  Waste |
| Considerations | Does the policy consider health? Y/N and how? | Y - 'improve child nutrition' is the first objective and 'promote health and prevent disease' is another, consideration is made of obesity/overweight rates, portion control to avoid over-consumption, increasing fruit and vegetable intake, healthy vending machine options. |
|  | Does the policy consider equity? Y/N and how? | Y - reducing and preventing malnutrition is part of the objective, in order to offer nutritious meal options for children who may be experiencing disadvantage |
|  | Does the policy consider the broader food system? Y/N and how? | Y - supporting seasonal produce by specifying menu reviews in spring-summer |
| Evaluation | Has evaluation been planned for? | The policy is evaluated by reviewing food provision at school, take-up of school meals, financial viability of services, engagement with local farmers, control of portion sizes, continuing education of canteen staff, implementation of contract/laws, quality/price, compliance with menu requirements including special dietary requests (coeliac, etc). |
|  | Data collection tools, target population | Balzaretti et al (2018) reviewed the potential of school meals to increase childhood obesity and food waste by conducting document analysis (contracts between municipalities and school catering services). Portion size assessment. Analysis considered two main factors (i) consistency of food portions within regions and (ii) adherence to national standards for children. |
|  | Has the policy been effective? | Balzaretti et al (2018) concluded that portion sizes vary significantly between regions and in several cases, are excessive against recommendations, leading to over-consumption and food waste. They also highlight the educational potential of eating at school to address food waste—by promoting strategies for waste reduction, reuse, and recycling. |
| Policy Development | What evidence underpins the development of the policy? | Not stated in the online case study or the included paper by Balzaretti et al (2018) |
|  | What type of evidence was used to develop the policy? | Not stated in the online case study or the included paper by Balzaretti et al (2018) |
|  | What was the process for using evidence to develop the policy? | Not stated in the online case study or the included paper by Balzaretti et al (2018) |

| Citation | Name of Policy | **Community rooftop garden of Via Gandusio (2011)** |
| --- | --- | --- |
|  | Reference | Viii |
|  | Format and link or citation | [Website](https://susturbanfoods.com/2016/07/13/case-study-community-garden-of-via-gandusio-bologna-italy/) |
|  | Included study(s) cited within | 4 |
| Geographic context | Region | Europe & Central Asia |
|  | Country | Italy |
|  | Signatory City | Bologna |
|  | World Bank economic classification | High Income |
| Policy context | Is this intervention a policy itself or part of an over-arching policy? | Not stated however was designed by the Municipality of Bologna so must be considered part of a funded workplan. |
|  | Timeframe for intervention | The garden opened in 2011 and is described as the first government-funded rooftop garden of Italy. In 2017 the garden ceased due to structural renovation of the housing complex. |
|  | Related policy interventions (Regional, National, State, Local) | Not stated |
| Policy Aims & Activities | Policy aim | Via Gandusio's community garden aims to set a "meeting point for the community where food production is the link between neighbours to exchange knowledge, culture and experiences" |
|  | Description of actions | This rooftop garden exists in a social housing complex 'Via Gandusio', in the North of Bologna. Residents are 'advanced-age Italians (former migrants in the 60's) and current international immigrants from Africa and Asia. 250m2 of roof garden co-designed with residents, including do-it-yourself cultivation systems. Production was all pesticide-free and consumed by the participants. The University and education association 'L'Altra Gandusio' developed research and training activities in the garden. |
|  | Role of Local Government | The community garden was designed and funded by the Municipality of Bologna, the association 'BiodiverCity' and the RESCUE-AB (University of Bologna). Implementation costs were covered by the local government. |
|  | Targeted healthy and sustainable diet-related practice(s) | More plant-based foods  Eat seasonally  Eat locally available foods  Connect with local food system  Support sustainable food production practices |
|  | Targeted food system phase(s) | Agricultural production  Distribution  Consumption |
| Considerations | Does the policy consider health? Y/N and how? | Y - increasing fruit and vegetable consumption, social connectedness |
|  | Does the policy consider equity? Y/N and how? | Y - targeted low-income participants |
|  | Does the policy consider the broader food system? Y/N and how? | Not stated |
| Evaluation | Has evaluation been planned for? | Sanye-Mengual et al (2018) describe an evaluation of rooftop agriculture across Western Europe, including this community garden. |
|  | Data collection tools, target population | Case study research design - data collection methods not stated. |
|  | Has the policy been effective? | The garden was identified to have diverse impacts on clients of the gardens, depending on their purpose. The details of these impacts are not stated. |
| Policy Development | What evidence underpins the development of the policy? | Not stated |
|  | What type of evidence was used to develop the policy? | Not stated |
|  | What was the process for using evidence to develop the policy? | Not stated |

| Citation | Name of Policy | **‘Orti e arte’ - Vegetable Gardens and Arts (2018)** |
| --- | --- | --- |
|  | Reference | Ix |
|  | Format and link or citation | Data extracted from peer-reviewed journal article |
|  | Included study(s) cited within | 1 |
| Geographic context | Region | Europe & Central Asia |
|  | Country | Italy |
|  | Signatory City | Catania |
|  | World Bank economic classification | High Income |
| Policy context | Is this intervention a policy itself or part of an over-arching policy? | This urban garden is part of the EU's over-arching policy intervention to promote urban agriculture |
|  | Timeframe for intervention | Not stated |
|  | Related policy interventions (Regional, National, State, Local) | Part of European-wide policy: <https://eur-lex.europa.eu/legal-content/EN/ALL/?uri=CELEX%3A52016IR3170> which has since informed the development of iPES Food's "Towards a Common Food Policy for the European Union": <https://www.askfood.eu/tools/forecast/wp-content/uploads/2019/07/CFP_FullReport.pdf> |
| Policy Aims & Activities  Considerations | Policy aim | 'Orti e arte' aims to preserve green areas between built areas to provide food for family consumption and add didactic, therapeutic, social and environmental value. |
|  | Description of actions | A complex of urban vegetable gardens (over 50,000m^2^) in areas destined to abandonment and degradation. 5 categories of gardens exist for (1) retirees and people with disabilities, (2) family units, (3) groups of families, (4) education of young people, (5) reintegration and therapeutic rehabilitation projects. |
|  | Role of Local Government | Initiation, funding and ongoing coordination |
|  | Targeted healthy and sustainable diet-related practice(s) | More plant-based foods  Eat seasonally  Connect with local food system  Minimise food waste |
|  | Targeted food system phase(s) | Agricultural production  Eating |
|  | Does the policy consider health? Y/N and how? | Sustainable diets and nutrition  Social and economic equity  Food production |
| Considerations  Evaluation | Does the policy consider equity? Y/N and how? | Not stated |
|  | Does the policy consider the broader food system? Y/N and how? | Y - promoting fruit and vegetable consumption |
|  | Has evaluation been planned for? | Y - allotment of plots for sub-groups of the population e.g. retirees, people with disabilities |
| Evaluation  Policy Development | Data collection tools, target population | Y - protection of biodiversity, reducing the food supply chain, increasing awareness of food value |
|  | Has the policy been effective? | Survey with 0-5 Likert scale, n = not stated |
|  | What evidence underpins the development of the policy? | Social benefits: Safe spaces in the city, exchange report, food security, know-how exchange, awareness of the food value, social inclusion, best quality of life. Economic benefits: Reproduction of landraces, short supply chain, reduce food budget, maintenance of public spaces, project of formation. Environmental benefits: Protection of bio-diversity, healthy safety, landscape improvement, maintenance of the rural culture, recycle organic matrixes. |
| Intervention Development  Citation | What type of evidence was used to develop the policy? | Not stated |
|  | What was the process for using evidence to develop the policy? | Not stated |
|  | Name of Policy | Not stated |

| Citation | Name of Policy | **Sustainable Food Procurement for Schools in Rome (2013)** |
| --- | --- | --- |
|  | Reference | x |
|  | Format and link or citation | [Case Study Document](https://ec.europa.eu/environment/gpp/pdf/news_alert/Issue14_Case_Study34_Rome_food.pdf) |
|  | Included study(s) cited within | 9, 11 |
| Geographic context | Region | Europe & Central Asia |
|  | Country | Italy |
|  | Signatory City | Catania |
|  | World Bank economic classification | High Income |
| Policy context | Is this intervention a policy itself or part of an over-arching policy? | Policy - regulatory |
|  | Timeframe for intervention | Tender process defined in the linked document: 2007 - 2012. The policy itself extends beyond this period. |
|  | Related policy interventions (Regional, National, State, Local) | All for Quality' program has been in place in Rome since 2001. 'Finance Law 488' is National legislation introduced in 1999 to introduce organic procurement in school canteens. In 2010, Rome's council introduced a green procurement policy for food and canteens. [Geographical Indications and Designations of Origin EU Legislation](https://eur-lex.europa.eu/LexUriServ/LexUriServ.do?uri=OJ:L:2007:189:0001:0023:EN:PDF) & [European Parliament resolution on Fair Trade and Development](https://eur-lex.europa.eu/LexUriServ/LexUriServ.do?uri=OJ:L:2007:189:0001:0023:EN:PDF) |
| Policy Aims & Activities | Policy aim | The objective behind Rome’s approach is to "support organic agriculture and organic food chains, ensure food safety and nutritional balance, and encourage good environmental performance of current and potential suppliers, through its school meal service" |
|  | Description of actions | This intervention describes the tender for school food service partners (preparation, cooking, serving, cleaning and waste management). There are key criteria for applicants to minimise environmental impact for example; non-food and food waste to be separated, single-use materials must be biodegradable and recyclable, food produced using organic agricultural practices, ban on genetically modified food for catering and animal feed, freshness for fruit and vegetable (e.g.. no more than 3 days between harvest and intake), meat freshness (delivered within 4 days of packaging), meat origin labelled (e.g. 'protected geographical indication' and 'protected denomination of origin'), seasonality based on Rome's climate to be used to plan menus and design recipes, meat served a maximum of twice per week. |
|  | Role of Local Government | Leadership/ownership. City of Rome writes the tender criteria for food service suppliers and therefore stipulates the technical requirements. |
|  | Targeted healthy and sustainable diet-related practice(s) | Variety (diversity)  Limit processed foods  Less animal-derived foods  More plant-based foods  Eat seasonally  Eat locally available foods  Minimise food waste  Support sustainable food production practices |
|  | Targeted food system phase(s) | Agricultural production  Distribution  Consumption  Waste |
| Considerations | Does the policy consider health? Y/N and how? | Not stated |
|  | Does the policy consider equity? Y/N and how? | Not stated |
|  | Does the policy consider the broader food system? Y/N and how? | Yes - promotion of organic agricultural practices, banning genetically modified food (for human and animal consumption), freshness criteria require a short and streamlined distribution system, seasonality |
| Evaluation | Has evaluation been planned for? | Yes - municipal dietitians carry out quality checks of the food daily to ensure that the terms of the contract are being respected. A contract was created by the City of Rome with two private laboratories which analyse 15 samples of food each day to monitor adherence to the policy. |
|  | Data collection tools, target population | The approach taken to evaluation (including tools) is not described in this policy document, nor in any of the included studies of this review. |
|  | Has the policy been effective? | The case study document outlines expected environmental outcomes: (i) by serving meat only twice per week, Roman schools will save 8,887 tonnes of CO2 equivalents each year, (ii) savings of water consumption (5,783m2 per year) associated with less meat consumption, (iii) savings of plastic waste estimated at 1,800 tonnes per year. |
| Policy Development | What evidence underpins the development of the policy? | European Green Procurement Policy (GPP) criteria and background report, Council Regulation on organic production and labelling of organic products: https://eur-lex.europa.eu/LexUriServ/LexUriServ.do?uri=OJ:L:2007:189:0001:0023:EN:PDF |
|  | What type of evidence was used to develop the policy? | Not stated |
|  | What was the process for using evidence to develop the policy? | J Smith et al (2016) describe the policy-making process for this intervention using a case study approach. They describe a range of political (political will to change, inclusive and incremental approach), environmental (National law (1999) created regulatory context that encouraged organic procurement at municipal level) and social (broader interpretation of 'quality' meals, innovative criteria aligned with socio-environmental outcomes, food education) forces that enabled implementation. |

| Citation | Name of Policy | **City of Turin: Guidelines on school canteen services (2015)** |
| --- | --- | --- |
|  | Reference | Xi |
|  | Format and link or citation | [Guidelines](https://sustainable-catering.eu/fileadmin/user_upload/enewsletter/Documents/Guidelines_ENG.pdf) - to be made regulatory |
|  | Included study(s) cited within | 23 |
| Geographic context | Region | Europe & Central Asia |
|  | Country | Italy |
|  | Signatory City | Turin |
|  | World Bank economic classification | High Income |
| Policy context | Is this intervention a policy itself or part of an over-arching policy? | Policy - regulatory. The evaluation results of the initial policy informed these guidelines which were to be adopted as an official political act and used in the 2018 procurement tender process. |
|  | Timeframe for intervention | 2015-2018 |
|  | Related policy interventions (Regional, National, State, Local) | Part of the [INNOCAT project](https://sustainable-catering.eu/actions/innocat-tenders/procurement-of-eco-innovative-school-catering-services/) that aimed to bring together public/private buyers to make tenders publicly available to promote eco-innovative catering, services and solutions. |
| Policy Aims & Activities | Policy aim | The City of Turin's school catering service provides food for 360 schools (nursery, primary and secondary levels), covering 50000 meals daily. The policy demonstrates Turin's commitment to sustainable procurement with specific indicators to objectively measure environmental achievement against the previous year. Two main criteria for the tender 2013-2016; i) more food sustainability and ii) more environmental sustainability. |
|  | Description of actions | The procurement policy includes several environmental requirements; provision of organic and/or fair trade fruit and vegetables, offer well-balanced menu with local and seasonal produce, promote new and equitable forms of dialogue with farmers, improve waste separation processes to increase composting potential, education activities to reduce waste and promote a healthy eating culture, specification of the procurement of meat (must be raised and butchered in the territory or neighbouring regions) provision of drinking water from the public water network (avoiding bottled water), use of reusable dishes, use of environmentally modes of transport for suppliers (e.g. natural gas-fuelled vehicles), purchase of school kitchen appliances based on energy efficiency/savings criteria, efficient handling of waste including redistribution of surplus food to people in need, ecological packaging for all food-related products (no single packaging, use of eco-labelled products, biodegradable packaging materials). To promote this work, the City of Turin attended the CompraVerde BuyGreen Forum in Rome in 2014, and the City of Turin facilitated a local event to bring public authorities interested in procuring sustainable catering services for schools together. To support the implementation of this policy, City of Turin partnered with Ecosistemi to offer a helpdesk for other local governments and business to increase cooperation and promote the importance and potential of sustainability. |
|  | Role of Local Government | Leadership/ownership. The City of Turin |
|  | Targeted healthy and sustainable diet-related practice(s) | Variety (diversity)  Avoid over-consumption  Limit processed foods  Less animal-derived foods  More plant-based foods  Eat seasonally  Eat locally available foods  Minimise food waste  Connect with local food system  Support sustainable food production practices  Promote safe tap water |
|  | Targeted food system phase(s) | Agricultural production  Distribution  Consumption  Waste |
| Considerations | Does the policy consider health? Y/N and how? | Y - many of the strategies are in place to offer a 'well-balanced' menu to students in order to meet their nutritional needs. Procurement specifications around the sourcing of locally raised and butchered meats are in place to assure 'high quality from the nutritional and organoleptic point of view' e.g. testing revealed that fat content is lower than average in local pig meat |
|  | Does the policy consider equity? Y/N and how? | Y - surplus food that is deemed suitable for human consumption is rescued and redistributed to feed people in need |
|  | Does the policy consider the broader food system? Y/N and how? | Y - procurement specifications favour sustainable agricultural production practices, strategies encourage low-carbon transportation methods for the distribution of food products, surplus food is returned to the food system via composting strategies |
| Evaluation | Has evaluation been planned for? | The case study on the website describes City of Turin's involvement in the INNOCAT project including defining measurable targets and the establishment of a partnership with the Chemical Laboratory of Turin Chamber of Commerce to monitor progress via carbon footprint analysis conducted by University of Turin. |
|  | Data collection tools, target population | Carbon footprint analysis |
|  | Has the policy been effective? | The results are not stated however the website advises that the results of the carbon footprint analysis will be used to identify areas to improve sustainability outcomes further. |
| Policy Development | What evidence underpins the development of the policy? | The guidelines have been developed based on the carbon footprint analysis of the initial 2013-2016 tender period, as conducted by the University of Turin. |
|  | What type of evidence was used to develop the policy? | The INNOCAT project is rooted within the Italian and European context whereby Green Public Procurement (GPP) have been strengthening over many years as part of the Europe 2020 strategy to support Public Procurement of Innovation. Evidence from these broader interventions was no doubt influential in designing the evaluation and implementation processes in Turin. |
|  | What was the process for using evidence to develop the policy? | In 2015, the results of the University of Turin's carbon footprint study were disseminated via a public event in Turin together with an opportunity to discuss available eco-innovation opportunities in the area with suppliers and other stakeholders. |

| Citation | Name of Policy | **Edible City Solution initiatives (2018)** |
| --- | --- | --- |
|  | Reference | Xii |
|  | Format and link or citation | [Website](https://www.arcgis.com/apps/MapJournal/index.html?appid=751e4f0954724a5fabea03620bf3d5b8) with case studies |
|  | Included study(s) cited within | 3, 22 |
| Geographic context | Region | Europe & Central Asia |
|  | Country | Netherlands |
|  | Signatory City | Rotterdam |
|  | World Bank economic classification | High Income |
| Policy context | Is this intervention a policy itself or part of an over-arching policy? | Part of an EU-wide Edible Cities Network |
|  | Timeframe for intervention | Not stated |
|  | Related policy interventions (Regional, National, State, Local) | Edible Cities Network https://www.edicitnet.com/ is a European demonstration program recognising the importance of urban agricultural initiatives for the city. The City of Rotterdam reports over 200 'green' initiatives and has a dedicated local government work package titled "SuperbFood - sustainable urban and peri-urban food provision" https://edepot.wur.nl/443120 |
| Policy Aims & Activities | Policy aim | The Edible Cities Network (EdiCitNet) aims to 'empower local communities to overcome social problems by their inclusive and participatory dynamics, and to create new green businesses and jobs, thereby generating local economic growth and fostering social cohesion'. |
|  | Description of actions | Rotterdam's living lab has a city-wide green initiatives network, facilitating green and edible initiatives and access to knowledge about local business opportunities and financing models. Over 200 nature-based Edible City Solution initiatives, mainly grass roots. |
|  | Role of Local Government | City of Rotterdam has main responsibility of administering the ECS activities. These are integrated in the top down program "Food & the City" (Gemeente Rotterdam, 2012) |
|  | Targeted healthy and sustainable diet-related practice(s) | More plant-based foods  Eat locally available foods  Eat seasonally  Connect with local food system  Support sustainable food production practices |
|  | Targeted food system phase(s) | Agricultural production  Distribution  Consumption |
| Considerations | Does the policy consider health? Y/N and how? | Y - increasing access to fruit and vegetables |
|  | Does the policy consider equity? Y/N and how? | Y - initiatives are targeting 'at risk' individuals and have an economic growth consideration, including to support employment outcomes |
|  | Does the policy consider the broader food system? Y/N and how? | Y - reducing the food supply chain, celebrating Dutch agricultural history and success |
| Evaluation | Has evaluation been planned for? | Y - Case studies as per referenced article |
|  | Data collection tools, target population | Questionnaire, SWOT Analysis. Population for questionnaire: City of Rotterdam contact person, n = not stated. |
|  | Has the policy been effective? | Rotterdam counts to date more than 200 nature-based Edible City Solution initiatives mainly grass-roots (Figure 4a). Most of ECS initiatives work with volunteers and often lack professional back-up and continuity in expertise transfer. All of them depend on (private) funds. When granted funds, they lack time, capacity and/or expertise to monitor and to show the funders the social benefits of their organizations. In addition, some initiatives may share the ambition of picking up commercial activities. Rotterdam aims at facilitating with the valorisation of their ECS. Here, the main challenge is to guide these fragmented and often vulnerable ECS to a stable network of ECS. Moreover, Rotterdam aims at the integration, employment and education in a socio-cultural diverse society and will overcome the fragile availability of expertise  and experience of the existing high potential ECS grass root movements. Thus, Rotterdam’s Living Lab will focus on an empowerment of ECS initiatives by fostering self-sustainment and on the development of a legal framework. |
| Policy Development | What evidence underpins the development of the policy? | Not Stated |
|  | What type of evidence was used to develop the policy? | Not Stated |
|  | What was the process for using evidence to develop the policy? | Not Stated |

| Citation | Name of Policy | **Seasonal food for kindergartens (2013)** |
| --- | --- | --- |
|  | Reference | Xiii |
|  | Format and link or citation | [Case Study Document](https://ec.europa.eu/environment/gpp/pdf/news_alert/Issue47_Case_Study99_Barcelona.pdf) |
|  | Included study(s) cited within | 11 |
| Geographic context | Region | Europe & Central Asia |
|  | Country | Spain |
|  | Signatory City | Barcelona |
|  | World Bank economic classification | High Income |
| Policy context | Is this intervention a policy itself or part of an over-arching policy? | Policy - regulatory |
|  | Timeframe for intervention | 2013-2015 |
|  | Related policy interventions (Regional, National, State, Local) | Barcelona + Sustainable City Council Programme, adopted in 1995. This led to an intensive participatory process to develop the 'Citizen Commitment to Sustainability' for 2002-2012, which was updated to continue for 2012-2022. Greening of Council Service (2001) led to the Green Office Guide and subsequently the "+ Sustainable City Council Programme in 2006 which aims to encompass all local authority action. Adoption of four governmental regulatory measures related to contracts and procurement. |
| Policy Aims & Activities | Policy aim | Increase the environmental sustainability of food services in kindergartens that are part of Barcelona City Council's Municipal Education (IMEB) Institute (n = 95). |
|  | Description of actions | IMEB introduced sustainability criteria into their catering tenders in 2006. The current intervention describes the tender process for 2013 which involved 49 kindergartens for 2 academic years. Criteria required bidders to (i) provide staff training about waste minimisation, waste collection, environmental characteristics of foods and sustainable cleaning procedures and products, (ii) have a waste management plan, (iii) provide all fresh vegetables - never frozen, (iv) fish must be fresh at least 3 times per month - can be frozen at other times, (v) fruit juices and yoghurts must be organic. A competition was facilitated to incentivise kindergartens to exceed the minimum criteria requirements. |
|  | Role of Local Government | Leadership/ownership. City of Barcelona writes the tender documentation and ultimately selects that successful bidders based on a point system linked to the sustainability criteria. |
|  | Targeted healthy and sustainable diet-related practice(s) | Variety (diversity)  Limit processed foods  More plant-based foods  Eat seasonally  Eat locally available foods  Minimise food waste  Support sustainable food production practices |
|  | Targeted food system phase(s) | Agricultural production  Consumption  Waste |
| Considerations | Does the policy consider health? Y/N and how? | Yes - "organic farming avoids the use of pesticides and herbicides on crops, which can pose a risk to human and eco-system health" |
|  | Does the policy consider equity? Y/N and how? | Yes - bidders with a higher percentage of employees with disabilities (above the 2% obliged by law) were given preference to win the contract. |
|  | Does the policy consider the broader food system? Y/N and how? | Yes - the case study document states their intention to move away from conventional farming systems (to protect biodiversity, soil fertility and system stability) by creating demand for organic agriculture and seasonal produce. |
| Evaluation | Has evaluation been planned for? | The case study document mentions monthly quality controls conducted by IMEB however indicates that in practice this is difficult to execute. School staff therefore conduct the evaluation as part of IMEB's quarterly review. Companies have to provide information about organic certification quarterly. On an ad-hoc basis, invoices for the purchase of organic products are required. It is stated that "monitoring measures should be strengthened to ensure compliance". |
|  | Data collection tools, target population | Invoices, quality reports |
|  | Has the policy been effective? | Not stated |
| Policy Development | What evidence underpins the development of the policy? | Not stated |
|  | What type of evidence was used to develop the policy? | Not stated |
|  | What was the process for using evidence to develop the policy? | Not stated |

| Citation | Name of Policy | **Barcelona green infrastructure and biodiversity plan (2020)** |
| --- | --- | --- |
|  | Reference | Xiv |
|  | Format and link or citation | [Plan for Action](https://ajuntament.barcelona.cat/ecologiaurbana/sites/default/files/Barcelona%20green%20infrastructure%20and%20biodiversity%20plan%202020.pdf) |
|  | Included study(s) cited within | 24 |
| Geographic context | Region | Europe & Central Asia |
|  | Country | Spain |
|  | Signatory City | Barcelona |
|  | World Bank economic classification | High Income |
| Policy context | Is this intervention a policy itself or part of an over-arching policy? | Action plan - no current regulatory components related to food |
|  | Timeframe for intervention | 2013-2020 |
|  | Related policy interventions (Regional, National, State, Local) | Related policy is the "The Municipal Plan of Urban Gaps with Territorial and Social Involvement ([BUITS Plan](https://ajuntament.barcelona.cat/ecologiaurbana/ca/pla-buits))" |
| Policy Aims & Activities | Policy aim | The overall plan aims to "achieve green infrastructure to act as a resource providing a wealth of services in a city where nature and the urbanity converge and enhance one another" |
|  | Description of actions | Whilst a small part of the overall plan, 12 urban vegetable gardens are mentioned as an example of a program with social and physical health value. Barcelona's urban vegetable gardening program was launched in 1994, and 546 users are registered, 205 of whom are at risk of social exclusion. School vegetable gardens are also mentioned. The plan acknowledges urban vegetable gardens as an environmental education activity, particularly for schools to connect children with agriculture and organic farming. The plan describes cross-generational benefits as the gardening education activities enable children to engage with the elderly. 4.8 of the plan aims to promote organic agriculture in urban and peri-urban areas. 9.7 of the plan includes creating accessible neighbourhood-run vegetable gardens. 10.7 of the plan describe the Barcelona City Council enhancing its environmentally friendly procurement policy, including food. |
|  | Role of Local Government | Leadership/ownership. The plan is much broader than food however describes some opportunities for Barcelona City Council to act on food-related activities. |
|  | Targeted healthy and sustainable diet-related practice(s) | Connect with local food system  Support sustainable food production practices |
|  | Targeted food system phase(s) | Agricultural production  Consumption |
| Considerations | Does the policy consider health? Y/N and how? | Y - the health, social and leisure activities offered to residents in city parks and gardens are considered. The green spaces allow for increased sports and recreational facilities, children’s' playgrounds |
|  | Does the policy consider equity? Y/N and how? | Y - Camps-Calvet et al (2016) concluded that the main beneficiaries of exosystem services from urban gardens are the elderly, low to middle income and migrant sub-groups of the population. |
|  | Does the policy consider the broader food system? Y/N and how? | Y - adoption of organic agricultural practices in urban gardens, educational gardens to teach school students about organic principles and food production practices. |
| Evaluation | Has evaluation been planned for? | The included paper in this study by Camps-Calvet et al (2016) describes an assessment of the contribution of urban gardens to the quality of their users. Measurable targets related to food are not included in the Action Plan. |
|  | Data collection tools, target population | Camps-Calvet et al (2016) conducted document analysis, interviews, surveys and observations to inform their assessment |
|  | Has the policy been effective? | Camps-Calvet et al (2016) identified two food-related services from urban gardens; in terms of provisioning services as a food supply and from a cultural services perspective, the quality of food. |
| Policy Development | What evidence underpins the development of the policy? | The plan states that indicators from existing strategies (Catalan, Spanish, European and internationally) will be used to inform the development of measurable targets. The plan outlines key documents to be used to guide implementation, including the City Biodiversity Index or the Singapore Index stemming from the Convention on Biological Diversity signed in Rio in 1992. |
|  | What type of evidence was used to develop the policy? | The plan mentions stakeholder consultation and high-level documentation as informing the development of this plan. |
|  | What was the process for using evidence to develop the policy? | The plan states that local technicians and representatives from political groups, institutions, companies and organisations took part in creating the Plan. |

| Citation | Name of Policy | **Sustainable Food Procurement Regulations in Zurich City (2010)** |
| --- | --- | --- |
|  | Reference | Xv |
|  | Format and link or citation | Further detail about these 2 regulations was not available in the grey literature (through online google search) therefore data has been extracted directly from the included study: Fesenfeld (2016) |
|  | Included study(s) cited within | 19 |
| Geographic context | Region | Europe & Central Asia |
|  | Country | Switzerland |
|  | Signatory City | Zurich |
|  | World Bank economic classification | High Income |
| Policy context | Is this intervention a policy itself or part of an over-arching policy? | 2 regulations as described in the paper |
|  | Timeframe for intervention | The two regulations were adopted in 2010 and 2014 however no further detail about their implementation timeframe was stated by Fesenfeld 2016 |
|  | Related policy interventions (Regional, National, State, Local) | 2014 [Case Study Report](http://supurbfood.eu/scripts/document.php?id=126) of SupurbFood (Sustainable Urban and Peri-urban Food Provision) project in Zurich as an overarching project, with two related regulatory policies as mentioned in the included study. This report describes the broader policy context, EU Food labelling and Country of Origin legislation, Zurich Agricultural Act, Zurich Waste Management Act, Zurich Water Conservation Act, Zurich Environmental Protection Act, Zurich Energy Act, Zurich Spatial Planning Act, Zurich Cultural Land Initiative, Quality Charter for Agriculture and Food Economy, |
| Policy Aims & Activities | Policy aim | The City of Zurich does not have a food strategy however various regulatory measures to improve the sustainability of the food system have been handed into the city parliament. According to the case study report, "Zurich has formulated the vision to develop towards a sustainable city-region by the year 2025". |
|  | Description of actions | These 2 regulations were adopted by the City of Zurich and affect food procurement across all city departments and canteens; (i) Increase procurement of fair-trade products and products in line with international labour standards (2010), (ii) Increase procurement of organic, seasonal and less energy-intensive products (2014). Other activities include; activities to maintain and support small-scale gardening and organic agriculture e.g. Transformation of the city green of Zurich into an “edible city”, whereby Zurich plants vegetables on public areas such as traffic islands. Sustainability article inserted into the local constitution via referendum. The Case study report also mentions this Council-driven regulatory measure however details of current implementation is not stated; “abandon use of meat produced in national and international factory farming in the municipal public canteens and to promote vegetarian menus". |
|  | Role of Local Government | Ownership. Fesenfeld (2016) describes that the 2 regulations were adopted by the City of Zurich after environmental and health departments put forward the proposal. These departments however held no authority to issue instructions to other departments (e.g. schooling and urban planning) therefore successful implementation was limited by authority/power higher up within council to make these required actions happen. |
|  | Targeted healthy and sustainable diet-related practice(s) | More plant-based foods  Eat seasonally  Support sustainable food production practices |
|  | Targeted food system phase(s) | Agricultural production  Consumption |
| Considerations | Does the policy consider health? Y/N and how? | Not stated |
|  | Does the policy consider equity? Y/N and how? | Not stated |
|  | Does the policy consider the broader food system? Y/N and how? | Yes - Focus is on increasing the purchase of organic, fair trade, seasonal food that is grown in desirable conditions in terms of international labour standards and less intensive farming methods. Increasing urban agriculture is aiming to shorten the food supply chain. |
| Evaluation | Has evaluation been planned for? | Fesenfeld (2016)'s interviewees describe the regulations as vague. If there are measurable targets they are not stated in this literature. Fesenfeld (2016) describes difficulties in measuring successful implementation of sustainable food procurement policies where public canteens/events/services relies on self-reporting surveys as the responsibility for collecting this data is dispersed across departments. |
|  | Data collection tools, target population | Fesenfeld (2016) conducted an in-depth process-tracing analysis of 13 food procurement regulations that had been officially adopted by City councils in Zurich, Munich and Nuremberg between 2003-2014. This study intended to compare the ambitiousness of policy targets and success of their implementation. Data collection involved data analysis of the regulation documents then semi-structured interviews (n=24) with decision-makers as identified from the document analysis. |
|  | Has the policy been effective? | Fesenfeld (2016) describes that Zurich served as the comparative, less successful case study location. Fesenfeld (2016) identified 5 policy design and process factors to determine the level of adoption and implementation deliberative and corporativist governance mechanisms (ii) high-level central coordination for cross-cutting policy implementation, (iii) policy networks to engage decision-makers, (iv) evidence-based instruments to inform policy design and implementation, (v) bundling short- and long-term benefits. These five factors were deemed to be more influential in Munich and Nuremberg than in Zurich. Interviewees describe Zurich's regulations as "vague", making implementation and monitoring challenging. Fesenfeld categorised the 2 regulations as level II adoption success as they didn't specify an exact percentage or exact criteria. Fesenfeld categorised the 2 Zurich regulations as II for implementation success meaning a "low degree of implementation". |
| Policy Development | What evidence underpins the development of the policy? | Work shared by other local governments through the established food policy networks. |
|  | What type of evidence was used to develop the policy? | Fesenfeld (2016) state that Zurich sourced expertise from other local government stakeholders also members of the 'Procura+ Campaign initiated by Local Governments for Sustainability (ICLEI). |
|  | What was the process for using evidence to develop the policy? | Fesenfeld (2016) described the process of engaging stakeholders and citizens in policy-making processes for both Munich and Nuremberg however made no mention of these processes for the case study of Zurich. |

| Citation | Name of Policy | **Bristol's Good Food Action Plan (2015)** |
| --- | --- | --- |
|  | Reference | Xvi |
|  | Format and link or citation | [Action Plan](https://bristolfoodpolicycouncil.org/wp-content/uploads/2016/02/food-action-plan-overview-19-2.pdf) |
|  | Included study(s) cited within | 6, 8, 13 |
| Geographic context | Region | Europe & Central Asia |
|  | Country | United Kingdom |
|  | Signatory City | Bristol |
|  | World Bank economic classification | High Income |
| Policy context | Is this intervention a policy itself or part of an over-arching policy? | Yes - part of the Good Food Plan for Bristol |
|  | Timeframe for intervention | 2015-2018 |
|  | Related policy interventions (Regional, National, State, Local) | In 2011 a Food Policy Council was formed by local government in Bristol. In 2012 the 'Good Food Charter' was produced as a "call to arms for all relevant stakeholders to adopt the principle of Good Food encompassing seven hallmarks; Good Food is not only tasty, healthy, affordable, but must also be produced and distributed in a way that it is good for nature, good for workers, good for animal welfare and good for local businesses". In 2013, the Good Food Plan for Bristol was launched. |
| Policy Aims & Activities | Policy aim | The Action Plan presents a shared plan of how Bristol will work towards achieving their vision "for a truly sustainable food city, renowned for the vibrancy and diversity of its food culture, for the high standards met by its thousands of food businesses, and for a food system, which, from field to fork, is good for people, places and the planet". The action plan exists to help coordinate, support and inspire the work of many players, and to make the system aims and actions more open and transparent. |
|  | Description of actions | The “Good Food Action Plan” identifies ten themes (p6) of actions. Bristol City Council – is supporting the good food work through its activities across numerous departments, including Public Health, Planning, Sustainability, Healthy Schools, Property, Economic Regeneration, Horticulture, Environmental Protection, Children’s and Young Peoples services, Neighbourhoods, Procurement and Contracting. BCC provides resources to support voluntary and community activities including Bristol Food Network, and is linking with neighbouring Councils to ensure consistency of approach. Examples of specific strategies; Connecting emergency food relief services with local producers to increase provision of fresh, healthy food, garden plots for people seeking asylum, pop-up fresh produce market stall by a local producer at the hospital to promote access to organic, seasonal, locally grown and fairly traded food, supporting Bristol schools to work towards and achieve the Mayor's Award for Excellence as a Health Improving School - including nutrition, health and gardening curriculum, adult masterclasses to reduce household food waste (e.g. "What's in your fridge" series), adding edible garden beds to train stations along the Severn Beach line (facilitated by local schools), University of Bristol's student union facilitating the 'Get Green' 2 year project to fund and develop student-led projects to promote flexitarianism and food waste reduction, pilot a national campaign titled 'Flexitarian City' which promotes a flexitarian diet in restaurants and communities throughout the city, Portland Centre of Integrative Medicine implementing the 'Kitchen on Prescription' program which involves motivational healthy eating cooking courses via social prescribing within mainstream healthcare, school and community garden projects, online local food store to enable stocking, packing and dispatch of local produce for home delivery, annual 'meet the buyer' events to connect purchasers with smaller, local suppliers, guides for sustainable food catering and menu planning (including reducing waste) for businesses, 'Fairtrade Fortnight' where a Fairtrade farmer visits a local school to encourage more schools, community groups and businesses to procure Fairtrade food, publish a Bristol citizen statement about the value of good quality soil to the city and how local government will demonstrate that value, plans to develop a Bristol Pollinator Strategy to promote better habitat management for insect pollinators required for food production, develop food distribution hub, expand and support CSA initiatives, support the Bristol Food Producers Network to scale-up local food production and collaboration amongst producers, 'Feed me and win' incentive program for residents using their food recycling bins, establishment of a kitchen onsite at Elm Tree Farm to use farm surpluses in preserves, chutneys, dehydrated and fermented foods to be sold direct to local restaurants, introducing wormeries into primary schools, research to identify where food waste is occurring across the city, 'Food Route' online platform connecting surplus food with businesses, 'FoodCycle' education program building capacity to reduce food waste and food insecurity in schools, the Sustainable Restaurant Association's 'FoodSave' project running food waste audits with small-medium businesses. |
|  | Role of Local Government | Bristol City Council provides resources to support voluntary and community activities including Bristol Food Network, and is linking with neighbouring Councils to ensure consistency of approach. The Council is a member of the Food Policy network and is identified as one of the lead organisations for a range of actions. |
|  | Targeted healthy and sustainable diet-related practice(s) | Eat locally available foods  Minimise food waste  Support sustainable food production practices  Prepare food at home with others  Connect with local food system |
|  | Targeted food system phase(s) | Agricultural production  Distribution  Retail  Consumption  Waste |
| Considerations | Does the policy consider health? Y/N and how? | Y - promoting healthy, affordable and sustainable food to the public, increase Bristol-grown fruit and vegetable procurement within the City |
|  | Does the policy consider equity? Y/N and how? | Y - increasing access to affordable good food, providing learning opportunities for growing and cooking, promote redistribution of food that would otherwise go to waste |
|  | Does the policy consider the broader food system? Y/N and how? | Y - open up markets for food grown by local, regional and Fairtrade producers, strengthen city links with local wholesale markets, abattoirs, dairies and farms, promote community-led fair-trade groups (e.g. CSA), minimise food waste |
| Evaluation | Has evaluation been planned for? | Yes. Bristol's Food Policy Council prepared a Baseline Report and this detailed action plan. Within the action plan, measurable outcomes are described, some which include plans for discrete evaluation. The action plan does not describe plans for an overall evaluation of the Good Food Plan. |
|  | Data collection tools, target population | Surveys and participation rates are mentioned in the Action Plan. Moragues-Faus & Morgan (2015) conducted case study research including interviews however these focused more on the policy-making process than efficacy. |
|  | Has the policy been effective? | None of the included studies in this scoping review reported on efficacy of the intervention. |
| Policy Development | What evidence underpins the development of the policy? | The baseline report "Who Feeds Bristol? Towards a resilient food plan" NHS Bristol and Bristol City Council is referenced as informing the development of Bristol's Good Food Action Plan <https://bristolfoodpolicycouncil.org/wp-content/uploads/2012/10/Who-Feeds-Bristol-report.pdf> |
|  | What type of evidence was used to develop the policy? | Expert opinion (an advisory group provided advice and guidance on the baseline report), evidence from benchmark cities, peer-reviewed literature and countrywide statistics |
|  | What was the process for using evidence to develop the policy? | The Bristol Food Network (2009) and the Bristol Food Policy Council (2011) included members from key sectors and facilitated the translation of evidence to influence policy-making. |

| Citation | Name of Policy | **Spade to spoon: digging deeper. A food strategy and action plan for Brighton and Hove (2012)** |
| --- | --- | --- |
|  | Reference | Xvii |
|  | Format and link or citation | [Action Plan](https://bhfood.org.uk/wp-content/uploads/2017/09/Spade-to-Spoon-report-interactive-PDF.pdf) |
|  | Included study(s) cited within | 8 |
| Geographic context | Region | Europe & Central Asia |
|  | Country | United Kingdom |
|  | Signatory City | Brighton and Hove |
|  | World Bank economic classification | High Income |
| Policy context | Is this intervention a policy itself or part of an over-arching policy? | Regulatory policy |
|  | Timeframe for intervention | 2012-2017 |
|  | Related policy interventions (Regional, National, State, Local) | This is the second local food strategy, this building upon success from the 2006 strategy. Evaluation of the 2006 strategy revealed that 90% of planned activities were implemented. Policies cited in the strategy include: 'Brighton & Hove City Council Domestic Waste Strategy (2009)', 'Best Food Forward, A One Planet Framework for Brighton & Hove, adopted by the City Sustainability Partnership (2011)', 'Climate Change Action Plan (2012)', 'NHS Brighton & Hove Public Health Directorate (2011)', 'National Childhood Measurement Programme (2010)' |
| Policy Aims & Activities | Policy aim | "Our vision is a city where everyone has the opportunity to eat fresh, healthy food from sustainable sources". The strategy describes 8 key outcomes and 9 aims with specific objectives for each. The 8 outcomes relate to diet, food poverty, community, economy, waste, climate change, natural resources and research and planning. |
|  | Description of actions | Education initiatives to promote H&S diets (training courses, working with schools, awareness campaigns), weight management services, cooking initiatives, offer food grants to promote food initiatives in deprived communities, develop sustainability criteria to the 'Healthy Choice Award' initiative (in all care homes, early years, cafes, restaurants), increase physical access to food by working with transport plans |
|  | Role of Local Government | Leadership/Ownership |
|  | Targeted healthy and sustainable diet-related practice(s) | Variety (diversity)  Avoid over-consumption  Limit processed foods  Less animal-derived foods  More plant-based foods  Eat seasonally  Eat locally available foods  Promote breastfeeding  Minimise food waste  Connect with local food system  Support sustainable food production practices  Prepare food at home with others |
|  | Targeted food system phase(s) | All |
| Considerations | Does the policy consider health? Y/N and how? | Y - the first Outcome states 'Reduce the number of people with diet related ill health' which is linked to Aims 1 and 2. E.g. Disrupting the year on year increase in childhood obesity. |
|  | Does the policy consider equity? Y/N and how? | Y - the second Outcome states 'Reduce the number of people living in food poverty' which is linked to Aim 2 and others where the local economy and employment opportunities are addressed. E.g. Reduce the life-expectancy difference between rich and poor areas of the city. |
|  | Does the policy consider the broader food system? Y/N and how? | Y - several outcomes consider the broader food system by 'engaging people in communal activities around food', 'creating a vibrant and sustainable food economy', reducing food waste and making waste a resource', respecting biodiversity and the limits of our natural resources. E.g. "The city and council are committed to a One Planet approach" |
| Evaluation | Has evaluation been planned for? | Yes. The final outcome relates to research and planning, to 'ensure that food is at the heart of planning and policy work'. There are 5 objectives to achieve this, involving a number of stakeholders (e.g. University) and approaches to evaluation (e.g. sourcing funding for research projects). |
|  | Data collection tools, target population | Appendix I tabulates sources of data available to measure the impact of this strategy. The table includes organisation, type of data and aim of the data for both National and Local policies. |
|  | Has the policy been effective? | The policy document does not describe the efficacy of the 2012-2017 intervention. It does however advise that 90% of the actions from the 2006 food strategy were achieved however no further detail is provided. The relevant included study from this scoping review (Sonnino, 2016) does not evaluate the efficacy either. |
| Policy Development | What evidence underpins the development of the policy? | The evidence cited within the 2012-2017 policy document includes local, national and international policy documents, scientific reports, public consultation, peer reviewed literature and statistics - national and local. |
|  | What type of evidence was used to develop the policy? | Brighton & Hove were the first city council in the UK to publish a citywide food strategy. Consultation and collaboration with key partners is described as part of the development process for the current 2012-2017 intervention. |
|  | What was the process for using evidence to develop the policy? | Brighton & Hove pride themselves in leading the way nationally and internationally with this intervention and describe working closely with global collaborators in the development of this intervention however specifics are not included in the policy document. |

| Citation | Name of Policy | **Flagship Food Boroughs in London (2014)** |
| --- | --- | --- |
|  | Reference | Xviii |
|  | Format and link or citation | [Website](https://www.london.gov.uk/what-we-do/business-and-economy/food/food-flagships/about-food-flagships) |
|  | Included study(s) cited within | 15 |
| Geographic context | Region | Europe & Central Asia |
|  | Country | United Kingdom |
|  | Signatory City | London |
|  | World Bank economic classification | High Income |
| Policy context | Is this intervention a policy itself or part of an over-arching policy? | Policy (Non-regulatory) |
|  | Timeframe for intervention | 2014 with no end date of funding stated. |
|  | Related policy interventions (Regional, National, State, Local) | [National School Food Plan 2013](http://www.schoolfoodplan.com/) which sets out 17 actions to transform what children eat in school and how they learn about food. Food Growing Schools, [Healthy Schools London](https://www.london.gov.uk/what-we-do/health/healthy-schools-london/awards/) and [London Healthy Workplace Charter](https://www.london.gov.uk/what-we-do/health/london-healthy-workplace-award/about-london-healthy-workplace-award). |
| Policy Aims & Activities | Policy aim | The Food Flagship program works with two boroughs (Lambeth and Croydon) who take part in new and existing food-related programs which are designed to: (i) improve the quality of food available to schools and communities, (ii) increase understanding of how diet effects health (iii) develop practical cookery skills, and (iv) encourage a love of good food. |
|  | Description of actions | Across the two involved boroughs, the following activities have been implemented; improving food-related curriculum (e.g. 'Maths with cookery' program brining practical meaning to numeracy skills, creating edible playgrounds), improving nutritional quality of food available at school and in the community (e.g. providing community grants to support growing and cooking healthy food, students are encouraged to eat school meals and only allowed to bring packed lunches under special circumstances to ensure students all have a hot, nutritious meal every day, incentivising healthy food choices with prizes for students, Gipsy Hill Village Hub providing opportunity for residents to be involved in food growing projects and learning food preparation skills, 'Natural Thinkers' program connecting children and parents with nature by networking Early Years practitioners with food co-ops, parks officers, local garden groups, etc, Workplace achievement program with nutrition actions), building capacity to grow and prepare food (e.g. cooking classes with students/parents/teachers, Schools investing in 'food laboratories' to enable students to learning cooking skills, bringing a local baker to teach students about breadmaking), improving access to nutritious emergency food relief (e.g. free breakfast clubs, 'Rose voucher scheme' identifies eligible families and provides weekly vouchers to spend at local fruit and vegetable markets). |
|  | Role of Local Government | The Food Flagships program is supported by the Mayor of London, City Hall and the London Food Board, with further support from major supermarkets. |
|  | Targeted healthy and sustainable diet-related practice(s) | Variety (diversity)  Eat seasonally  Eat locally available foods  Connect with local food system  Promote safe tap water  Prepare food at home with others |
|  | Targeted food system phase(s) | Consumption |
| Considerations | Does the policy consider health? Y/N and how? | Y - majority of activities are focused on improving the nutritional quality of program participants (students, parents, teachers, community members). E.g. 'Eat Well Croydon' is a voluntary scheme which had twenty local businesses sign up, showing their commitment to improve food choices, preparation and cooking methods to reduce saturated fat and salt in food available for consumption outside the home. |
|  | Does the policy consider equity? Y/N and how? | Y - acknowledges that people experiencing food insecurity are less likely to eat a healthy diet. Activities involve improving the nutritional quality of emergency food relief options. Croydon borough partnered with 'Good Food Matters', a charity dedicated to helping people of all abilities, ages and backgrounds to build healthier lifestyles by hosting food growing lessons and cooking classes. |
|  | Does the policy consider the broader food system? Y/N and how? | Y - however it's not the focus. By building capacity to grow food, this intervention is promoting local, seasonal food and shorter supply chains. Croydon's 'Healthy Business Project' was led by the Council's Economic Development team and offered free training to local food businesses to improve the health and sustainability of their businesses through financial planning, sourcing local produce, managing food production and marketing skills. |
| Evaluation | Has evaluation been planned for? | Schools must complete an audit every 2 years to demonstrate their achievements, before receiving an award as part of the London Healthy Schools Programme. |
|  | Data collection tools, target population | The website nor the included study include details of the data collection tools or approach to evaluation. |
|  | Has the policy been effective? | The website states that although this intervention is focussed on two boroughs, the whole of London has benefited from taking a 'whole environment approach' to food. No specific evaluation results are included in the website or included study. |
| Policy Development | What evidence underpins the development of the policy? | This intervention is based on National frameworks and projects and therefore is guided by best-practice within the local government area and beyond. No specific documents are referenced on the website or in the included study. |
|  | What type of evidence was used to develop the policy? | Not stated on the website or in the included study. |
|  | What was the process for using evidence to develop the policy? | Not stated on the website or in the included study. |

| Citation | Name of Policy | **The London Food Strategy (2018)** |
| --- | --- | --- |
|  | Reference | Xix |
|  | Format and link or citation | [Strategy](https://www.london.gov.uk/sites/default/files/final_london_food_strategy.pdf) & [Implementation Plan](https://www.london.gov.uk/sites/default/files/implementation_plan_2018-2023.pdf) to support the strategy document: |
|  | Included study(s) cited within | 8, 22 |
| Geographic context | Region | Europe & Central Asia |
|  | Country | United Kingdom |
|  | Signatory City | London |
|  | World Bank economic classification | High Income |
| Policy context | Is this intervention a policy itself or part of an over-arching policy? | Regulatory policy |
|  | Timeframe for intervention | 2018 - 2023 |
|  | Related policy interventions (Regional, National, State, Local) | This policy document clearly states the relevant Mayoral strategies which are linked to the Food Strategy: Healthy and Sustainable Food for London: the mayor's food strategy (2008), New London Plan, the London Health Inequalities Strategy, the Mayor’s Economic Development Strategy, the London Environment Strategy, the Mayor’s Transport Strategy, Culture for All Londoners Strategy, the Mayor’s Skills for Londoners Strategy, the Mayor’s Vision for a Diverse and Inclusive City, a Tourism Vision for London, and a Vision for London as a 24-Hour City |
| Policy Aims & Activities | Policy aim | The London Food Strategy defines the Mayor's priorities for improving London's food system. The vision is for "every Londoner to have access to healthy, affordable food and sets a direction of travel for the Mayor to work collaboratively with partners to achieve this ambition". |
|  | Description of actions | The London Food Strategy is structured into six key settings, each with several actions. (i) Good Food at Home, and Reducing Food Insecurity e.g. Reduce consumption of meat, campaign for employers to pay the London Living Wage. (ii) Good Food Economy, Shopping and Eating Out e.g. support farmers' markets. (iii) Good Food in Community Settings and Public Institutions e.g. Look for food that's accredited by Fairtrade, Sustainable Fish Cities, Good Farm Animal Welfare. (iv) Good Food for Pregnancy and Childhood e.g. Encourage childcare centres to work towards Healthy Early Years London Award. (v) Good Food Growing, Community Gardening and Urban Farming e.g. support urban farms by buying from box schemes, link schools with Food Growing Schools London scheme. (vi) Good Food for the Environment e.g. reduce edible food waste via Love Food Hate Waste campaign, buy and eat sustainably and locally produced food. |
|  | Role of Local Government | Leadership/Ownership. The London Food Strategy was developed by the Greater London Authority on behalf of the Mayor of London, and in partnership with the London Food Board. The actions are itemised based on what the Mayor will do to deliver change. |
|  | Targeted healthy and sustainable diet-related practice(s) | All |
|  | Targeted food system phase(s) | Agricultural production  Distribution  Retail  Consumption  Waste |
| Considerations | Does the policy consider health? Y/N and how? | Y - promoting healthy food choices through modifications to the food environment in a number of settings (public institutions, childcare, schools) and menu and product labelling in the retail setting |
|  | Does the policy consider equity? Y/N and how? | Y - the first setting promotes an increase in household food security, several actions prompt Londoners to advocate for employers to pay the London Living Wage |
|  | Does the policy consider the broader food system? Y/N and how? | Y - actions promote sustainable food production practices, localised distribution and retail systems, and waste minimisation |
| Evaluation | Has evaluation been planned for? | Yes. An indicators framework is included on pages 2-4 of the implementation plan document, with a detailed description included in Appendix A which includes the data source owner and timeframe for each indicator. |
|  | Data collection tools, target population | The London Food Strategy Indicator Framework (Appendix A of the Implementation Plan) describes a range of data sources: surveys (e.g. breastfeeding prevalence at 6-8 weeks postpartum), estimates (e.g. food-related GHG emissions) and administrative (e.g. prevalence of overweight and obesity in state schools). |
|  | Has the policy been effective? | The intervention timeline is 2018-2023 and the strategic document or implementation plan do not describe efficacy results. |
| Policy Development | What evidence underpins the development of the policy? | The strategy was written by the Greater London Authority and the London Food Board, based on best-practice evidence. Consultation on drafts included policy-makers from New York City and Toronto, together with almost 150 organisations and thousands of Londoners during the consultation period. |
|  | What type of evidence was used to develop the policy? | Expert opinion (London Food Board), evidence and input from benchmark cities, peer-reviewed literature and countrywide statistics |
|  | What was the process for using evidence to develop the policy? | Not stated |

| Citation | Name of Policy | **National School Feeding Program of Brazil - Sustainable Schools initiative (2009)** |
| --- | --- | --- |
|  | Reference | xx |
|  | Format and link or citation | [Website](http://www.fao.org/in-action/program-brazil-fao/projects/school-feeding/en/) |
|  | Included study(s) cited within | 2, 10, 17 |
| Geographic context | Region | Latin America and Caribbean |
|  | Country | Brazil |
|  | Signatory City | Araraquara, Belo Horizonte, Curitiba, Porto Alegre, Praia, Rio de Janeiro, Sao Paulo |
|  | World Bank economic classification | Upper Middle Income |
| Policy context | Is this intervention a policy itself or part of an over-arching policy? | A federal policy, implemented at a local government level. |
|  | Timeframe for intervention | School feeding program commenced 1940's, family farming policy introduced 2009 with no end date stated. |
|  | Related policy interventions (Regional, National, State, Local) | Brazil-FAO International Cooperation was signed in 2008 between the Federal Government of Brazil and the FAO. Aims to promote formulation and implementation of sustainable school feeding programs ('Sustainable Schools' initiative) based on the experience in Brazil. In 2009 the Family Farming Law (Law n. 11,947/2009) was accepted which requires at least 30% of funds granted by Brazilian government be used to buy food directly from family farmers. |
| Policy Aims & Activities | Policy aim | The Sustainable Schools initiative aims to provide quality food, from family farming, to public based education, philanthropic, community and youth and adult schools, as a strategy for improving school feeding, strengthening family farming and of local development. |
|  | Description of actions | 1955 - mandatory school feeding in Brazilian primary and secondary schools. 2009 - introduction of family farming policy where 30% minimum finances are spent on procurement of organic agricultural products from local farmers. Current activities now extend beyond food provision to include food and nutrition education in the school curriculum in a cross-cutting manner, school menus are developed by a nutritionist to ensure local culture and eating traditions are prioritised and that the region's sustainability and agricultural diversity are considered. |
|  | Role of Local Government | Funded Federally, implemented at Municipality level. |
|  | Targeted healthy and sustainable diet-related practice(s) | Variety (diversity)  Limit processed foods  Eat seasonally  Eat locally available foods  Connect with local food system  Support sustainable food production practices |
|  | Targeted food system phase(s) | Agricultural production  Consumption  Distribution |
| Considerations | Does the policy consider health? Y/N and how? | Y - increasing intake of healthy food for school-aged children |
|  | Does the policy consider equity? Y/N and how? | Y - acknowledges this intervention as one of many strategies to address food insecurity in Brazil. The School Feeding Program can be considered a "cross-cutting policy in addressing poverty and other social issues, because it not only encourages the human development of students, improves eating habits, and ensures access to safe and adequate food; it also promotes the development of the local economy by purchasing food produced in the school's environment." |
|  | Does the policy consider the broader food system? Y/N and how? | Y - the 2009 iteration strengthens the local food system by enforcing 30% local food procurement |
| Evaluation | Has evaluation been planned for? | Yes - there are multiple evaluation studies published in the peer-reviewed literature. Of the included studies for this scoping review, Goncalves et al (2015) evaluate the quality of menus before and after the 2009 law was passed. |
|  | Data collection tools, target population | Goncalves et al (2015) conduct semi-structured questionnaires and menu assessment to describe changes to the school menus after the introduction of the Sustainable Schools initiative. 82 cities participated. |
|  | Has the policy been effective? | Goncalves et al (2015) conclude that 74.1% of the cities studied actually purchase food from family farms with the lack of interest from farmers and sanitary inadequacy being reported as barriers to implement the 30% procurement policy. The quality of menus has improved and nutritionists have declared that 69% of cities studied are interested in increasing fruit, legume and vegetable procurement. |
| Policy Development | What evidence underpins the development of the policy? | Scwartzman et al (2017) describe the history to the family farming policy. Brazil's Zero Hunger Strategy, Brazilian Food Acquisition Program (PAA), Human Right to Adequate Food, Sustainable Development all underpin the policy development. |
|  | What type of evidence was used to develop the policy? | Scwartzman et al (2017) interviewed actors involved in drafting the family farming legislation. Social movements and family farmers advocated for legislative change at the same time that civil society and health, nutrition professionals were discussing the issues of food security and sustainable development. |
|  | What was the process for using evidence to develop the policy? | Scwartzman et al (2017) pages 4-6 |

| Citation | Name of Policy | **Belo Horizonte food security policy (2011)** |
| --- | --- | --- |
|  | Reference | Xxi |
|  | Format and link or citation | [Website](https://www.futurepolicy.org/food-and-water/belo-horizontes-food-security-policy/) |
|  | Included study(s) cited within | 15 |
| Geographic context | Region | Latin America and Caribbean |
|  | Country | Brazil |
|  | Signatory City | Belo Horizonte |
|  | World Bank economic classification | Upper Middle Income |
| Policy context | Is this intervention a policy itself or part of an over-arching policy? | Policy - regulatory |
|  | Timeframe for intervention | Ongoing policy, specific start and finish dates are not stated |
|  | Related policy interventions (Regional, National, State, Local) | Belo Horizonte Municipal Law No. 6.352, 15/7/1993 - established a historical milestone by creating the 'Municipal Secretariat of Supply' (organisational framework committed to food sovereignty). Municipal Law 10,255 of 13/9/2011 which establishes policy to support urban agriculture as part of the food security policy. . |
| Policy Aims & Activities | Policy aim | SUSAN (Under Secretariat for Food and Nutrition Security) coordinates the intervention and believes that 'the right to adequate, healthy, sufficient and nutritious food is an inherent right of citizenship and is the duty of the public sector to ensure food and nutrition security.' |
|  | Description of actions | Three fundamental pillars; (i) the direct supply of food to the population through popular restaurants, school meals, food assistance to social assistance organizations, and the food bank; (ii) market regulation and promotion of fairs to provide low-cost food to the population e.g. vegetable markets, farm-direct organic fairs; (iii) strengthening family and urban agriculture e.g. institutional and community agroecological systems, establishment of productive backyards, community gardens in vulnerable communities, institutional procurement of family farming. 20 interconnected programmes. Transversal to the 3 fundamental pillars are initiatives for food and nutrition education, and training in agroecology and gastronomy. |
|  | Role of Local Government | Leadership/ownership. The mayor (Patrus Ananias) created a Secretariat for Food Policy and Supply - with a 20-member council representing municipal, state and federal government, food producers and distributors, consumer groups, research institutions, churches, civil society, labour unions, etc. The Mayor (a member of the working party) dedicated US$18m in the initial annual budget and continued unwavering support throughout his 5-year term. |
|  | Targeted healthy and sustainable diet-related practice(s) | Variety (diversity)  Minimise food waste  Connect with local food system  Support sustainable food production practices |
|  | Targeted food system phase(s) | All |
| Considerations | Does the policy consider health? Y/N and how? | Y - 'stimulate healthy eating habits and lifestyle habits', increasing availability of healthy foods for all people, regardless of economic status |
|  | Does the policy consider equity? Y/N and how? | Y - the explicit mandate of the Secretariat for Food Policy and Supply was to increase access to healthy food for all as a measure of social justice |
|  | Does the policy consider the broader food system? Y/N and how? | Y - urban and family farming is considered central to the achievement of a human right to adequate food |
| Evaluation | Has evaluation been planned for? | Not stated on the webpage reviewed. |
|  | Data collection tools, target population | Not stated on the webpage reviewed. |
|  | Has the policy been effective? | Included study (IPES, 2017) highlights the success of this policy; enduring 25 years with core principles intact, sustaining changes in political leadership, institutionalisation of the right to food at a federal level, driving supportive national policy. The state of Minas Gerais (which Belo Horizonte is a part of) has since adopted their [food security and nutrition policy](https://www.almg.gov.br/acompanhe/noticias/arquivos/2018/01/02_sancao_politica_seguranca_alimentar.html) in 2018. The success of Belo Horizonte's food security policy has since spread to [Windhoek, Namibia](https://www.worldfuturecouncil.org/wp-content/uploads/2017/03/Food-Handbook-final-web.pdf) (Africa) |
| Policy Development | What evidence underpins the development of the policy? | Not stated on the webpage reviewed. IPES details the expertise of multi-disciplinary working party members, including community representation, who contributed to the development. |
|  | What type of evidence was used to develop the policy? | Not stated specifically however this policy is based on evidence from 25 years of regulatory policy action in the local government area. |
|  | What was the process for using evidence to develop the policy? | The webpage states that 'a multi-stakeholder, participatory and multi-level approach' was applied to the formulation of Belo Horizonte's food security policy. IPES (2017) describes the bottom-up pressure from the Citizens' Action Campaign Against Hunger and Food Life. |

| Citation | Name of Policy | **Public Policy on Food Security, Food Sovereignty and Nutrition (2005)** |
| --- | --- | --- |
|  | Reference | xxii |
|  | Format and link or citation | FAO Case Study [summary document](http://www.fao.org/3/CA0652EN/ca0652en.pdf) |
|  | Included study(s) cited within | 15, 20 |
| Geographic context | Region | Latin America and Caribbean |
|  | Country | Colombia |
|  | Signatory City | Medellin |
|  | World Bank economic classification | Upper Middle Income |
| Policy context | Is this intervention a policy itself or part of an over-arching policy? | Policy - regulatory |
|  | Timeframe for intervention | Established 2005. No end date stated. |
|  | Related policy interventions (Regional, National, State, Local) | The City of Medellin has had a public policy for food and nutrition security since 2005. This is part of the City Region Food System of Medellin (includes 31 municipalities). Plan for Food and Nutrition Security (SAN) for the period 2016-2028, aiming to ensure in the next 12 years a hunger-free and food-sovereign city. |
| Policy Aims & Activities | Policy aim | IPES 2017 states that this policy aims to "provide an adequate, balanced, healthy diet for all, in the context of high food insecurity where half the population is under- or overweight." |
|  | Description of actions | This policy includes various actions. Implementation of vegetable gardens for the production of local fruits and vegetables. Public procurement to favour local food producers. Promotion of the culture of 'self-consumption' within families - generating and selling surplus food, and promoting other mechanisms of food access other than retail purchasing. E.g. "Medellin Solidaria"; communitarian restaurants for elderly; family and community gardens; research on food practices and ancestral knowledge; gastronomic workshops and education on healthy habits in schools. |
|  | Role of Local Government | The Council of Medellin created this public policy in 2005, and the 'Food Security Unit' which manages it. This committee is chaired by the Mayor and involves representation from city departments, NGOs, academia, business and the health sector. |
|  | Targeted healthy and sustainable diet-related practice(s) | Variety (diversity)  More plant-based foods  Eat seasonally  Eat locally available foods  Connect with local food system  Support sustainable food production practices |
|  | Targeted food system phase(s) | Agricultural production  Distribution  Consumption |
| Considerations | Does the policy consider health? Y/N and how? | Y - increasing healthy eating habits in the school population |
|  | Does the policy consider equity? Y/N and how? | Y - activities target vulnerable families, children and the elderly e.g. Medellin Solidaria program |
|  | Does the policy consider the broader food system? Y/N and how? | Y - by strengthening urban and per-urban agricultural processes |
| Evaluation | Has evaluation been planned for? | Yes. The outcomes of this policy are monitored by the University of Antioquia. Research is focused on 'investigating food-related processes and models from food practices, ancestral knowledge in food, nutritional and food security status, biophysical conditions of the population, food habits, and environmental awareness, among others. These projects also generate timely analysis from various disciplines, recommendations and new research for work proposals.' |
|  | Data collection tools, target population | Details of evaluation tools or approaches to the evaluation are not included in the cited studies or linked cased study summary. |
|  | Has the policy been effective? | The case study reveals key outcomes; 476 educational establishments involved attended daily by 293000 children (aiming to improve academic performance, school attendance and establish healthy eating habits), provision of 25-55% of food served in 20 dining halls ('Comedores Comunitarios') serving 2,0000 elderly participants, establishment of 1007 rural family gardens, urban terraces, associative gardens and institutional gardens. |
| Policy Development | What evidence underpins the development of the policy? | Population growth, increasing food insecurity rates, and 50% of Medellin's population is either under- or overweight (IPES, 2017). Learnings from policy action since first created in 2005. |
|  | What type of evidence was used to develop the policy? | Dubbeling et al (2017) describe insights from assessing and planning Medellin and Quito's city region food systems (CRFS) and describes future work to support regional value chains linking regional producers with local demand, and improving access and availability of safe and diverse food for city dwellers by strengthening food supply and distribution systems. |
|  | What was the process for using evidence to develop the policy? | Not stated |

| Citation | Name of Policy | **Participatory Urban Agriculture Project (AGRUPAR) (2000)** |
| --- | --- | --- |
|  | Reference | xxiii |
|  | Format and link or citation | [Website](https://www.futurepolicy.org/global/quito-agrupar/) ('FuturePolicy.org' - an online database of sustainable policy solutions, World Future Council) |
|  | Included study(s) cited within | 15, 20 |
| Geographic context | Region | Latin America and Caribbean |
|  | Country | Ecuador |
|  | Signatory City | Quito |
|  | World Bank economic classification | Upper Middle Income |
| Policy context | Is this intervention a policy itself or part of an over-arching policy? | Policy - regulatory |
|  | Timeframe for intervention | Started in 2000, no end date stated as it's still running according to the website (2016) and Dubbeling et al (2017) |
|  | Related policy interventions (Regional, National, State, Local) | Constitution of Ecuador of 2008, Organic Agriculture Law of 2007, Food Sovereignty Law of 2009, National Plan for Good Living of 2013, Plan of Development for the Metropolitan District of Quito of 2015, Quito’s Resilience Strategy, Quito’s Climate Action Plan, Municipal ordinance No. 084 on Social Responsibility, Municipal ordinance No. 048 on Urban Fauna. In 2012, a Resolution conferred more formality to the execution of AGRUPAR within CONQUITO, meaning that urban agriculture in Quito is now institutionalized as a permanent service. |
| Policy Aims & Activities | Policy aim | Improve the life of the most vulnerable groups - foremost women, elderly and persons with disabilities, but also unemployed, refugees, migrants and Indigenous people - through agricultural activities to advance food security and food sovereignty, improved incomes, job creation, environmental management, gender equity, social inclusion and the generation of productive activities. |
|  | Description of actions | The programme has 4 key areas of action; (i) Support for urban, community and institutional gardening for home consumption and the sale of leftovers. E.g. training in organic production, nutrition, food processing and marketing, vertical gardening. Establishment of community gardens on communal land. (ii) Support for market-oriented local production in the DMQ region. E.g. Once household food security is achieved, this program encourages micro-enterprises (with start-up grants available) to sell fruit, veg and processed products such as jams, cheese, yoghurt, etc (iii) Food supply and distribution. E.g. food sold in markets in low-income neighbourhoods and peri-urban zones, improved processing technologies (containers, packaging, labels), connect organic farmers with local hotels and restaurants, AGRUPAR shares cost of organic certification with participating producers (iv) Promotion of food consumption, healthy diets and nutrition through bio-fairs and education. E.g. training, technical advice and logistics. Provides seeds, inputs, equipment and supply of animals to producers. |
|  | Role of Local Government | In 2000, Local Government representatives from Latin America and the Caribbean met, the outcome being the landmark 'Quito Declaration', the first call for cities to "embrace urban agriculture". City of Quito representatives created the policy and oversee its' implementation, engaging representatives from other sectors also (farmers, NGOs, academia and students). 2018 budget was USD 283,336, mainly financed by City of Quito however participating farmers contribute infrastructure (e.g. greenhouses, small sheds for animal husbandry) and training participants pay a small fee, unless part of an 'at risk' group. |
|  | Targeted healthy and sustainable diet-related practice(s) | More plant-based foods  Eat seasonally  Eat locally available foods  Connect with local food system  Support sustainable food production practices  Minimise food waste |
|  | Targeted food system phase(s) | All |
| Considerations | Does the policy consider health? Y/N and how? | Y - promotion of health diets to ultimately improve quality of life, increasing nutrition capacity through training |
|  | Does the policy consider equity? Y/N and how? | Y - focuses on vulnerable groups (women, elderly, etc), start-up grants available for producers without adequate start-up capital, participation fee for training is waved if part of an 'at risk' population group, low-income neighbourhoods are prioritised for organic markets |
|  | Does the policy consider the broader food system? Y/N and how? | Y - the website states "The Programme works along the entire food chain and focuses on producing, processing and distributing healthy food from urban and peri-urban gardens. It provides technical assistance and capacity-building; creation of production infrastructures and livestock improvement; management of micro-entrepreneurship; marketing and promotion; access to microcredit; and applied research to facilitate agroecology." |
| Evaluation | Has evaluation been planned for? | Yes - academic partners are part of the implementation committee. The website doesn’t include measurable targets. |
|  | Data collection tools, target population | Data collection tools nor evaluation approaches are described on the website or in the included studies within this review (IPES, 2017) |
|  | Has the policy been effective? | From the website: "In its 16 years of existence, AGRUPAR has continuously expanded and considerably advanced food security, job creation, income generation, environmental management, gender equity, social inclusion of vulnerable groups such as women, elderly and migrants, and micro-entrepreneurship. Due to its impressive socioeconomic and environmental impact, its participatory and holistic approach involving the most vulnerable groups, and its full respect for the Future Justice Principles and Elements of Agroecology, AGRUPAR was recognized with the Future Policy Silver Award 2018, awarded by the World Future Council in partnership with FAO and IFOAM – Organics International." |
| Policy Development | What evidence underpins the development of the policy? | The long history of urban agricultural policy, and deep-rooted partnerships (2000 Quito Declaration) have grounded this policy in evidence. |
|  | What type of evidence was used to develop the policy? | Dubbeling et al (2017) describe insights from assessing and planning Medellin and Quito's city region food systems (CRFS) and describes Quito's challenges in bringing together local, provincial and national level stakeholders given political differences. Bringing together urban and peri-urban agricultural programs, provincial and national rural agriculture and working across jurisdictional boundaries is essential. |
|  | What was the process for using evidence to develop the policy? | The website states "AGRUPAR was developed on the basis of a broad, largely women-led community consultation. It explicitly recognizes the role of urban agriculture for wider social, ecological and economic development and works along the entire food chain." |

| Citation | Name of Policy | **Toronto Food Strategy (2010)** |
| --- | --- | --- |
|  | Reference | xxiv |
|  | Format and link or citation | [Strategic Report](https://www.toronto.ca/legdocs/mmis/2018/hl/bgrd/backgroundfile-118079.pdf) |
|  | Included study(s) cited within | 5, 8, 14, 15, 22 |
| Geographic context | Region | North America |
|  | Country | Canada |
|  | Signatory City | Toronto |
|  | World Bank economic classification | High Income |
| Policy context | Is this intervention a policy itself or part of an over-arching policy? | Yes - Adopted by the [Board of Health on 1 June, 2010](http://app.toronto.ca/tmmis/viewAgendaItemHistory.do?item=2010.HL31.1) http://app.toronto.ca/tmmis/viewAgendaItemHistory.do?item=2010.HL31.1 |
|  | Timeframe for intervention | Toronto Food Strategy 2010 - present |
|  | Related policy interventions (Regional, National, State, Local) | Provincial 'Local Food Act 2013' which aims to 'foster successful and resilient local food economies and systems throughout Ontario'. Toronto Food Policy Council (formed 1990), Toronto Food Charter (2001), "Cultivating Food Connections: Toward a Healthy and Sustainable Food System for Toronto" (2010), GrowTO: An Urban Agriculture Action Plan for Toronto (2012), Toronto Agriculture Program (2013), Toronto Food Strategy has ongoing amendments to align with Toronto Public Health Plans |
| Policy Aims & Activities | Policy aim | The Toronto Food Strategy (TFS) aims to move the needle towards a food system that positively impacts human and environmental health. This kind of transformative change requires a long-term effort by global, regional and local stakeholders – community, institutional, private sector, government, producers, and academia |
|  | Description of actions | Activities within the City of Toronto's Food Strategy are categorised into (i) food and health, (ii) food and community, (iii) food and housing, (iv)food and jobs, (v) food and poverty, (vi) food and land, (vii) food as a public good, (viii) food and environment. The Toronto Food Strategy Action Work Streams align with the pillars of the MUFPP and provides examples of progress for each: governance (Toronto Food Policy Council, Mayor proclaimed an Urban Agriculture Day which has since been extended to a week to highlight innovative UA initiatives in the city including a tour for City Councillors, media and City staff to meet UA participants), health (sustainable diets and nutrition), social and economic equity (Community Food Works is an initiative for low-income residents and refugees providing food handler training, nutrition education and employment support), food production, supply and distribution (FoodReach is an online portal for local organisations including emergency food relief agencies to order fresh and healthy foods at wholesale prices for free next-day delivery), and food system waste. |
|  | Role of Local Government | Development and implementation leadership |
|  | Targeted healthy and sustainable diet-related practice(s) | All |
|  | Targeted food system phase(s) | All |
| Considerations | Does the policy consider health? Y/N and how? | Y - an extensive range of initiatives under the 'food and health' domain e.g. SSB Recommendations, Food Vulnerability Assessment, Spot Marketing to Kids, Healthiest Babies Possible, Prenatal programs, Breastfeeding Clinics, Healthy Menu Choices Act Inspections, Food Recall Response, Sip Smart, Nutritious Food Basket |
|  | Does the policy consider equity? Y/N and how? | Y - equity-based food security, emergency food security, considers the results of a vulnerability assessment, dedicated activities under 'food and poverty' domain e.g. Toronto Strong Neighbourhood Strategy 2020, Residential Apartment Commercial Zoning, Tower Renewal |
|  | Does the policy consider the broader food system? Y/N and how? | Y - initiatives spanning the food system are considered across the variety of policy domains. E.g. Ontario Food Collaborative, Food Waste Audits, Community and Allotment Gardens, Pollinators Strategy, Energy Efficiency Workshops for Food Processors, Urban Food Policy Project, Institutional Buying of local foods, Toronto Agricultural Program |
| Evaluation | Has evaluation been planned for? | Y - Toronto's Food Systems Monitoring Framework |
|  | Data collection tools, target population | TSF worked with MUFPP (FAO), the C40 Network and the Netherlands-based RUAF (Resource Centres on Urban Agriculture and Food Security) to develop the Toronto Food Strategy Indicator framework |
|  | Has the policy been effective? | y - outcomes from each initiative are detailed in the 2018 report |
| Policy Development | What evidence underpins the development of the policy? | Internal and external (local, national and international) policy documents and scientific reports. Public consultation, peer reviewed literature, statistics - national and local. |
|  | What type of evidence was used to develop the policy? | Public consultation, peer review literature, statistics - national and local |
|  | What was the process for using evidence to develop the policy? | For background information and to persuade council to support the intervention |

| Citation | Name of Policy | **Toronto Golden Horseshoe Food and Farming Action Plan (2012)** |
| --- | --- | --- |
|  | Reference | xxv |
|  | Format and link or citation | [Action Plan](https://www.toronto.ca/wp-content/uploads/2017/10/88b2-foodfarming_actionplan.pdf) |
|  | Included study(s) cited within | 15 |
| Geographic context | Region | North America |
|  | Country | Canada |
|  | Signatory City | Toronto |
|  | World Bank economic classification | High Income |
| Policy context | Is this intervention a policy itself or part of an over-arching policy? | Policy - regulatory |
|  | Timeframe for intervention | 2012 - 2021 |
|  | Related policy interventions (Regional, National, State, Local) | Rooted in the 2005 'Greater Toronto Area Agricultural Action Plan), applied to the City of Toronto and surrounding areas. |
| Policy Aims & Activities | Policy aim | Establish a collaboration to adopt a common plan which helps the food and farming sector remain viable in the face of land use pressures at the urban-rural interface, as well as infrastructure gaps, rising energy costs, and disjointed policy implementation. Five objectives: (i) to grow the food and farming cluster; (ii) to link food, farming and health through consumer education; (iii) to foster innovation to enhance competitiveness and sustainability; (iv) to enable the cluster to be competitive and profitable by aligning policy tools; and (v) to cultivate new approaches to supporting food and farming. |
|  | Description of actions | Establishment of the 'Golden Horseshoe Food and Farming Alliance', an innovative governance body bringing together 7 LGA stakeholders, civil society, researchers and food producers who meet 5 times per year. Activities included; expansion and relocation of Toronto's Food Business Incubator (assisting food start-ups), development of a strategy to connect barriers and opportunities for public food markets in Toronto linking consumers with local food supply, complete and maintain an inventory of production, processing, distribution and marketing infrastructure required to support the regional food system and attract investment to address identified needs, work with local food producers, retailers and manufacturers to explore international distribution opportunities to leverage the regions' cultural diversity, support and promote breakfast and lunch programs to promote healthy choices, work with food service providers at colleges and universities, design and offer primary and secondary education opportunities to engage students in health, nutrition and agriculture education, educate the community to consumer local healthy foods, market regional food and food products in health promotion campaigns, expand the regional food brand 'Foodland Ontario' through labelling (date of harvest, point of origin, processing activity), training for retail staff to accurately represent and promote local food, develop internships, apprenticeships, scholarship, etc to inspire students to take on careers in food and farming, promote local food to tourists to increase profitability, appoint a senior official in each LGA to assist farming and food entrepreneurs to navigate approval and regulatory processes,, align provincial and municipal taxes and fees to support profitability of the regional food and farming industry (e.g. long-term land rental agreements, financial incentives for innovative food processing), procurement policies for local food and beverages in public and broader public sector agencies. |
|  | Role of Local Government | Stakeholder representative, as one of 7 LGAs involved in the Alliance that manages this action plan. Active involvement in implementation and submission of plans to respective local authorities is part of the Terms of Reference. All 7 LGAs adopted this overarching action plan as their own local policy to streamline development, reporting, etc. Each municipality contributes funding ($30k CDN per annum) to employ the Executive Director and the part-funding of projects. |
|  | Targeted healthy and sustainable diet-related practice(s) | Connect with local food system  Support sustainable food production practices  Eat seasonally  Eat locally available foods |
|  | Targeted food system phase(s) | All |
| Considerations | Does the policy consider health? Y/N and how? | Yes - many of the activities consider health e.g. procurement policies to increase availability of healthy food for primary, secondary, university and college students. |
|  | Does the policy consider equity? Y/N and how? | Not stated |
|  | Does the policy consider the broader food system? Y/N and how? | Yes - this policy is aiming to protect the regional food system and takes a food systems approach in doing so. Consideration of every stage of the food supply chain. |
| Evaluation | Has evaluation been planned for? | Yes - the fifth objective includes considerable focus on research to pilot projects and monitor implementation success. Measurable targets and details of the approach to evaluation is not included in the action plan. |
|  | Data collection tools, target population | Not stated by IPES 2017 or the Action Plan. |
|  | Has the policy been effective? | Details of successful implementation are not stated in the included study nor in the action plan itself. |
| Policy Development | What evidence underpins the development of the policy? | The competition for land between urban development and agriculture brought together regional planners and agricultural groups in the 1990s. 2005 'Greater Toronto Area Agricultural Action Plan', which was applied to the City of Toronto and surrounding areas. It was instigated by farmers concerned about protection of agricultural land. |
|  | What type of evidence was used to develop the policy? | Expert opinion was considered in the development of this plan, including representation from government agencies, industry representatives, local governments, etc. |
|  | What was the process for using evidence to develop the policy? | In 2009 key stakeholders met (representing 5 of the 7 LGAs, farmers, grant-makers and the Mayors' Alliance) and agreed that subsequent plans should take a food systems approach to consider economic viability of agriculture in the area, including but not restricted to farmers. The Action Plan was developed over 3 phases; research, consultation and drafting. IPES (2017) describe the writing process to be long and labour-intensive due to economic development actors and planning professionals working on the task in different ways with language inconsistencies. |

| Citation | Name of Policy | **Toronto's Long-Term Waste Management Strategy (2016)** |
| --- | --- | --- |
|  | Reference | xxvi |
|  | Format and link or citation | The Food Waste Strategy is described within this [broader waste reduction strategy](https://www.toronto.ca/wp-content/uploads/2017/10/9803-Final-Long-Term-Waste-Management-Strategy.pdf) as implementing the Love Food Hate Waste Canada campaign, at a local level across the City of Toronto |
|  | Included study(s) cited within | 16 |
| Geographic context | Region | North America |
|  | Country | Canada |
|  | Signatory City | Toronto |
|  | World Bank economic classification | High Income |
| Policy context | Is this intervention a policy itself or part of an over-arching policy? | Love Food Hate Waste Canada is a Nation-wide campaign, facilitated at local government level |
|  | Timeframe for intervention | The Long-Term Waste Management Strategy was endorsed by Toronto City Council in 2016 with the intention to guide policy decisions for 30-50 years. |
|  | Related policy interventions (Regional, National, State, Local) | This food waste strategy is part of a broader ['Long Term Waste Reduction Strategy'](https://www.toronto.ca/services-payments/recycling-organics-garbage/long-term-waste-strategy/waste-reduction/) ([summary document](https://www.hdrinc.com/sites/default/files/2017-06/toronto-long-term-waste-management-strategy.pdf)). [Love Food Hate Waste Canada](https://lovefoodhatewaste.ca/), 'Waste Free Ontario Act'. |
| Policy Aims & Activities | Policy aim | Toronto's Food Waste Strategy was designed to reduce waste in two ways; (i) decrease the quantity of food that is being wasted (reduce by 34,000 tonnes/year by Year 10 of the strategy) (ii) increase the amount of food waste that is being captured for diversion (reduce the amount of Green Bin organics generated, reduce the need for new organics processing infrastructure and reduce the amount of garbage to be managed). The included study (Gorrie 2017) also describes that the strategy also aims to "support consumer participation in sustainable food systems". |
|  | Description of actions | Love Food Hate Waste Canada focuses on information and outreach programs to educate residents about the economic, environmental and social benefits of food waste reduction. Gorrie (2017) describes a range of activities included in the Waste Reduction Strategy; (i) Compulsory participation in the in household organics collection (weekly) with "Raccoon Resistant" green bins provided by Council, (ii) energy recovery from biogas at two anaerobic digesters, (iii) expansion and upgrade of an existing food digester which will double annual capacity to 60,000 tons of feedstock, (iv) education for residents about the benefits of reducing food waste, the value of food, suitable green bin contents, household food preparation strategies to minimise the generation of food waste, (v) Food handling certification support to enhance capacity to minimise food waste in households, schools, City divisions, agencies, corporations and commercial businesses. |
|  | Role of Local Government | City of Toronto acknowledged that food waste is a National issue and therefore partnered with National Zero Waste Council and other Canadian cities and food retailers by joining the Love Food Hate Waste Canada campaign. Implemented at the local government level. The strategy states that the waste strategy depends on successful partnerships eg. NGOs, other municipalities, universities, food-related organisations. |
|  | Targeted healthy and sustainable diet-related practice(s) | Minimise food waste |
|  | Targeted food system phase(s) | Consumption  Waste |
| Considerations | Does the policy consider health? Y/N and how? | Not stated |
|  | Does the policy consider equity? Y/N and how? | Yes - page 40 of the strategy document states that benefits of the strategy include; 'reducing the amount of food waste in households will result in potential household savings on grocery bills', 'increasing attention and participation in sustainable food movement and food security issues'. |
|  | Does the policy consider the broader food system? Y/N and how? | Yes - the strategy refers to the potential to divert organic waste back to feedstock, p40 of the strategy states that a benefit is 'reinforcing message of food production sustainability'. |
| Evaluation | Has evaluation been planned for? | Page 91 of the policy document describes the approach to measuring performance. Regarding the food waste reduction targets, the document states "Food waste reduction can be measured by performing waste audits that look at the composition of waste, specifically looking at the amount of wasted food, packaged food and food waste in all waste streams (i.e. garbage and Green Bin organics) |
|  | Data collection tools, target population | Food Waste Audits |
|  | Has the policy been effective? | The current Long-Term Waste Reduction Strategy refers to achievements from the 2014 strategy however none of the outcomes are specific to food waste. The included study for this review (Gorrie, 2017) reports that "almost 160,000 tons of source separated organics were collected for anaerobic digestion and/or composting in 2016. About 78% came from households." Gorrie describes Toronto as leading North America in organics recycling, and states that in 2016 "City divisions, agencies and corporations and commercial sources totalled 158,962 tons. More than 124,000 tons of that total came from households." |
| Policy Development | What evidence underpins the development of the policy? | Circular Economy is referenced throughout the Waste Strategy. “A circular economy… aims for the elimination of waste through the superior design of materials, products, systems and business models”. Towards the Circular Economy, Ellen MacArthur Foundation. |
|  | What type of evidence was used to develop the policy? | A Stakeholder Advisory Group was formed with 16 members representing City of Toronto, Social Planning Recycling Council of Ontario, Ontario Waste Management Association, Business Improvement Association, Catholic District School Board, Community Housing, Universities, Environmental Alliance, etc. Key Stakeholder Meetings were also held with representation from Aboriginal communities, tenants’ associations, non-profit organisations, rate-payer groups, etc. |
|  | What was the process for using evidence to develop the policy? | Page 7 -11 of the long-term policy document describes the participation of stakeholders as 'critical' to effective implementation of the strategy. "Thousands of participants from across the City have provided feedback, engaged in dialogue and accessed key project information through a variety of consultation and engagement activities". The consultation occurred over 3 phases; 'Building the foundation', 'Developing the Waste Strategy', and 'Documenting and deciding on this Waste Strategy'. 4 surveys with 3400 responses, 12 public consultation events with 4200 people, 6 project updates, 4 speaker sessions, 19 meetings with the Stakeholder Advisory Group, etc. |

| Citation | Name of Policy | **Regional Food System Action Plan Metro Vancouver (2016)** |
| --- | --- | --- |
|  | Reference | xxvii |
|  | Format and link or citation | [Action Plan](http://www.metrovancouver.org/services/regional-planning/PlanningPublications/RegionalFoodSystemActionPlan.pdf) |
|  | Included study(s) cited within | 8, 22 |
| Geographic context | Region | North America |
|  | Country | Canada |
|  | Signatory City | Vancouver |
|  | World Bank economic classification | High Income |
| Policy context | Is this intervention a policy itself or part of an over-arching policy? | Policy – regulatory |
|  | Timeframe for intervention | 2016-2020 |
|  | Related policy interventions (Regional, National, State, Local) | [Regional food system strategy Metro Vancouver](http://www.metrovancouver.org/services/regional-planning/PlanningPublications/RegionalFoodSystemStrategy.pdf) (2011) This strategy is linked to a number of existing plans e.g. Regional Growth Strategy, Integrated Solid Waste and Resource Management Plan, Affordable Housing Strategy, Drinking Water Management Plan, Parks and Greenways Plan. The strategy describes its' alignment with four key provincial initiatives; 'BC Agricultural Plan: Growing a healthy future for BC families (2008)', 'BC Climate Action Plan (2008)', 'Living Water Smart: BC's Water Plan (2008)', 'Healthy Eating Strategy (2007)' |
| Policy Aims & Activities | Policy aim | The overarching Regional Food System Strategy (2011)'s vision was to create "a sustainable, resilient and healthy food system that will contribute to the well-being of all residents and the economic prosperity of the region while conserving our ecological legacy". The strategy included 5 goals and 21 strategies. |
|  | Description of actions | This Action Plan was developed through consultation with staff from local governments who identified action that local governments were planning to undertake in the following 3-5 years. 160 actions to advance the 2011 strategy implementation and 18 new actions, all liked to the 5 key goals. (i) Increase Capacity to Produce Food Close to Home e.g. Protecting Agricultural Land (ii) Improve the Financial Viability of the Food Sector e.g. Increase direct marketing opportunities for local foods, revitalise processing, distribution and storage of local food through tax exemptions (ii) People Make Healthy and Sustainable Food Choices e.g. Enhance food literacy skills, enable residents to make healthy food choices (cooking classes and education-focused action items), communicate how food choices support sustainability (school policy, signage on farmland to promote local produce) (iv) Everyone has access to Healthy, Culturally Diverse and Affordable Food e.g. encouraging urban agriculture & food rescue efforts (v) A Food System Consistent with Ecological Health e.g. reduce waste (revise best before/used-by labelling, Love Food Hate Waste campaign), facilitate sustainable practices (parks programming around native plant foraging, seed saving/exchange, create healthy soil guidelines for urban farms, composting workshops) |
|  | Role of Local Government | Leadership/Ownership by Metro Vancouver. 'Metro Vancouver' is the entity (a political and corporate body operating under provincial legislation) which delivers 'regional services, planning and political leadership on behalf of 24 local authorities', including the City of Vancouver. |
|  | Targeted healthy and sustainable diet-related practice(s) | Variety (diversity)  Avoid over-consumption  Minimise food waste  Eat seasonally  Eat locally available foods  Connect with local food system  Support sustainable food production practices |
|  | Targeted food system phase(s) | All |
| Considerations | Does the policy consider health? Y/N and how? | Y - the 3rd goal is that 'People make healthy and sustainable food choices' - this includes a number of actions including; cooking/food literacy programs ('Table Matters', 'Tasty Connections'), workshops, implementing the Blue Dot movement to promote sustainable and healthy food choices, explore tax incentives to support healthy food recovery |
|  | Does the policy consider equity? Y/N and how? | Y - a number of actions within Goal 3 and Goal 4 aim to address inequities including: support the 'Golden Ears Feast' that provides cooking education programs for parents of low-income families, multi-lingual food resources, grants to increase nutritious meals for emergency food relief providers, support trips to local farms for refugee families, in-kind support for backyard sharing programs to partner homeowners with residents looking for urban gardening space |
|  | Does the policy consider the broader food system? Y/N and how? | Y - building capacity of primary producers is a focus, as well as incentivising the next generation of food producers. Other actions include: funding signage to promote u-pick and crop information on local farms, school learning gardens, neighbourhood champions program 'More peas please' teaching kids to grow food, 'Green Ambassadors' program for high school students, award agriculture awareness grants, promote local food procurement, increase community food markets, review development application processes to establish urban agriculture activities |
| Evaluation | Has evaluation been planned for? | The Action Plan states that review of the progress will be an "iterative and ongoing" process with annual reporting updates to respective regional and municipal decision-makers. In the 2011 Strategy document, the Appendix includes performance measures for each of the five goals eg. average age of farmers, proportion of residents eating 5 serves or more of fruit and vegetables, annual cost of a nutritious food basket. |
|  | Data collection tools, target population | The specifics of evaluation tools and approaches are not stated in the Action Plan. Each action item has an 'agency' listed against it who is responsible for implementation and likely evaluation. |
|  | Has the policy been effective? | Not stated |
| Policy Development | What evidence underpins the development of the policy? | Neither the Strategy or the Action Plan documents are referenced. |
|  | What type of evidence was used to develop the policy? | Expert opinion (citizen and key stakeholder consultation) |
|  | What was the process for using evidence to develop the policy? | Both the Strategy and the Action Plan describe community consultation processes. The Strategy states "hundreds of residents have attended public meetings to talk about agriculture and food issues". The Action Plan states that it was developed by "Metro Vancouver, member municipalities, the Tsawwassen First Nation and the BC Ministry of Agriculture. Input was also provided by regional and municipal Agricultural Advisory Committees, external stakeholders and a series of three Roundtable events hosted by Metro Vancouver in 2013 and 2014". |

| Citation | Name of Policy | **Chicago: GO TO 2040 Regional Comprehensive Plan Chicago (2010)** |
| --- | --- | --- |
|  | Reference | xxviii |
|  | Format and link or citation | [Action Plan](https://www.cmap.illinois.gov/documents/10180/17842/long_plan_FINAL_100610_web.pdf/1e1ff482-7013-4f5f-90d5-90d395087a53) |
|  | Included study(s) cited within | 8, 14 |
| Geographic context | Region | North America |
|  | Country | United States of America |
|  | Signatory City | Chicago |
|  | World Bank economic classification | High Income |
| Policy context | Is this intervention a policy itself or part of an over-arching policy? | Policy – regulatory |
|  | Timeframe for intervention | 2010 - 2040 |
|  | Related policy interventions (Regional, National, State, Local) | Promote sustainable local food' is one of the 12 recommendations included in the GO TO 2040 Regional Comprehensive Plan Chicago. The plan has four themes, and this food-related recommendation fits within the 'Liveable Communities' theme. The Federally funded 'Farm Bill', legislation passed every five years to guide national agriculture policy. This plan acknowledges a shift in federal policy towards supporting local food, with modest increase in the 2008 Farm Bill for production and access to local food (e.g. Farmers' Market Promotion Program or USDA "Food Desert" study) |
| Policy Aims & Activities | Policy aim | The 'promote sustainable local food' chapter has three objectives: (i) Facilitating sustainable local food production and processing, (ii) increasing access to safe, fresh, nutritious and affordable foods, (iii) raising awareness by providing data, research, training and information. |
|  | Description of actions | Actions are aligned with each of the three objectives and include the following (not an exhaustive list); Local governments to simplify and incentivise conversion of vacant and underutilised lots, spaces and rooftops for urban agriculture uses, local governments to maintain and improve current farmland protection programs, advocate for the procurement of local food by state institutions, support and expand alternative retail options (farmers' markets, food co-ops, on-site school programs, etc), work with emergency food relief providers to increase fresh food provision, improve data collection on local food distribution mechanisms (transportation and storage), encourage businesses and restaurants to support local food through menu and product labelling |
|  | Role of Local Government | Leadership/Ownership. Author of the plan is 'Chicago Metropolitan Agency for Planning' |
|  | Targeted healthy and sustainable diet-related practice(s) | More plant-based foods  Eat seasonally  Eat locally available foods  Connect with local food system  Support sustainable food production practices |
|  | Targeted food system phase(s) | All |
| Considerations | Does the policy consider health? Y/N and how? | Y - the plan aims to increase access to safe, fresh, nutritious and affordable food |
|  | Does the policy consider equity? Y/N and how? | Y - the plan describes mutual benefits of linking local food production programs with those which address food insecurity, and references the 2009 Hunger Strategy Report. Many of the actions described consider equity e.g. increasing availability of healthy food in existing food deserts (one of the Plan's targets is to reduce the percentage of people living in food deserts from 7% down to 0% by 2040) |
|  | Does the policy consider the broader food system? Y/N and how? | Y - this plan has a strong emphasis on strengthening the local food system from food production, processing, distribution, retail alternatives and consumption. |
| Evaluation | Has evaluation been planned for? | Approaches to evaluation have been documented throughout the plan, linked to specific action areas rather than an evaluation of the plan as a whole. The actions itemised for the sustainable local food chapter do not mention specific evaluation plans. |
|  | Data collection tools, target population | Illieva (2017) conducted an in-depth comparative analysis of the 2030 Agenda and sustainable food system strategies in New York, Philadelphia, Los Angeles, Chicago and Toronto. A systematic review and comparative analysis was conducted. |
|  | Has the policy been effective? | Not stated in this plan or any included studies from this review. This document is a 30-year plan, ending in 2040 |
| Policy Development | What evidence underpins the development of the policy? | The appendices links to the comprehensive [2009 Food Systems Report](https://www.cmap.illinois.gov/documents/10180/31446/012610+FOOD+SYSTEMS.pdf/67bf510e-62f8-4cec-ae58-c91f0212aef3), funded by the Chicago Community Trust and Affiliates |
|  | What type of evidence was used to develop the policy? | The 2009 Food Systems Report was developed with input from over 130 individuals and organisations via meetings over a 9-month period. Representatives included farmers, non-profits, private business, etc, facilitated by Chicago Food Policy Advisory Council and others. |
|  | What was the process for using evidence to develop the policy? | 47 strategy papers were researched and produced during the development stage. Public engagement was also part of the process in developing this intervention as outlined in the [Appendices](https://www.cmap.illinois.gov/about/2040/supporting-materials/appendices) |

| Citation | Name of Policy | **A Recipe for Healthy Places: Addressing the Intersection of Food and Obesity in Chicago (2013)** |
| --- | --- | --- |
|  | Reference | xxix |
|  | Format and link or citation | [Action Plan](https://www.chicago.gov/content/dam/city/depts/zlup/Sustainable_Development/Publications/Recipe_For_Healthy_Places/Recipe_for_Healthy_Places_Final.pdf) |
|  | Included study(s) cited within | 14 |
| Geographic context | Region | North America |
|  | Country | United States of America |
|  | Signatory City | Chicago |
|  | World Bank economic classification | High Income |
| Policy context | Is this intervention a policy itself or part of an over-arching policy? | Policy – regulatory |
|  | Timeframe for intervention | 2013 the policy was adopted. No end date stated |
|  | Related policy interventions (Regional, National, State, Local) | City of Chicago. A Recipe for Healthy Places: Addressing the Intersection of Food and Obesity in Chicago; City of Chicago Department of Housing and Economic Development: Chicago, IL, USA, 2013. |
| Policy Aims & Activities | Policy aim | The vision is stated: "Healthy food will play a central role in the lives of individuals, families and communities in Chicago, creating a stronger city. Backyard and community gardens, urban farms and food enterprises will support a culture that values fresh, nutritious food. Schools, community and faith-based organisations and government will promote and model healthy eating habits while respecting cultural and ethnic differences. Every neighbourhood will include places to buy fresh fruits and vegetables, and a food safety net will ensure that residents can eat well regardless of income." |
|  | Description of actions | The plan outlines six strategies with activities that link to environmental sustainability outcomes; (i) Build healthier neighbourhoods (involve public health professionals in community planning efforts to avoid obesogenic decisions, incorporate Health Impact Assessments into local planning and development projects to give policy-makers evidence-based recommendations), (ii) Grow food (identify and prepare public open spaces for urban agriculture purposes including food growing, job training and education activities, connect schools with community groups via gardening, protect soil health by establishing restrictions on illegal dumping), (iii) Expand Healthy Food Enterprises (target tax increment financing to develop healthy food businesses involved in production, processing and distribution of healthy food, enhance healthy food retail options especially in underserved areas), (iv) Strengthen the Food Safety Net (increase nutritional value and quality of food served at emergency food relief services and in school meals programs, expand the use of LINK cards, create technology to help people find food resources), (v) Serve Healthy Food and Beverages (add free water refilling stations to promote tap water consumption, criteria to limit fat, sugar and sodium in afterschool and childcare programs, establish food standards for vending machines and city vendors), (vi) Improve Eating Habits (adopt ethically and culturally appropriate curricula, engage grocery chains as partners by promoting point-of-sale messages, healthy food tastings and video messages at cash registers). |
|  | Role of Local Government | Leadership/Ownership. The policy work is led by the City of Chicago's Department of Housing and Economic Development and Department of Public Health. |
|  | Targeted healthy and sustainable diet-related practice(s) | Avoid over-consumption  Limit processed foods  More plant-based foods  Support sustainable food production practices  Promote safe tap water |
|  | Targeted food system phase(s) | Agricultural production  Distribution  Processing  Retail  Consumption |
| Considerations | Does the policy consider health? Y/N and how? | Yes - there is an emphasis on obesity prevention throughout the policy, with many references to other diet-related diseases |
|  | Does the policy consider equity? Y/N and how? | Yes - increasing healthy food retail options in underserved areas, strengthening the food safety net by setting high nutrition standards for emergency food relief and connecting people in need with the available services |
|  | Does the policy consider the broader food system? Y/N and how? | Yes - only in the sense of urban agriculture and shortening the supply chain by connecting consumers with growing their food - for dietary as well as educational and social outcomes |
| Evaluation | Has evaluation been planned for? | The plan mentions evaluation plans throughout (e.g. "collect data on urban food production") and suggests that this evaluation could be facilitated by University and non-profit partners over time. |
|  | Data collection tools, target population | The approach taken to evaluation (including tools and results) is not described in this policy document, nor in any of the included studies of this review. |
|  | Has the policy been effective? | This plan does not include details of any progress towards achieving the six strategies. It does however describe for each strategy, a range of examples where the work is underway in achieving the outlined strategy. |
| Policy Development | What evidence underpins the development of the policy? | The plan is referenced throughout and includes citations from peer-reviewed literature, policy documentation and other grey literature such as US Department of Agriculture and US Department of Health and Human Services data. |
|  | What type of evidence was used to develop the policy? | The Appendix outlines the planning process undertaken to develop this document. Evidence was obtained from research, expert opinion and peer reviewed literature. |
|  | What was the process for using evidence to develop the policy? | A pre-planning workshop was facilitated in 2011 which established values for the process of developing this policy, including to involve communities and organisations from across the city, especially those disproportionately affected by obesity. A number of approaches were taken to engage stakeholders in the policy-making process. This process included 26 public meetings held over 13 months to gather ideas and feedback on proposed strategies. Over 400 individuals participated in this process. |

| Citation | Name of Policy | **FoodWorks: a vision to improve NYCs food system (2010)** |
| --- | --- | --- |
|  | Reference | xxx |
|  | Format and link or citation | Policy Document - [Report](http://www.ngfn.org/resources/ngfn-database/knowledge/foodworks_fullreport_11_22_10.pdf) |
|  | Included study(s) cited within | 8, 14, 26 |
| Geographic context | Region | North America |
|  | Country | United States of America |
|  | Signatory City | New York City |
|  | World Bank economic classification | High Income |
| Policy context | Is this intervention a policy itself or part of an over-arching policy? | Policy – non-regulatory |
|  | Timeframe for intervention | First published in 2010 and then updated in 2013. No end time frame for this work plan has been stated. |
|  | Related policy interventions (Regional, National, State, Local) | PlaNYC is a blueprint for city-wide sustainability published in 2007, however food was missing from the narrative. Less than 3 years later, Foodworks NYC Report was released (a food sustainability blueprint). FRESH program, Green Cart program, Supplemental Nutrition Assistance Program (SNAP), Child Nutrition Act, WIC program, SNAP Education |
| Policy Aims & Activities | Policy aim | FoodWorks explores some of the ways in which the many pieces of the complex food system are interconnected, sets goals to support better choices, and presents a blueprint for some initial steps (a 59-point plan), both large and small, that can make the system stronger and more sustainable for generations to come. |
|  | Description of actions | AG PRODUCTION: (i) preserve and increase regional food production (ii) increase urban food production. PROCESSING: (iii) generate group and employment in the food manufacturing sector by making affordable space available and offering technical assistance (iv) increasing regional products being processed in and for NYC (v) reducing enviro impact from food processing. DISTRIBUTION: (vi) Improve food distribution in NYC by enhancing infrastructure, technology, transportation and planning. CONSUMPTION: (vii) create a healthier food environment (viii) strengthen safety net for hunger relief and nutrition programs (ix) improve nutrition of institutional meals (x) increase food literacy. POST-CONSUMPTION: (xi) decrease waste throughout the food system (xii) increase resource recapture in the food system. |
|  | Role of Local Government | LEADERSHIP/OWNERSHIP. The Mayor’s Office created a Food Policy Coordinator and inter-agency task force  at the request of the City Council. The City Council called on the Mayor  to create the New York City Food Policy Council. |
|  | Targeted healthy and sustainable diet-related practice(s) | Variety (diversity)  Avoid over-consumption  More plant-based foods  Eat seasonally  Eat locally available foods  Minimise food waste  Connect with local food system  Promote safe tap water  Prepare food at home with others  Support sustainable food production practices |
|  | Targeted food system phase(s) | All |
| Considerations | Does the policy consider health? Y/N and how? | Y - this policy document includes 'addressing both hunger and obesity', and 'improving the food environment' under its aims to 'improve public health', Many of the strategies proposed in this document address issues of physical health, and health equity. |
|  | Does the policy consider equity? Y/N and how? | Y - equity is considered throughout this report. Goal * specifically aims to strengthen the safety net of hunger and nutrition programs. This policy document also proposes to promote an increase of retail options with healthy foods in existing food deserts by including a strategy to 'aggressively market the FRESH Program'. Promotes increase in Co-ops offering affordable healthy food. Supporting an increase in healthy food available in convenience stores (eg. bodegas). Support for the Green Cart Program, GroceryWorks training program, SNAP, WIC, SNAP-Education |
|  | Does the policy consider the broader food system? Y/N and how? | Y - this policy document is broken up into the stages of the food supply chain in order to describe the current state of place and their proposed activities. It addresses agricultural production, processing, distribution, consumption and post-consumption phases of the food system. |
| Evaluation | Has evaluation been planned for? | The Report states that in developing the blueprint, they discovered fundamental gaps in the basic data available to measure food system progress. They state "therefore, the City Council will introduce legislation that will allow us to better understand the current state of our food system, monitor changes, and provide a foundation for future work". The report goes on to specify that the Reporting Bill will establish metrics across each phase of the food system to measure where food is procured from, any benefits to local processors, reach and quality of agency meals, progress towards eliminating hunger, etc. |
|  | Data collection tools, target population | Not stated. Ilieva (2017) however conducted an in-depth comparative analysis of the 2030 Agenda and sustainable food system strategies in New York, Philadelphia, Los Angeles, Chicago and Toronto. A systematic review and comparative analysis was conducted |
|  | Has the policy been effective? | Not stated. Ilieva (2017) discovered that Foodworks 2010's goals aligned with 16 of the 17 Sustainable Development Goals (missing 'Peace and Justice') |
| Policy Development | What evidence underpins the development of the policy? | A comprehensive review of the current state of play is presented in the Foodworks Report. |
|  | What type of evidence was used to develop the policy? | The reference list includes grey literature and some peer-reviewed research. |
|  | What was the process for using evidence to develop the policy? | Not stated in detail however the Acknowledgements page of the report states "This report would not be possible without the insights and contributions of hundreds of New Yorkers and organisations who work every day to improve our food system" and goes on to name 40 individuals, representing various agencies (eg. New York City Coalition Against Hunger, New York State Department of Agriculture and Markets, the Earth Institute at Columbia University) |

| Citation | Name of Policy | **New York City Food Standards (2011)** |
| --- | --- | --- |
|  | Reference | Xxxi |
|  | Format and link or citation | [Policy document](http://www.nyc.gov/html/dycd/downloads/pdf/2012/City_Agency_Food_Standards.pdf) |
|  | Included study(s) cited within | 7, 15, 18, 26 |
| Geographic context | Region | North America |
|  | Country | United States of America |
|  | Signatory City | New York City |
|  | World Bank economic classification | High Income |
| Policy context | Is this intervention a policy itself or part of an over-arching policy? | Policy – regulatory |
|  | Timeframe for intervention | Implemented 2008, revised 2011. End date not stated. |
|  | Related policy interventions (Regional, National, State, Local) | [Supporting documentation](https://www1.nyc.gov/site/doh/health/health-topics/healthy-workplaces.page), Mandatory standards for City Facilities and Vendors: 'Meals/Snacks Purchased and Served Standards', 'Meetings and Events Standards', 'Beverage Vending Machines Standards', 'Food Vending Machines Standards', 'Commissaries Standards'. Voluntary Adoption of the Standards: 'Cafeterias/Cafes Standards |
| Policy Aims & Activities | Policy aim | The New York City Food Standards aim to "reduce the prevalence of chronic disease, such as obesity, diabetes and cardiovascular disease, by improving dietary intake". New York City also recognizes the importance of "promoting a healthy and ecologically sustainable food system that conserves natural resources and supports long term public health goals". |
|  | Description of actions | The Standards set guidelines for any government facility where food is served, including vending machines, meetings/events and in commissaries at correctional facilities. They apply to more than 238 million meals and snacks served each year at the city’s facilities and programs, including schools, senior centres, homeless shelters, child care centres, after school programs, correctional facilities and public hospitals. They also apply to cafeterias/cafés on a voluntary basis. All food purchased or served by a City agency must meet the standards which stipulate acceptable nutrient values for each of the food groups. These are categorised into (i) purchased food, (ii) meals and snacks served, (iii) agency and population-specific standards and exceptions, and (iv) sustainability recommendations. City agencies are encouraged to offer these preferred products; (i) fruits and vegetables that are local, seasonal, or are grown by producers using low or no pesticides or an integrated pest management system, (ii) dairy products that are local or (iii) seafood that is sustainably raised or harvested. Agencies are also encouraged to procure locally grown/produced food products and educate their customers about these local foods through labelling and other mechanisms. |
|  | Role of Local Government | Leadership/Ownership. Development of the NYC Food Standards began in 2007 by a Food Procurement Workgroup with representatives from all City agencies involved in food purchasing or service. This workgroup engaged NYC Health Department as a technical advisor. The Food Standards became a citywide policy in 2008 as the first of its kind, applying to >3000 programs run by 12 City agencies. |
|  | Targeted healthy and sustainable diet-related practice(s) | Avoid over-consumption  More plant-based foods  Less animal-derived foods  Eat locally available foods  Support sustainable food production practices  Eat seasonally |
|  | Targeted food system phase(s) | Agricultural production  Distribution  Retail  Consumption |
| Considerations | Does the policy consider health? Y/N and how? | Y - The New York City Food Standards aim to reduce the prevalence of chronic disease, such as obesity, diabetes and cardiovascular disease, by improving dietary intake. |
|  | Does the policy consider equity? Y/N and how? | Y - Section iii stipulates exceptions to the standards for agencies working with population groups with special needs (eg. children, seniors, youth detention facilities, shelters, patients under therapeutic care). Agencies who purchase food for emergency food relief providers must follow the guidelines. However, food for disaster response is exempt. |
|  | Does the policy consider the broader food system? Y/N and how? | Y - "Procurement practices that support sustainable production systems that reduce the overall environmental impact of the food system, are aligned with long term public health goals and conserve natural resources that are needed to sustain the food supply in the long term" |
| Evaluation | Has evaluation been planned for? | The Food Standards document concludes with a statement that the suggestions will be evaluated and updated based on the "latest scientific research on nutrition, the relationship between human health and food production methods, and the sustainability of the food system". The specifics of an evaluation plan are not included in the Food Standards document. |
|  | Data collection tools, target population | Tsui et al (2015) conducted a comparative case study with three institutional settings involving document review and interviews (n not stated). Freudenberg et al (2018) assesses the use of annual food metrics reports to describe progress on implementing municipal food policies. |
|  | Has the policy been effective? | Tsui et al (2015)'s examination concluded that affordability and consumer preference are key factors affecting the provision of healthy foods in complex institutional settings. Freudenberg et al (2018) conclude that previous indicators and processes used to generate food metrics reports provide valuable data on the efficacy of policy implementation however some limitations exist. These annual reports could provide greater accuracy on policy implementation efficacy by introducing new indicators, incorporating additional data sources and broadening stakeholders involved in the process. |
| Policy Development | What evidence underpins the development of the policy? | The Food Standards document does not include this detail however Lederer et al (2014) describe the collaborative and evidence-based process [here](https://www-sciencedirect-com.ezproxy.lib.monash.edu.au/science/article/pii/S0749379713006338) |
|  | What type of evidence was used to develop the policy? | The Food Standards document advises that they have been developed based upon "agency feedback, review, and agreement", the details of which are not included in this document. |
|  | What was the process for using evidence to develop the policy? | The Food Standards document does not include this detail however Lederer et al (2014) describe the collaborative and evidence-based process [here](https://www-sciencedirect-com.ezproxy.lib.monash.edu.au/science/article/pii/S0749379713006338) |

| Citation | Name of Policy | **Healthy & Sustainable Food for San Francisco (2009)** |
| --- | --- | --- |
|  | Reference | Xxxii |
|  | Format and link or citation | [Policy - Executive Directive](https://sfgov.org/sffood/sites/default/files/Documents/sffood/MayorNewsomExecutiveDirectiveonHealthySustainableFood.pdf) |
|  | Included study(s) cited within | 8 |
| Geographic context | Region | North America |
|  | Country | United States of America |
|  | Signatory City | San Francisco |
|  | World Bank economic classification | High Income |
| Policy context | Is this intervention a policy itself or part of an over-arching policy? | Policy – regulatory |
|  | Timeframe for intervention | Foodshed Assessment was published in 2008, the Mayor called for the Healthy and Sustainable Food for San Francisco in 2009, the Food Policy Council was formed and implemented the action items. No end date is stated. |
|  | Related policy interventions (Regional, National, State, Local) | [Think globally – eat locally](https://4aa2dc132bb150caf1aa-7bb737f4349b47aa42dce777a72d5264.ssl.cf5.rackcdn.com/ThinkGloballyEatLocally-FinalReport8-23-08.pdf): San Francisco Foodshed Assessment' (2008) American Farmland Trust, Sustainable Agriculture Education and University of California Berkeley. Executive Directive 10-01 ['Healthy Food & Beverage Options in Vending Machines'](https://sfgov.org/sffood/sites/default/files/Documents/sffood/may_2010/vending_maching_policy.pdf) (2010) |
| Policy Aims & Activities | Policy aim | The Executive Directive states "In our vision, sustainable food systems ensure nutritious food for all people, shorten the distance between food consumers and producers, protect workers health and welfare, minimize environment impacts, and strengthen connections between urban and rural communities." |
|  | Description of actions | A new Food Policy Council was developed in response to the Executive whose initial task was to ensure the principles outlined in the Executive Directive were made into law. The action items are grouped into six themes; (1) Nutritional Standards e.g. vending machine Executive Directive (regulatory policy), nutrition guidelines for City contractors, procurement for City events. (2) Urban Agriculture e.g. land audit and educational/technical/financial support for urban gardeners. (3) Regional Food e.g. review of administrative processes to enable increased access to farmers' markets, food procurement ordinance to ensure a percentage of the City's food purchases support regional agricultural producers. (4) Hunger and Food Security e.g. addressing inadequate funding for emergency food relief programs. (5) Food Business e.g. incentivise healthy and sustainable food retail, promote sustainable food businesses. (6) Fisheries - no examples stated. |
|  | Role of Local Government | Leadership/Ownership. The Food Policy Council is lead by stakeholders from the Mayor's Office and has representatives from various local government departments as well as non-government and private business representatives (e.g. retail, restaurants, schools, urban agriculture) |
|  | Targeted healthy and sustainable diet-related practice(s) | Avoid over-consumption  Limit processed foods  Eat locally available foods  Eat seasonally  Connect with local food system  Support sustainable food production practices |
|  | Targeted food system phase(s) | Agricultural production  Retail  Consumption |
| Considerations | Does the policy consider health? Y/N and how? | Yes - significant focus is made on increasing access to healthy food eg. farmers markets, new 'Healthy Food and Beverage Options in Vending Machines' legislation, review of nutrition guidelines for contractors, food procurement ordinance, supporting healthy food in retail |
|  | Does the policy consider equity? Y/N and how? | Yes - the SF Food Stamp Program created a new website, launched remote sites, trained staff and other initiatives to increase enrolments and ultimately facilitate access to federal nutrition funds. School lunch program improvements. |
|  | Does the policy consider the broader food system? Y/N and how? | Yes - promotion of urban agriculture, procurement of regional foods, inclusion of healthy options in retail and restaurants, facilitation of access to farmers' markets. |
| Evaluation | Has evaluation been planned for? | The Food Policy Council published an [annual report](https://sfgov.org/sffood/sites/default/files/Documents/sffood/may_2010/vending_maching_policy.pdf) 12 months after the Mayor's Executive Directive |
|  | Data collection tools, target population | Evaluation methods are not described however each action item is evaluated independently by a range of key agencies, likely adopting a diverse range of data collection tools. |
|  | Has the policy been effective? | A number of key results are presented in the 12 month report (2010) which include (not an exhaustive list): nutrition standards for vending machines adopted, food businesses have healthy and sustainable food standards built into their leases, Airport introduced 'slow food' vendors, Food Security Task Force nutrition goals for city contractors including low cost and free dining rooms, 120 site reviewed in urban agricultural audit with 30 deemed potential options, local government support to transform 13 of these sites, funding secured for community gardens, city code for farmers' markets revised, Draft Food Procurement Ordinance developed, SF Wholesale Produce Market retained and expanded to promote regional produce, restaurant recognition program for environmental conservation, local food use, nutrition in underserved communities and safety standards. |
| Policy Development | What evidence underpins the development of the policy? | [Think globally – eat locally](https://4aa2dc132bb150caf1aa-7bb737f4349b47aa42dce777a72d5264.ssl.cf5.rackcdn.com/ThinkGloballyEatLocally-FinalReport8-23-08.pdf): San Francisco Foodshed Assessment' (2008) American Farmland Trust, Sustainable Agriculture Education and University of California Berkeley. |
|  | What type of evidence was used to develop the policy? | Expert opinion and benchmark policy interventions from the local area. |
|  | What was the process for using evidence to develop the policy? | The evaluation report document states that to develop the Executive Directive, the drafting team adopted a food systems approach to review all food-related policies previously passed in and around San Francisco. For each possible action item they asked a series of questions including; 'What food system area does this represent? Is there an existing policy that supports the proposed action? What agency(s) are responsible? What's the current status? What are the resources needed?' then a list of interventions was prioritised. All city agencies were consulted to determine feasibility. |

| Citation | Name of Policy | **San Francisco Zero Waste (2018)** |
| --- | --- | --- |
|  | Reference | Xxxiii |
|  | Format and link or citation | [Website](https://sfenvironment.org/zero-waste-legislation#waste-prevention) with linked policy documents |
|  | Included study(s) cited within | 15 |
| Geographic context | Region | North America |
|  | Country | United States of America |
|  | Signatory City | San Francisco |
|  | World Bank economic classification | High Income |
| Policy context | Is this intervention a policy itself or part of an over-arching policy? | Policy – regulatory |
|  | Timeframe for intervention | 2018-2030 |
|  | Related policy interventions (Regional, National, State, Local) | [Resolution Setting Zero Waste Date 2003](https://sfenvironment.org/sites/default/files/editor-uploads/zero_waste/pdf/resolutionzerowastedate.pdf), C40 Cities Advancing Towards Zero Waste [Declaration](https://www.c40.org/other/zero-waste-declaration), Mandatory Recycling and Composting [Ordinance](https://sfenvironment.org/sites/default/files/editor-uploads/zero_waste/pdf/resolutionzerowastedate.pdf) 2009, Single-use Food Ware Plastics, Toxics and Litter Reduction [Ordinance](https://sfbos.org/sites/default/files/o0294-18.pdf) 2018, Food Service Waste Reduction [Ordinance](https://sfenvironment.org/sites/default/files/editor-uploads/zero_waste/pdf/sfe_zw_food_service_waste_reduction_ordinance.pdf) 2006, Mayor's [Executive Order](https://sfenvironment.org/sites/default/files/editor-uploads/zero_waste/pdf/sfe_zw_executive_order_bottled_water.pdf.pdf) on Bottled Water 2007 |
| Policy Aims & Activities | Policy aim | This intervention aims to achieve two key targets; "(i) Reduce municipal solid waste generation by 15% by 2030 (reducing what goes to recycling, composting, and trash). (ii) Reduce disposal to landfill and incineration 50% by 2030 (in San Francisco this means reducing what goes in the black trash bins)." |
|  | Description of actions | This intervention encompasses a range of regulatory policies/ordinances, outreach and education. The city-led ordinances are grouped under five headings, some with more relevance to food than others. (i) Zero Waste e.g. signing the C40 Cities declaration (ii) Recycling, Composting, Trash e.g. mandatory requirement that everyone in SF separates their recyclables, compostable and landfill-bound trash (ordinance passed in 2009) with compliance audits conducted by authorities (ii) Producer Responsibility e.g. supporting the state-wide responsibility framework which ensures producers/suppliers are responsible for their waste generation, provides producer responsibility language to be used in city purchasing contracts, prohibiting Styrofoam or polystyrene use from food ware and instead requires the use of compostable or recyclable food ware, prohibits use of single-use food service ware, requires reusable beverage cups at events on City property, enables single-use plastic straws to be available upon request for people with disabilities and medical needs however single-use straws not available in self-serve areas, compostable plastic or recyclable paper must be used at all retail establishments (iv) Construction and Demolition e.g. new buildings must have drinking fountains to provide bottle-filling stations, disaster debris recycling, green building regulations (v) City Government e.g. restricting sale of packaged water, increase availability of drinking water in public areas, prohibit public funds being spent on bottled water. |
|  | Role of Local Government | This intervention is led by San Francisco's Department of the Environment. San Francisco consider themselves global leaders in waste reduction, based on their history of regulatory policy success. Their initial 2003 policy led to a 50% reducing in landfill disposal. San Francisco joined other C40 cities in the 'Waste to Resources'. |
|  | Targeted healthy and sustainable diet-related practice(s) | Minimise food waste  Promote safe tap water |
|  | Targeted food system phase(s) | Waste |
| Considerations | Does the policy consider health? Y/N and how? | No - not stated in the website content |
|  | Does the policy consider equity? Y/N and how? | No - not stated in the website content |
|  | Does the policy consider the broader food system? Y/N and how? | No - not stated in the website content |
| Evaluation | Has evaluation been planned for? | The commitment states that "progress will continue to be measured over the course of the next 10 years or more". The approach for evaluation has not been described however the 2 overarching goals are measurable. |
|  | Data collection tools, target population | Data collection tools and evaluation specifics are not described in the website or the included study. |
|  | Has the policy been effective? | IPES (2017) describe some indicators of success: "80% of waste is diverted, 300 tons of food scraps are collected daily, and 100 million fewer plastic bags are used each year" |
| Policy Development | What evidence underpins the development of the policy? | There is a long history of waste-reduction interventions in the City and County of San Francisco. Specific policy documents are not mentioned in the website or included study. |
|  | What type of evidence was used to develop the policy? | IPES 2017 states that the policy was originally developed "in the context of the city running out of landfill space and concern over toxic gases and groundwater contamination". |
|  | What was the process for using evidence to develop the policy? | IPES 2017 states that the policy was developed with wide cooperation across departments within the City and County of San Francisco, together with input from other organisations and citizens. No further detail regarding the consultation process is described in the website or included study. |

| Citation | Name of Policy | **Nairobi Urban Agriculture Promotion and Regulation Act (2015)** |
| --- | --- | --- |
|  | Reference | Xxxiv |
|  | Format and link or citation | [Regulation Act](http://kenyalaw.org/kl/fileadmin/pdfdownloads/Acts/NairobiCityCountyUrbarnAgriculturePromotionandRegulationAct2015.pdf) |
|  | Included study(s) cited within | 15 |
| Geographic context | Region | Sub-Saharan Africa |
|  | Country | Kenya |
|  | Signatory City | Nairobi |
|  | World Bank economic classification | Lower Middle Income |
| Policy context | Is this intervention a policy itself or part of an over-arching policy? | Policy - regulatory |
|  | Timeframe for intervention | Act enforced in 2015, no end date provided. |
|  | Related policy interventions (Regional, National, State, Local) | Harare Declaration on Urban and Peri-urban Agriculture (2003) - endorsement from Ministers of Kenya, Malawi, Swaziland, Tanzania and Zimbabwe to develop policies and instruments to enable urban agriculture. This catalysed development of the 2015 Act, a U-turn in policy after a time when local governments were reprimanding citizens for attempting to produce food on open land, believing it to be a threat to public health and land rights. Referred to in the Act: National Agriculture, Fisheries & Food Act (2013), Crops Act (2013) |
| Policy Aims & Activities | Policy aim | The Act aims to increase food security by enabling urban food production, to promote job creation, add value across the food chain, protect food safety and environmental health and regulate land (and other resources) access. |
|  | Description of actions | The Act describes key activities (not an exhaustive list); increase food security by empowering people and institutions to enable agricultural activities for subsistence and commercial purposes, develop peoples' capacities in food production, value addition, employment creation, etc; regulate access to land and water for urban agricultural purposes (prioritising high-density areas and informal settlements), protect food safety and public health, institutionalise administrative processes to access food resources (including organic waste); monitor social, economic, environmental conditions with consideration of authoritative research and best practice; enforce laws on issues regarding urban agriculture, facilitate job creation by promoting food production as an alternative source of income; ensure the inclusion of urban agriculture in the planning process of land use, zoning, food policy, marketing and market infrastructure, build capacity amongst farmers regarding sustainable livestock practices, pest management, organic compost, sustainable production of crops, vegetables, fruits, mushrooms, fish, etc; ensure urban agriculture efforts are adequately funded; promote animal welfare standards; promote traceability systems, prohibit pesticides and fertilisers. |
|  | Role of Local Government | Ownership/leadership. The act established the Nairobi City County Urban Agriculture Promotion Advisory Board to facilitate the work. Various roles of the County Government are stipulated throughout the Act, including prominent membership on the Executive Committee and the Nairobi City Council Urban Agriculture Promotion Advisory Board. |
|  | Targeted healthy and sustainable diet-related practice(s) | Connect with local food system  Support sustainable food production practices  Minimise food waste |
|  | Targeted food system phase(s) | Agricultural production  Distribution  Consumption  Waste |
| Considerations | Does the policy consider health? Y/N and how? | Y - public health is mentioned in terms of food safety and enabling institutional access to food resources |
|  | Does the policy consider equity? Y/N and how? | Y - priority for urban agriculture in areas with informal settlements (high density of low-income residents), job creation objectives in terms of commercialisation of food production |
|  | Does the policy consider the broader food system? Y/N and how? | Y - considers sustainable agricultural practices, use of pesticides/fertilizers, management of organic waste and manure, animal welfare standards |
| Evaluation | Has evaluation been planned for? | There is mention throughout the Act of the monitoring of achievements, and partnership with research institutions and best-practice research efforts. Measurable targets are not included in the Act itself. |
|  | Data collection tools, target population | Data collection tools or evaluation approaches are not described in the Act, nor are they detailed in the included study within this review (IPES, 2017) |
|  | Has the policy been effective? | IPES (2017) describe the successful policy-making process that led to the 2015 Act. They describe a participatory policy process, involving many stakeholders (farmers given a dominant voice) and the enabling power of having supportive civil servants. |
| Policy Development | What evidence underpins the development of the policy? | The policy-making process considered expertise from National and State documentation. |
|  | What type of evidence was used to develop the policy? | Citizen support; expert opinion (farmers and government officials - reassigned to Nairobi city council) |
|  | What was the process for using evidence to develop the policy? | The Nairobi and Environs Food Security, Agriculture and Livestock Forum (NEFSALF) formed in 2004 from an initial open meeting of everyone interested in growing food in Nairobi. This forum allowed the farmers to 'have a voice' (IPES, 2017). |

| Citation | Name of Policy | **Dakar Micro-gardens Programme (2006)** |
| --- | --- | --- |
|  | Reference | Xxxv |
|  | Format and link or citation | [Webpage](https://www.c40.com/case_studies/micro-gardening-in-dakar-alleviates-poverty-hunger-and-food-insecurity) - case study as part of FAO's website |
|  | Included study(s) cited within | 15 |
| Geographic context | Region | Sub-Saharan Africa |
|  | Country | Senegal |
|  | Signatory City | Dakar |
|  | World Bank economic classification | Lower Middle Income |
| Policy context | Is this intervention a policy itself or part of an over-arching policy? | Part of the 2019-2023 Senegalese Country Programming Framework (CPP) - in partnership with FAO. Regulatory components. |
|  | Timeframe for intervention | Project commenced May 2006. End date is not stated however webpage published June 2019 with current implementation details. |
|  | Related policy interventions (Regional, National, State, Local) | Part of the 2019-2023 Senegalese Country Programming Framework (CPP) - in partnership with FAO. Regulatory components. |
| Policy Aims & Activities | Policy aim | 2030 Agenda, Regional Initiatives, 2013-2017 CPP, UN Development Assistance Framework (UNDAF 2012-2018) for Senegal |
|  | Description of actions | Contribute to City of Dakar's concept of a 'green city' in an effort to adapt to the consequences of climate change, scarcity of agricultural land, and benefit from the social and health benefits of urban agriculture (increasing food access, economic opportunities and re-use of waste materials). |
|  | Role of Local Government | Activities within this programme include; installation of 12 training and demonstration centres, train-the-trainer model (24 facilitators educated in micro-gardening - 18 women and 6 men), 26 elementary school micro-gardens formed, catering for vulnerable population sub-groups, fruit/veg provision for correctional facilities. |
|  | Targeted healthy and sustainable diet-related practice(s) | Variety (diversity)  More plant-based foods  Eat seasonally  Eat locally available foods  Minimise food waste  Connect with local food system  Support sustainable food production practices |
|  | Targeted food system phase(s) | Agricultural production  Distribution  Retail  Consumption  Waste |
| Considerations | Does the policy consider health? Y/N and how? | Y - fruit and vegetables grown is given to 'at risk' community members including women with diabetes, anaemia, obesity and kids with disabilities, elderly |
|  | Does the policy consider equity? Y/N and how? | Y - providing food and income for 4000 families |
|  | Does the policy consider the broader food system? Y/N and how? | Y - this program implements a soil-less system of agricultural production, using locally available substrates (waste materials) such as crop trays made from wooden pallets, recovered peanut husks and rice straw, old utensils/tyres/bottles all reused |
| Evaluation | Has evaluation been planned for? | The webpage states that the next stage of the programme is to integrate it into the city's master plan. The data presented in IPES (2017) and on the webpage demonstrates that some data has been collected throughout the implementation period. |
|  | Data collection tools, target population | Not stated. It appears data is being recorded about how many people eat the food produces at these micro-gardens, who is benefiting nutritionally and financially from this and the partnerships formed (e.g. procurement opportunities) |
|  | Has the policy been effective? | 9694 beneficiaries (83% women, 17% men), increased yields from micro-gardening than traditional gardening, increased consumption of fruit and vegetables amongst students, young people with disabilities, women with diabetes/anaemia/obesity, elderly, detainees, source of food and income for 4000 families |
| Policy Development | What evidence underpins the development of the policy? | Not stated |
|  | What type of evidence was used to develop the policy? | Not stated |
|  | What was the process for using evidence to develop the policy? | Not stated |

| Citation | Name of Policy | **Urban Agriculture Policy (2007)** |
| --- | --- | --- |
|  | Reference | Xxxvi |
|  | Format and link or citation | [Policy document](https://ubwp.buffalo.edu/foodlab/wp-content/uploads/sites/68/2019/03/Urban-Agricultural-Policy-for-the-City-of-Cape-Town-approved-on-07-December-2006-Cape-Town-South-Africa.pdf) |
|  | Included study(s) cited within | 15 |
| Geographic context | Region | Sub-Saharan Africa |
|  | Country | South Africa |
|  | Signatory City | Cape Town |
|  | World Bank economic classification | Upper Middle Income |
| Policy context | Is this intervention a policy itself or part of an over-arching policy? | Policy - regulatory |
|  | Timeframe for intervention | Commenced 2007 with no end date provided |
|  | Related policy interventions (Regional, National, State, Local) | Integrated Environment Management Plan, Economic Development Strategy, Poverty Alleviation Strategy, Water Services Development Plan |
| Policy Aims & Activities | Policy aim | The policy has four key objectives; "(i) To enable the poorest of the poor to utilize urban agriculture as an element of their survival strategy (household food security), (ii) To enable people to create commercially sustainable economic opportunities through urban agriculture (jobs and income), (iii) To enable previously disadvantaged people to participate in the land redistribution for agricultural development programme (redress imbalances), (iv) To facilitate human resources development (technical, business and social skills training)" |
|  | Description of actions | 9 strategic imperatives; (i) Include urban agriculture in land use management and physical planning e.g. alterations to municipal land planning processes (land use plans, zoning schemes) (ii) Create linkages with other strategies e.g. internal interventions (poverty alleviation strategy, new housing settlements, local economic development strategies) and externally (integrated nutrition programme of the National Departments of Health and Education, National Department of Land Affairs), (iii) Establish urban agricultural consultative forums e.g. plenary sessions for all relevant stakeholders, Urban Agriculture Association, Intergovernmental Committee (key representatives from the City, Provincial Department of Agriculture), (iv) Build strategic partnerships (v) Release municipal land for urban agricultural purposes e.g. City will acquire commonage land from grant funding which they can lease to emerging city farmers through a permit system (vi) Provide subsidised water for vulnerable groups e.g. 10 kilolitre of water per month for free per institution or group of women, elderly, HIV/Aids facilities, soup kitchens, schools, orphanages. (vii) Specific strategy for livestock keeping - protect public health and safety by moving livestock to urban fringes. (viii) Introduce a support program for urban agriculture e.g. assistance to access land, basic infrastructure, capacity and skills development. (ix) Integrate urban agriculture into commercial agricultural industry e.g. urban farmers use same suppliers, markets, research bodies as commercial farmers. |
|  | Role of Local Government | Developed by the Economic and Human Development Departments. Implemented by the City of Cape Town's Urban Agricultural Unit. The City acts as a facilitator to enable urban agriculture to occur e.g. reducing 'red tape', introducing and exercising regulations, providing land, construction of necessary infrastructure, etc. |
|  | Targeted healthy and sustainable diet-related practice(s) | Connect with local food system  More plant-based foods  Eat seasonally  Eat locally available foods  Support sustainable food production practices |
|  | Targeted food system phase(s) | Agricultural production  Consumption  Distribution |
| Considerations | Does the policy consider health? Y/N and how? | Y - the policy aims to make urban agriculture more accessible to people experiencing food insecurity based on evidence that their nutritional status is the most disadvantaged. By increasing access to locally produced fresh food, health outcomes will improve. |
|  | Does the policy consider equity? Y/N and how? | Y - the City of Cape Town believes that urban agriculture can contribute to poverty alleviation by improving household food security, increasing nutritional status of people experiencing disadvantage, creation of jobs and income generation. Beneficiary groups are targeted based on vulnerability, alignment with food security-related National, provincial and local policies and strategies. |
|  | Does the policy consider the broader food system? Y/N and how? | Y - this intervention aims to promote a thriving urban agriculture industry that connects with commercial farmers and feeds into their existing systems |
| Evaluation | Has evaluation been planned for? | Monitoring and evaluation is described in the policy document as one of the key implementation actions "Develop system and procedure to monitor production output' and 'Design instrument to evaluate impact of urban agriculture', both with a lead agent listed as City of Cape Town's Department of Agriculture. Measurable targets are not listed in the policy document itself. |
|  | Data collection tools, target population | Data collection tools or evaluation approaches are not described in the policy document, nor are they detailed in the included study within this review (IPES, 2017) |
|  | Has the policy been effective? | IPES (2017) state that 'Assistance has been provided to between 50 and 60 food gardens each year, with provision of technical business skills training'. No further evaluation outcomes are included in the policy document nor the included study. |
| Policy Development | What evidence underpins the development of the policy? | This detail is not included in the policy document or the included study (IPES, 2017). |
|  | What type of evidence was used to develop the policy? | This detail is not included in the policy document or the included study (IPES, 2017). |
|  | What was the process for using evidence to develop the policy? | This detail is not included in the policy document or the included study (IPES, 2017). |

# Reference List of Included Studies

1. Timpanaro, G., et al., Urban agriculture as a tool for sustainable social recovery of metropolitan slum area in Italy: case Catania*.* 2018(1215): p. 315-318.
2. Schwartzman, F., et al., Background and elements of the linkage between the Brazilian school feeding program and family farming*.* 2017. 33(12): p. e00099816.
3. Säumel, I., Reddy, S., and Wachtel, T., Edible City Solutions—One Step Further to Foster Social Resilience through Enhanced Socio-Cultural Ecosystem Services in Cities*.* 2019. 11(4).
4. Sanyé-Mengual, E., et al., Evaluating the current state of rooftop agriculture in Western Europe: categories and implementation constraints*.* 2018(1215): p. 325-332.
5. Mulligan, K., et al., Toronto Municipal Staff and Policy-makers' Views on Urban Agriculture and Health: A Qualitative Study*.* 2018: p. 133-156.
6. Lazzarini, L., The role of planning in shaping better urban-rural relationships in Bristol City Region*.* 2018. 71: p. 311-319.
7. Tsui, E.K., et al., Institutional food as a lever for improving health in cities: the case of New York City*.* 2015. 129(4): p. 303-9.
8. Sonnino, R., The new geography of food security: exploring the potential of urban food strategies*.* 2016. 182(2): p. 190-200.
9. Smith, J., et al., Balancing competing policy demands: the case of sustainable public sector food procurement*.* 2016. 112: p. 249-256.
10. Nogueira, R.M., et al., Sixty years of the National Food Program in Brazil*.* 2016. 29(2): p. 253-267.
11. Neto, B. and Gama Caldas, M., The use of green criteria in the public procurement of food products and catering services: a review of EU schemes*.* 2017. 20(5): p. 1905-1933.
12. Olsson, E., et al., Peri-Urban Food Production and Its Relation to Urban Resilience*.* 2016. 8(12).
13. Moragues-Faus, A. and Morgan, K., Reframing the foodscape: the emergent world of urban food policy*.* 2015. 47(7): p. 1558-1573.
14. Ilieva, R.T., Urban Food Systems Strategies: A Promising Tool for Implementing the SDGs in Practice †*.* 2017. 9(10).
15. Hawkes, C. and Halliday, J. What makes urban food policy happen? Insights from five case studies. 2017. International Panel of Experts on Sustainable Food Systems.
16. Gorrie, P., Residential Organics Diversion in Toronto, in BioCycle. 2017: Canada.
17. Goncalves, H., et al., Family farming products on menus in school feeding: a partnership for promoting healthy eating. 2015. 45(12): p. 2267-73.
18. Freudenberg, N., Willingham, C., and Cohen, N., The Role of Metrics in Food Policy: Lessons from a Decade of Experience in New York City*.* 2018: p. 191-209.
19. Fesenfeld, L.P., Governing Urban Food Systems in the Long Run: Comparing Best Practices in Sustainable Food Procurement Regulations*.* 2016. 25(4): p. 260-270.
20. Dubbeling, M., et al., Assessing and Planning Sustainable City Region Food Systems: Insights from Two Latin American Cities*.* 2017. 9(8).
21. Crivits, M., et al., Four Perspectives of Sustainability Applied to the Local Food Strategy of Ghent (Belgium): Need for a Cycle of Democratic Participation? 2016. 8(1).
22. Collé, M., Daniel, A.C., and Aubry, C., Call for projects “Parisculteurs”: catalyst for urban agriculture development on rooftops in Paris*.* 2018(1215): p. 147-152.
23. Cerutti, A.K., et al., Modelling, assessing, and ranking public procurement options for a climate-friendly catering service*.* 2017. 23(1): p. 95-115.
24. Camps-Calvet, M., et al., Ecosystem services provided by urban gardens in Barcelona, Spain: Insights for policy & planning*.* 2016. 62: p.14-23.
25. Bügel, Public procurement as a means to link sustainable production with diet and lifestyle - the new nordic diet way 2017. 71 Suppl 2(21st International Congress of Nutrition. Buenos Aires, Argentina, October 15-20, 2017): p. 1-1433.
26. Campbell, L.K., Getting farming on the agenda: Planning, policymaking, and governance practices of urban agriculture in New York City*.* 2016. 19: p. 295-305.
27. Balzaretti, C.M., et al., Improving the overall sustainability of the school meal chain: the role of portion sizes*.* 2020. 25(1): p. 107-116.

1. Food and Agriculture Organisation and World Health Organisation. Sustainable healthy diets - Guiding principles. 2019. Rome. [↑](#footnote-ref-1)
2. Willett, W., et al., Food in the Anthropocene: the EAT–Lancet Commission on healthy diets from sustainable food systems. Lancet, 2019. 393(10170): p. 447-492. [↑](#footnote-ref-2)
3. World Health Organization, A healthy diet sustainably produced - information sheet. 2018. [↑](#footnote-ref-3)
4. United Nations Standing Committee on Nutrition. UNSCN Discussion Paper: Sustainable diets for healthy people and a healthy planet. 2017. Rome. [↑](#footnote-ref-4)
5. High Level Panel of Experts. Nutrition and food systems. A report by the High-Level Panel of Experts on Food Security and Nutrition of the Committee on World Food Security. 2017. Rome. [↑](#footnote-ref-5)
6. United Nations. United Nations Decade of Action on Nutrition (2016-2025). 2016. General Assembly on 1 April 2016. [↑](#footnote-ref-6)
7. Fischer, C. and Garnett, T. Plates, pyramids and planets. Developments in national healthy and sustainable dietary guidelines: a state of play assessment. 2016. FAO and the Food Climate Research Network at The University of Oxford. [↑](#footnote-ref-7)
8. Burlingame, B. and Dernini, S. Sustainable Diets and Biodiversity: Directions and Solutions for Policy, Research and Action. Proceedings of the International Scientific Symposium 2012. FAO Headquarters, Rome. [↑](#footnote-ref-8)
